# Identification of QTN-by-environment interactions and their candidate genes for soybean seed oil-related traits using 3VmrMLM

**DOI:** 10.3389/fpls.2022.1096457

**Published:** 2022-12-12

**Authors:** Jian-Fang Zuo, Ying Chen, Chao Ge, Jin-Yang Liu, Yuan-Ming Zhang

**Affiliations:** ^1^College of Plant Science and Technology, Huazhong Agricultural University, Wuhan, China; ^2^Jiangsu Key Laboratory for Horticultural Crop Genetic Improvement, Institute of Industrial Crops, Jiangsu Academy of Agricultural Sciences, Nanjing, China

**Keywords:** QTN-by-environment interaction, gene-by-environment interaction, 3VmrMLM, genome-wide association study, seed oil-related trait, soybean

## Abstract

**Introduction:**

Although seed oil content and its fatty acid compositions in soybean were affected by environment, QTN-by-environment (QEIs) and gene-by-environment interactions (GEIs) were rarely reported in genome-wide association studies.

**Methods:**

The 3VmrMLM method was used to associate the trait phenotypes, measured in five to seven environments, of 286 soybean accessions with 106,013 SNPs for detecting QTNs and QEIs.

**Results:**

Seven oil metabolism genes (*GmSACPD-A, GmSACPD-B, GmbZIP123, GmSWEET39, GmFATB1A, GmDGAT2D*, and *GmDGAT1B*) around 598 QTNs and one oil metabolism gene GmFATB2B around 54 QEIs were verified in previous studies; 76 candidate genes and 66 candidate GEIs were predicted to be associated with these traits, in which 5 genes around QEIs were verified in other species to participate in oil metabolism, and had differential expression across environments. These genes were found to be related to soybean seed oil content in haplotype analysis. In addition, most candidate GEIs were co-expressed with drought response genes in co-expression network, and three KEGG pathways which respond to drought were enriched under drought stress rather than control condition; six candidate genes were hub genes in the co-expression networks under drought stress.

**Discussion:**

The above results indicated that GEIs, together with drought response genes in co-expression network, may respond to drought, and play important roles in regulating seed oil-related traits together with oil metabolism genes. These results provide important information for genetic basis, molecular mechanisms, and soybean breeding for seed oil-related traits.

## Introduction

Soybean (*Glycine max* L. Merr.) is one of the most important oil crops (Zhang et al., 2004), contributing 58% of the world oilseed production and 28% of the world vegetable oil consumption in 2020 (http://www.soystats.com). Seed oil content (OIL) account for approximately 18 ~ 20% of dry seed weight in soybean, and it is mainly composed of five fatty acids (FAs): palmitic acid (PA), stearic acid (SA), oleic acid (OA), linoleic acid (LA), and linolenic acid (LNA), which are present at approximate concentrations of 12%, 3%, 26%, 52%, and 7% in bred soybeans, respectively (Zuo et al., 2022).

OIL in soybean is a complex quantitative trait, controlled by a few major genes and a series of polygenes, and affected by environment (Burton, 1987). The influence of environmental factors on OIL and FA compositions in soybean has been reported, including temperature (Dornbos and Mullen, 1992; Gibson and Mullen, 1996; Piper and Boote, 1999), drought (Dornbos and Mullen, 1992), and sunshine duration (Song et al., 2016). Seed oil content increased with the increase of temperature, approached a maximum at a mean temperature of 28°C, and decreased when temperatures exceeded these levels (Gibson and Mullen, 1996; Piper and Boote, 1999). Temperature strongly influences FA biosynthesis during ripening period (Werteker et al., 2010; Song et al., 2018). Higher temperatures during seed-filling stage increased OA content and decreased LA and LNA content, while PA and SA were relatively stable to environmental change (Dornbos and Mullen, 1992; Gibson and Mullen, 1996). Increased drought stress could decrease seed oil content, and severe drought stress during seed-fill stage could lead to up to 12.4% oil decrease (Dornbos and Mullen, 1992). Sunshine duration correlated positively with PA, SA, and LNA levels and negatively with OA level (Song et al., 2016). Genotype × environment interactions play a more and more important role in crop production. Primomo et al. (2002) found that year effect has the largest impact on all fatty acid levels, location effect is significant only for OA and LNA, and genotype × year interaction effect was significant for all fatty acids whereas genotype × location and genotype × year × location interaction effects were significant only for OA, LA, and LNA. Understanding of genotype × environment interactions is needed to allow breeders to better predict the phenotypes in given environments.

To date, more than 300 quantitative trait loci (QTLs) for seed oil content and more than 200 QTLs for seed fatty acid contents have been identified across all the 20 chromosomes in soybean genome using genome-wide association study (GWAS) and QTL mapping approaches (SoyBase, https://soybase.org). In previous studies, a lot of key functional genes have been reported to control seed oil content and fatty acid composition, such as *B1* (Zhang et al., 2018), *GmOLEO1* (Zhang et al., 2019), *GmPDAT* (Liu et al., 2020b), *GmSWEET39* (Miao et al., 2020; Zhang et al., 2020), *GmZF351* (Li et al., 2017), *GmDREBL* (Zhang et al., 2016), *FAD2-1A*, and *FAD2-1A* (Haun et al., 2014). Several important transcription factors have been identified as well, such as *GmDof4* and *GmDof11* (Wang et al., 2007), *GmMYB73* (Liu et al., 2014), *GmNFYA* (Lu et al., 2016), and *GmbZIP123* (Song et al., 2013). These studies have increased our understanding for soybean lipid metabolism mechanism, and provided useful information for the improvement of soybean seed oil-related traits.

In recent years, some QTN × environment interactions (QEIs) for seed oil-related traits in soybean have been reported. At early stage, seven QTLs for LNA have been identified by Han et al. (2011) to have significant additive × environment interaction effects. Recently, approximately thirty QTLs for soybean seed oil-related traits have been identified by Teng et al. (2017); Xia et al. (2017); Karikari et al. (2019), and Liu et al. (2020a) to have additive effects and/or additive × environment interaction effects. The identification of QEIs can be used to mine elite genes suitable for different environment, and provide gene sources for breeding of high oil content soybean accessions under extreme environment. However, these QEIs are still far from enough, especially, few candidate gene-by-environment interactions (GEIs) have been reported.

Some soybean genes have been reported to respond with environment factors. For examples, *GmAdh2* (Zhang et al., 2018), *GmMYB118* (Du et al., 2018), *GmCAMTA12* (Noman et al., 2019), *GmWRKY54* (Wei et al., 2019), *LHY1a*, and *LHY1b* (Wang et al., 2021) controlled drought response. Several oil metabolism genes had been reported to respond with environments, e.g., *GmPLDα1* affected lipid metabolism under high temperature and humidity conditions (Zhang et al., 2019). Mutation of *GmFAD3* resulted in lower linolenic acid content (from 7% to 10%) (Chappell and Bilyeu, 2007)*. GmFAD3A* can enhance cold tolerance in rice through the accumulation of proline content, the synergistic increase of the antioxidant enzymes activity, which finally ameliorated the oxidative damage (Wang et al., 2019). Loss of *SACPD* induced a variety of defense-related phenotypes and confers resistance to multiple pathogens in soybean (Kachroo et al., 2008). However, these seed oil-related trait genes that interact with environments were very limited.

To address the above issue, in this study the phenotypes of seed oil-related traits of 286 soybean accessions in five to seven environments were used to associate with 106,013 SNP markers for identifying QTNs and QEIs for seed oil-related traits using 3VmrMLM (Li et al., 2022b). Around these QTNs and QEIs, the genes, reported in previous studies and verified *via* molecular biological experiments, and candidate genes and GEIs were mined using multi-omics approaches. The results will provide important information for genetic foundation, function identification, molecular mechanism, and molecular breeding of seed oil-related traits in soybean.

## Materials and methods

### Genetic population

As described in Zhou et al. (2015a), a total of 286 soybean accessions, including 14 wild, 153 landrace, and 119 bred soybeans were obtained from six geographic regions of China, and planted in three-row plots in a randomized complete block design at the Jiangpu experimental station of Nanjing Agricultural University in 2011, 2012, 2014, 2015, and 2016 (NJ2011, NJ2012, NJ2014, NJ2015, and NJ2016 datasets), and at the Wuhan experimental stations of Huazhong Agricultural University in 2014 and 2015 (WH2014 and WH2015 datasets), respectively.

### SNP genotypes of 286 soybean accessions

Through resequencing of 286 soybean accessions using RAD-seq approach, a total of 106,013 high-quality SNPs were obtained, which had been described in our previous study of Zhou et al. (2015a).

### Phenotypes of six seed oil-related traits in 286 soybean accessions

In the genetic population, the plots were 1.5 m wide and 2 m long, and approximately 15 plants were planted in each row. Five plants in the middle row for each line were randomly harvested, and the seeds were prepared for the measurement of six seed oil-related traits (Zhou et al., 2016): PA, SA, OA, LA, LNA, and OIL, while the phenotypes for these traits of 286 soybean accessions in NJ2011, NJ2012, NJ2014, NJ2015, NJ2016, WH2014, and WH2015 were described in our previous studies of Zhou et al. (2016); Liu et al. (2020b), and Zuo et al. (2022).

### Statistical analysis

The statistical analysis and figure visualization in this study were conducted using R software. Phenotypic characteristics of six oil-related traits were analyzed using R package *psych*. Two-way analysis of variance (ANOVA) was performed to determine the significances for genotypes and genotype × environment interaction using R function *aov*. Correlation analysis among six seed oil-related traits was conducted and visualized using R package *GGally*.

### Identification of significant QTNs for seed oil-related traits in 286 soybean accessions

The single environment module of the IIIVmrMLM software (Li et al., 2022a) for the 3VmrMLM method (Li et al., 2022b) was used to identify QTNs for six seed oil-related traits in each environment, while its multiple environment module was used to detect QTNs and QEIs for the above traits. The kinship matrix K was calculated by the IIIVmrMLM software. As described in Zhou et al. (2015a), the number of optimum subgroups was four, and the Q matrix was calculated by the STRUCTURE 2.3.4 software (Hubisz et al., 2009). The critical *P*-value and LOD score were set as 0.05/*m* and 3.0, respectively, for significant and suggested QTNs, where *m* is the number of markers (Li et al., 2022b).

### Expression levels of candidate genes for seed oil-related traits

Here there were three transcriptome datasets available to conduct high expression analyses. The first transcriptome datasets were downloaded from the Gene Expression Omnibus database (http://www.ncbi.nlm.nih.gov/geo/query/acc.cgi?acc=GSE42871; Jones and Vodkin, 2013) and used to detect high expression genes at seed oil accumulation stages, in which their expression levels at these stages from 5 ~ 6 mg to 400 ~ 500 mg are higher than the average at all the seven seed development stages (Zhang et al., 2016). These stages included whole seed 4 days after flowering (DAF), whole seed 12 ~ 14 DAF, whole seed 22 ~ 24 DAF, whole seed 5 ~ 6 mg in weight, cotyledons 100 ~ 200 mg in weight, cotyledons 400 ~ 500 mg in weight, and dry whole seed. The second transcriptome datasets (Machado et al., 2020) were derived from the re-analyses of the first transcriptome datasets excluded the fifth and sixth stages and download from a user-friendly web interface at https://venanciogroup.uenf.br/resources/. The third transcriptome datasets at seed_10DAF, seed_14DAF, seed_21DAF, seed_25DAF, seed_28DAF, seed_35DAF, and seed_42DAF were downloaded from SoyBase (http://soybase.org; Severin et al., 2010). If one gene was highly expressed in at least two datasets, this gene was considered to be highly expressed in this study.

The gene expressional levels of two wild, two landrace, and two bred soybeans at 15, 25, 35, and 55 (DAF), described by Niu et al. (2020) and Liu et al. (2020a), were used to determine differential expression genes between wild and landrace soybeans and between landrace and bred soybeans using the DEGseq package (Wang et al., 2010) at the 0.001 probability level.

### SNP variants and haplotype analysis

Marker genotypes of 302 soybean accessions in Zhou et al. (2015b) were downloaded from https://figshare.com/articles/Soybean_resequencing_Project/1176133 (Figshare database). Using the downloaded genotypes, all the SNPs within each candidate gene and its 2 kb upstream were mined.

The genome sequences(glyma.Wm82.gnm1.FCtY.genome_main.fna.gz) and genome annotation (glyma.Wm82.gnm1.ann1.DvBy.gene_models_main.gff3.gz) were downloaded from Soybase (https://soybase.org/data/public/Glycine_max/) and used to conduct SNP annotation *via* the SnpEff software (Cingolani et al., 2012). The SNP variants were extracted from the SnpEff-annotated VCF file using a Perl script. We retained the loss of function mutations described in Torkamaneh et al. (2018) and the variants in 5’UTR, 3’UTR, and upstream of the candidate genes.

The genomes and genome annotations of four and twenty-six accessions were downloaded from Soybase (https://soybase.org/data/public/Glycine_max/) and Bigdata (https://bigd.big.ac.cn/, Project number: PRJCA002030; Liu et al., 2020c), respectively, where the four accessions included W05, PI483463 (wild), Williams 82 (landrace), and ZH13 (cultivar). The genes of Williams 82 were used to create a local BLAST database using the NCBI-BLAST+ (v2.2.31+) software. All the genes in the other 29 genomes were compared with the genes of Williams 82 to search the best-match gene in each of the 29 genomes, which are homologous to the gene of Williams 82. The sequences of genes homologous to candidate genes containing 2 kb upstream were extracted from the 30 genomic sequences by getfasta function in BEDTools (Quinlan, 2014), and these sequences were aligned to obtain SNP variants using the MUSCLE software (Edgar, 2004).

The common 172 soybean accessions between 302 accessions of Zhou et al. (2015b) and the publicly available resources on the USDA GRIN database (http://www.ars-grin.gov/) were used to conduct haplotype analysis using the Haploview v4.1 software (Barrett et al., 2005). The marker genotypes were derived from Zhou et al. (2015b), while the phenotypes of seed oil content were downloaded from the USDA GRIN database. The missing genotypes were imputed using the Beagle v5.1 software (Browning et al., 2018). Multiple comparisons of trait differences among various haplotypes were tested using *LSD.test* function of *agricolae* package in R.

### Candidate genes responded with environments

To identify candidate genes responded to environments, 4,000 most differentially expressed genes in response to temperature which were identified by Weston et al. (2011), and 4,866 differentially expressed genes in response to water deficit which were identified by Rodrigues et al. (2015) were used in this study. The expression matrix of soybean from Weston et al. (2011), including baseline (22 °C), optimum (33.25 °C), 20% inhibition from optimum (40.75 °C), and 30% inhibition from optimum (43.8 °C) with 4 duplications, and the expression matrix of soybean from Rodrigues et al. (2015), including control and drought stress having 6 time periods with 3 duplications, were download from Soybase (https://www.soybase.org/).

The correlation of candidate genes with all the genes in the expression matrices were calculated using the *cor.test* function with Pearson method in R. The significant level was set at 0.01 probability level and correlation coefficient ≥ 0.9.

The promoter sequences, 2 kb upstream of transcript start site of candidate genes, were extracted from Williams 82 V1.1 by getfasta function in BEDTools (Quinlan, 2014). These sequences were used to identify cis-acting regulatory element *via* the plantCARE web site (http://bioinformatics.psb.ugent.be/webtools/plantcare/html/).

### Co-expressional network analysis

The expressional levels of soybean genes under control condition and drought stress in Rodrigues et al. (2015) were analyzed by R package WGCNA v1.70 to construct co-expressional networks. The optimal soft thresholds were calculated by the function “pickSoftThreshold”, and the thresholds were set using r^2^ > 0.8. The TOMType and corType were set as “unsigned” and “bicor”, respectively. minModuleSize was set to 30, and mergeCutHeight was set to 0.3. The top 15 genes with higher kWithin value calculated by *intramodularConnectivity* function of the WGCNA software were defined as hub nodes. The network was visualization using Cytoscape package (Shannon et al., 2003). The KEGG enrichment analysis for the genes in the above co-expressional networks was conducted by KOBAS (http://bioinfo.org/kobas/; Bu et al., 2021).

### The precipitation datasets

The precipitation datasets in Nanjing (2011, 2012, 2014, 2015, and 2016) and Wuhan (2014 and 2015) were downloaded from http://data.sheshiyuanyi.com/.

## Results

### Phenotypic variation of six seed oil-related traits across various environments

All the 286 soybean accessions were measured in five to seven environments for PA, SA, OA, LA, LNA, and OIL in Nanjing and Wuhan, China, and the phenotypes had been described in our previous studies (**Table S1**; Zhou et al., 2016; Liu et al., 2020b; Zuo et al., 2022). The coefficients of variation for these traits and their best linear unbiased prediction (BLUP) values ranged from 5.59 to 17.56 (%), with a mean of 10.24%. These indicate the existence of large genetic variation in association mapping population. In correlation analysis among these traits, some known correlations were observed, i.e., OIL and LNA (negatively), OA and LA/LNA (negatively), and LA and LNA (positively; **Figure S1**).

**Figure 1:**
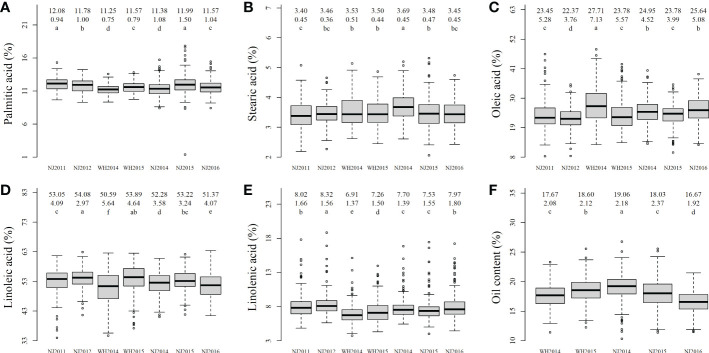
The boxplot of soybean seed oil-related traits in all the environments. **(A–F)** were the phenotype boxplots for palmitic acid, stearic acid, oleic acid, linoleic acid, linolenic acid, and oil content in five to seven environments; The numbers/characters in the first, second, and three rows in the upper of each plot were mean, standard deviation, and the results of multiple comparisons, respectively. a-f in each boxplot marked significant in multiple comparison.

The phenotypic differences among these traits were significant across various environments using multiple comparisons (**Figure 1**). In two-way (environment and genotype) ANOVA, genotypic variation for all traits were highly significant (*P*-values = <1.00 × 10^-300^ ~ 1.85 × 10^-111^); environmental variation was also highly significant (*P*-values = 9.23 × 10^-90^ ~ 6.63 × 10^-28^) (**Table S2**). Year effects had the largest impact on all seed oil-related traits (**Table S3**), location effect had significant impact on all seed oil-related traits excluding SA (**Table S4**), and genotype × location interaction effects were significant for OA and LA (**Table S4**), which are similar to those in Primomo et al. (2002). These results indicated the significant effects of genotype and environment on these traits, although genotype × year interaction effects weren’t significant for all traits (**Table S3**).

### Identification of significant QTNs for six seed oil-related traits using 3VmrMLM

#### QTNs for seed oil-related traits in a single environment

**Figure 2:**
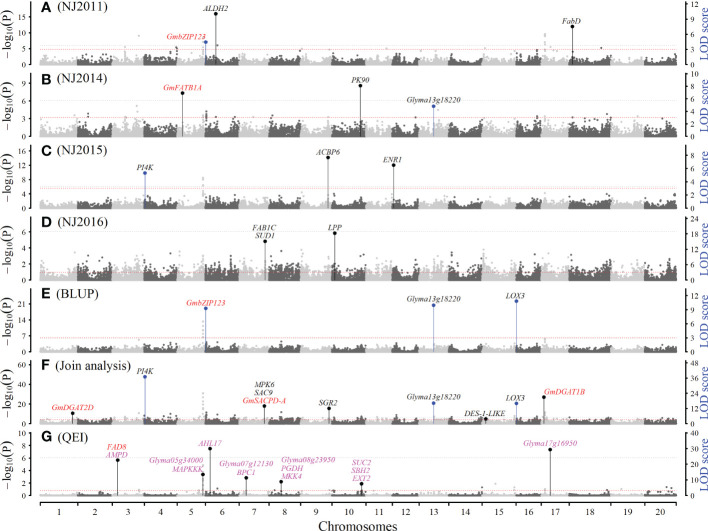
The Manhattan plot for seed linoleic acid content (LA) using software IIIVmrMLM. **(A–D)**: QTNs detected for LA in NJ2011, NJ2013, NJ2015 and NJ2016 using single environment module in software IIIVmrMLM. **(E)**: QTNs detected for LA BLUP values using single environment module in software IIIVmrMLM. **(F)**: QTNs detected for LA across all the seven environments using multi-environment joint analysis module in software IIIVmrMLM. **(G)**: QTN-by-environment interactions (QEIs) detected for LA using multi-environment joint analysis module in software IIIVmrMLM. The black (one) and blue (multiple) lines indicate the number of times that the QTN/QEI was identified. Known genes, candidate genes, and gene-by-environment interactions (GEIs) were marked with red, black, and magenta colors, respectively.

Among 467 QTNs identified for seed oil-related traits, 89, 87, 101, 99, 78, and 59 were found to be associated with PA, SA, OA, LA, LNA, and OIL in a single environment and their BLUP values, respectively (**Figures 2**, **S2**; **Table S5**). These QTNs were located on all the chromosomes (**Table S5**). The LOD scores were 3.10 ~ 46.45 for PA, 3.31 ~ 39.03 for SA, 3.38 ~ 69.99 for OA, 3.74 ~ 81.74 for LA, 3.89 ~ 67.66 for LNA, and 3.21 ~ 51.47 for OIL, and the corresponding average r^2^ were 4.20%, 3.91%, 3.12%, 3.31%, 2.73%, and 3.90%, respectively. Among these QTNs, there were six large QTNs (r^2^ > 10%), such as 13.65% for snp71149-associated SA QTN (**Table S5**).

#### QTNs for six seed oil-related traits in all the environments

Among 200 QTNs identified for seed oil-related traits, 37, 38, 41, 37, 29, and 26 were found to be associated with PA, SA, OA, LA, LNA, and OIL in five to seven environments, respectively (**Figures 2**, **S2**, **S3A**, **S3B**; **Table S6**). These QTNs were located on all the chromosomes (**Table S6**). The LOD scores were 3.42 ~ 79.50 for PA, 4.23 ~ 68.11 for SA, 4.03 ~ 55.41 for OA, 3.56 ~ 187.68 for LA, 13.93 ~ 138.05 for LNA, and 4.33 ~ 46.08 for OIL, and the corresponding average r^2^ were 0.68%, 0.91%, 0.73%, 0.84%, 0.80%, and 0.96%, respectively, such as 4.51% for snp25032-associated LA QTN (**Table S6**).

### QEIs for six seed oil-related traits in all environments using 3VmrMLM

**Figure 3:**
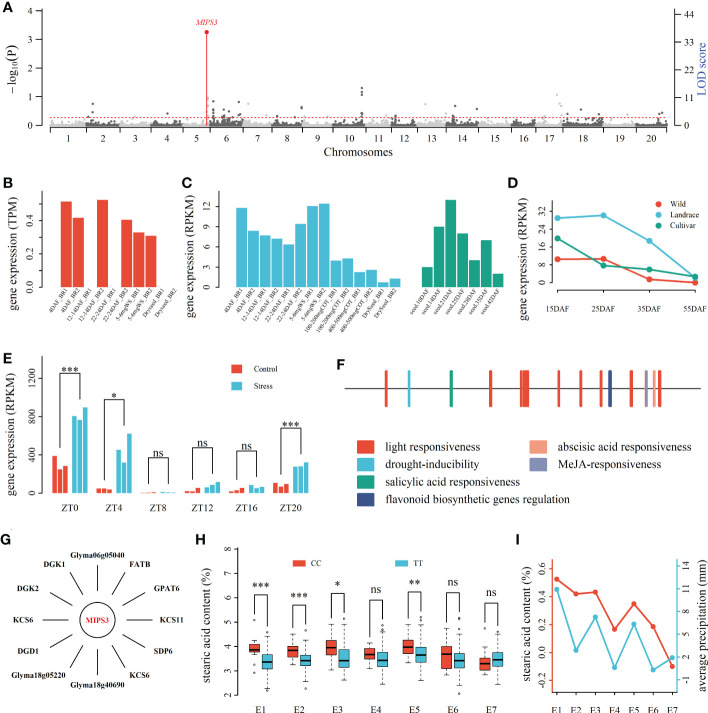
Drought response candidate gene *MIPS3* around QEI for soybean seed oil-related traits. **(A)**: Manhattan plot of QEIs for SA. **(B)**: The expression levels (TPM) of *MIPS3* at five seed development stages (Machado et al., 2020). **(C)**: The expression levels (RPKM) of *MIPS3* in seeds at seven and five seed development stages. The gene expression levels from Jones and Vodkin (2013) and Severin et al. (2010) were marked with blue and green colors, respectively. **(D)**: Differential expression levels (RPKM) of *MIPS3* among wild, landrace, and bred soybeans at four seed development stages (15 ~ 55 DAF; Niu et al., 2020; Liu et al., 2020a), **(E)**: The expression levels (RPKM) of *MIPS3* at six time periods (ZT0 ~ ZT20) under control and drought stress conditions. **(F)**: The cis elements in *MIPS3.* **(G)**: Oil metabolism genes co-expressed with *MIPS3*. **(H)**: Averages for seed stearic acid content across two genotypes of the QEI around *MIPS3* in seven environments. **(I)**: seed stearic acid content between genotypes CC and TT in environments E1~E7 (NJ2011, NJ2012, WH2014, WH2015, NJ2014, NJ2015, and NJ2016) and average precipitation in late August. DAF: day after flowering; ZT: Zeitgeber time. *, **, and ***: significance at the 0.05, 0.01, and 0.001 probability levels, respectively; ns, no significance.

Among 54 QEIs identified for six seed oil-related traits in five to seven environments, 11, 17, 14, 13, and 5 were found to be associated with PA, SA, OA, LA, and OIL, respectively (**Figures 2**, **3A**, **S2**; **Table S7**), and no LNA QEI was identified. These QEIs were located on all the chromosomes (**Table S7**). The LOD scores were 11.82 ~ 119.94 for PA, 4.62 ~ 52.04 for SA, 8.17 ~ 35.50 for OA, 6.57 ~ 29.81 for LA, and 6.43 ~ 37.05 for OIL, and the corresponding average r^2^ were 4.04%, 1.59%, 1.05%, 1.06%, and 2.16%, respectively. Among these QTNs, there was one large QTN (r^2^ > 10%), being 10.05% for snp22240-associated PA QTN. 5 QEIs were common between OA and LA and located on chromosomes 3, 6, 11, 13, and 19 (**Table S7**).

### Mining known and candidate genes around all the QTNs for seed oil-related traits

#### Known genes around all the QTNs

Within 100-kb flanking genomic region for each QTN identified for six seed oil-related traits, there were 8,903 genes. Among these genes, seven had been verified to regulate seed oil-related traits in previous studies (**Table 1**), including *GmSACPD-A* and *GmSACPD-B* (Kachroo et al., 2008), *GmbZIP123* (Song et al., 2013), *GmDGAT2D* (Chen et al., 2016), *GmDGAT1B* (Zhao et al., 2019) and *GmSWEET39*/*GmSWEET10a* (Miao et al., 2020), and *GmFATB1A* (Zhou et al., 2021).

**Table 1:** Seven known genes around QTNs for soybean seed oil- and size-related traits.

Trait	Dataset	Marker	Chr	Pos(bp)	LOD	Add	Dom	r^2^(%)	*P*-value	Sign.	Gene	Homologous gene in Arabidopsis			Reference
												Gene	Symbol	Annotation
PA	IX	snp22231	5	8057446	68.17	0.28	-0.24	1.18	6.74E-69	SIG	*Glyma05g08060* (*GmFATB1A*)	*AT1G08510.1*	FATB	fatty acyl-ACP thioesterases B	Zhou et al. (2021)
PA	VIII	snp22231	5	8057446	28.06	0.27	-0.12	3.19	8.77E-29	SIG	*Glyma05g08060* (*GmFATB1A*)	*AT1G08510.1*	FATB	fatty acyl-ACP thioesterases B	Zhou et al. (2021)
OA	II	snp73103	15	3855027	21.11	1.40	1.09	2.49	7.77E-22	SIG	*Glyma15g05470* (*GmSWEET39*)	*AT5G13170.1*	SAG29	senescence-associated gene 29	Miao et al. (2020)
OA	IX	snp4406	1	48527274	11.18	0.03	-1.62	0.62	6.62E-12	SIG	*Glyma01g36011* (*GmDGAT2D*)	*AT3G51520.1*	DGAT2	diacylglycerol acyltransferase family	Chen et al. (2016)
LA	I	snp25555	6	751417	4.41	-0.58	0.39	1.65	3.91E-05	SUG	*Glyma06g01240* (*GmbZIP123*)	*AT4G34590.1*	bZIP11	G-box binding factor 6	Song et al. (2013)
LA	III	snp22226	5	8031324	6.88	-0.83	0.39	2.31	1.32E-07	SIG	*Glyma05g08060* (*GmFATB1A*)	*AT1G08510.1*	FATB	fatty acyl-ACP thioesterases B	Zhou et al. (2021)
LA	IX	snp4406	1	48527274	8.22	-0.03	1.15	0.49	6.10E-09	SIG	*Glyma01g36011* (*GmDGAT2D*)	*AT3G51520.1*	DGAT2	diacylglycerol acyltransferase family	Chen et al. (2016)
LA	IX	snp35733	7	37646716	13.81	-0.31	-1.79	0.56	1.56E-14	SIG	*Glyma07g32850* (*GmSACPD-A*)	*AT2G43710.1*	SACPD	Plant stearoyl-acyl-carrier-protein desaturase family protein	Kachroo et al. (2008)
LA	IX	snp84224	17	4309255	20.79	0.51	0.03	0.97	1.63E-21	SIG	*Glyma17g06120* (*GmDGAT1B*)	*AT2G19450.1*	DGAT1	membrane bound O-acyl transferase (MBOAT) family protein	Zhao et al. (2019)
LA	VIII	snp25550	6	720481	9.24	-0.11	2.35	3.03	5.72E-10	SIG	*Glyma06g01240* (*GmbZIP123*)	*AT4G34590.1*	bZIP11	G-box binding factor 6	Song et al. (2013)
LNA	III	snp73103	15	3855027	21.14	-0.47	0.70	3.54	7.34E-22	SIG	*Glyma15g05470* (*GmSWEET39*)	*AT5G13170.1*	SAG29	senescence-associated gene 29	Miao et al. (2020)
LNA	VI	snp73103	15	3855027	44.81	-0.79	-0.08	5.66	1.57E-45	SIG	*Glyma15g05470* (*GmSWEET39*)	*AT5G13170.1*	SAG29	senescence-associated gene 29	Miao et al. (2020)
LNA	VIII	snp73103	15	3855027	28.83	-0.50	-0.52	2.58	1.47E-29	SIG	*Glyma15g05470* (*GmSWEET39*)	*AT5G13170.1*	SAG29	senescence-associated gene 29	Miao et al. (2020)
OIL	VI	snp7349	2	14128122	6.29	0.45	-0.17	3.09	5.17E-07	SIG	*Glyma02g15600* (*GmSACPD-B*)	*AT2G43710.1*	SACPD	Plant stearoyl-acyl-carrier-protein desaturase family protein	Kachroo et al. (2008)
OIL	VI	snp73103	15	3855027	11.48	0.61	-0.19	1.61	3.34E-12	SIG	*Glyma15g05470* (*GmSWEET39*)	*AT5G13170.1*	SAG29	senescence-associated gene 29	Miao et al. (2020)

PA, palmitic acid; OA, oleic acid; LA, linoleic acid; LNA, linolenic acid; OIL, oil content; Dataset I ~ VIII, the detection of main-effect QTNs for the phenotype of seed oil-related traits in NJ2011, NJ2012, NJ2014, NJ2015, NJ2016, WH2014, WH2015, and BLUP using Single-Env method of 3VmrMLM; Dataset IX, the detection of main-effect QTNs for the phenotype of seed oil-related traits across all environment using Multi-Env method of 3VmrMLM; Chr, chromosome; Pos, position; Add, additive; Dom, dominance; SIG, significant; SUG, suggestion.

#### Candidate oil metabolism genes

The above 8,896 genes around the above loci were compared with 1,123 oil-related genes in Zhang et al. (2016), and 195 genes were found to be associated with oil biosynthesis. The remains were used to conduct KEGG pathway analysis, and 57 genes were found to be associated with oil biosynthesis. Thus, 252 genes were found to be associated with oil biosynthesis.

**Figure 4:**
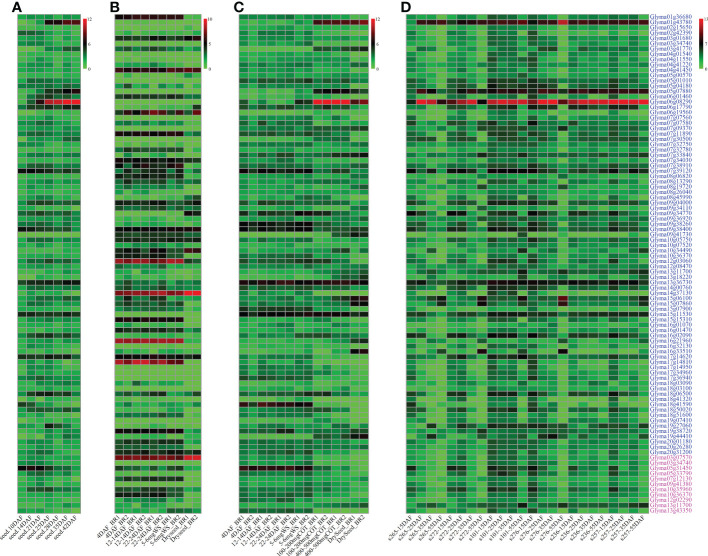
The expressional levels of 83 genes around QTNs and 11 genes around QEIs for soybean seed oil-related traits. Green and red colors represent the lower and higher expression levels, respectively. Gene expressional levels at various seed developmental stages are derived from Severin et al. (2010) **(A**, RPKM**)**, Machado et al. (2020) **(B**, TPM**)**, Jones and Vodkin (2013) **(C**, RPKM**)**, and Niu et al. (2020) or Liu et al. (2020a) **(D**, RPKM**)**, respectively. The genes around QTNs and QEIs were marked with blue and magenta color, respectively. DAF: day after flowering. s265 and s272: wild soybeans; s101 and s276: landraces; s236 and s257: bred soybeans.

The gene expressional datasets from Severin et al. (2010); Jones and Vodkin (2013), and Machado et al. (2020) were used to conduct high expression analysis (**Figure 4**). As a result, 159 genes were found to have high expressional levels at seed oil accumulation stages. The gene expressional datasets among two wild, two landrace, and two bred soybeans from Niu et al. (2020) and Liu et al. (2020a) were used to mine differentially expressed genes (DEGs). As a result, 83 genes were differentially expressed among wild, landrace, and bred soybeans (*P* ≤ 0.001; **Figure 4**). Based on expressional levels of genes in 14 soybean tissues in Severin et al. (2010), only 3 DEGs were found to express in seed rather than other tissues, being *Glyma01g43780*, *Glyma05g07880*, and *Glyma06g08290*, in which *Glyma01g43780* is homologous to *AT4G10020* (*AtHSD5*) coding hydroxysteroid dehydrogenase 5, and *Glyma05g07880* (*GmOLE2*) and *Glyma06g08290* (*GmOLE4*) code oleosin family protein. These three genes specially expressed in seed may play an important role in the accumulation of soybean seed oil content, and the other 80 genes also may regulate seed oil-related traits.

#### SNP variants

The SNP genotypes of 302 soybean accessions in Zhou et al. (2015b) were used to identify SNP variants for 7 known and 83 candidate genes. As a result, all the 90 genes had SNP variants in upstream, UTR, and CDS of these genes, in which all the 90 genes have SNP variants in upstream, 85 gene have SNP variants in UTR, and 65 genes have SNP variants in CDS, such as fifteen SNPs in the promoter, two SNPs in 5’UTR, and one SNP in 3’UTR for known gene *GmDGAT2D* (**Figure S3C**). It should be noted that *Glyma06g01460* (*KCS4*) and *Glyma07g32780* (*SAC9*) had stop gained variants, *GmDGAT2B* had stop lost variant, and *Glyma16g33510* (*AGAL2*) had start lost variant.

Thirty soybean genome sequences were used to further verify these SNP variants. As a result, 84 genes were found to have SNP variants, in which all the 84 genes have SNP variants in upstream, 64 genes have SNP variants in UTR, and 44 genes have SNP variants in CDS. It should be noted that *GmDGAT2B* had stop lost variant.

#### Haplotype analysis

The SNP variants in 302 soybean accessions and 30 soybean genomes, along with seed oil content for 172 soybean accessions, were used to conduct haplotypic analysis. As a result, 7 known (**Table 1**) and 76 candidate (**Table S9**) genes, excluding *Glyma05g00570* (*DGK1*), were found by multiple comparison to have significant differences across various haplotypes (**Tables 2**, **S8**; **Figure S4**), such as known gene *GmDGAT2D* (**Figure S3D**).

**Table 2:** The ANOVA for soybean seed oil content among different haplotypes of genes around QTNs.

Gene	*F*-value	*P*-value	Gene	*F*-value	*P*-value	Gene	*F*-value	*P*-value	Gene	*F*-value	*P*-value
***GmDGAT2D***	26.90	2.36E-20	*Glyma07g09370*	11.20	1.11E-10	*Glyma09g41730*	15.85	4.08E-12	*Glyma16g33510*	5.18	6.26E-04
*Glyma01g36680*	13.32	8.44E-08	*Glyma07g11890*	61.11	5.33E-33	*Glyma10g05750*	24.17	2.79E-17	***GmDGAT1B***	8.34	2.40E-08
*Glyma01g43780*	22.77	4.03E-12	*Glyma07g30500*	24.09	1.76E-12	*Glyma10g07520*	10.32	3.01E-06	*Glyma17g14620*	16.73	9.14E-18
***GmSACPD-B***	55.34	1.17E-30	*Glyma07g32750*	90.49	3.36E-39	*Glyma10g34490*	67.56	1.87E-27	*Glyma17g14810*	20.55	3.19E-13
*Glyma02g15650*	5.94	1.59E-02	*Glyma07g32780*	18.78	4.06E-14	*Glyma10g36370*	41.24	3.59E-22	*Glyma17g14950*	8.99	2.33E-06
*Glyma02g42390*	25.89	6.05E-24	***GmSACPD-A***	37.50	1.16E-17	*Glyma12g03060*	5.58	1.18E-03	*Glyma17g34960*	20.58	3.73E-11
*Glyma03g34740*	7.41	3.32E-06	*Glyma07g33840*	130.71	1.80E-22	*Glyma13g11700*	21.87	4.37E-16	*Glyma17g36940*	16.44	3.08E-16
*Glyma03g41770*	44.48	2.09E-20	*Glyma07g34030*	49.10	5.35E-26	*Glyma13g18220*	15.72	8.06E-11	*Glyma18g03090*	8.57	4.50E-07
*Glyma04g01540*	18.63	2.87E-10	*Glyma07g38910*	60.45	2.42E-36	*Glyma13g36730*	34.16	1.19E-23	*Glyma18g03100*	11.15	7.46E-08
*Glyma04g11550*	18.59	3.31E-12	*Glyma07g39120*	56.57	4.05E-24	*Glyma14g00760*	27.02	9.92E-17	*Glyma18g06500*	17.77	1.03E-13
*Glyma04g41220*	18.57	2.80E-10	*Glyma08g06820*	40.13	1.40E-24	*Glyma14g37130*	33.57	7.57E-20	*Glyma18g41320*	52.78	3.54E-27
*Glyma04g41450*	24.49	3.37E-15	*Glyma08g13290*	6.30	6.99E-06	***GmSWEET39***	128.75	8.65E-33	*Glyma18g41590*	303.56	9.01E-38
*Glyma05g04180*	13.96	1.77E-09	*Glyma08g19720*	31.83	5.28E-16	*Glyma15g07860*	40.69	9.63E-25	*Glyma18g50020*	5.28	2.09E-04
*Glyma05g07880*	71.46	1.70E-28	*Glyma08g26040*	34.05	3.06E-08	*Glyma15g07900*	50.03	2.46E-26	*Glyma18g51600*	27.10	8.78E-11
***GmFATB1A***	10.55	2.36E-06	*Glyma08g45990*	21.20	1.54E-11	*Glyma15g11530*	28.45	1.58E-19	*Glyma19g07410*	18.69	3.28E-12
***GmbZIP123***	6.27	2.85E-05	*Glyma09g04000*	54.68	5.51E-24	*Glyma15g15310*	37.95	6.90E-29	*Glyma19g27060*	11.21	2.79E-05
*Glyma06g01460*	14.46	2.94E-08	*Glyma09g34110*	19.34	1.10E-12	*Glyma16g01070*	23.98	1.08E-12	*Glyma19g44410*	24.37	5.86E-13
*Glyma06g08290*	28.92	6.64E-18	*Glyma09g34770*	19.83	2.55E-17	*Glyma16g01470*	56.65	5.37E-19	*Glyma20g01180*	27.83	4.40E-23
*Glyma06g19560*	14.18	2.36E-04	*Glyma09g36920*	29.89	4.43E-22	*Glyma16g02090*	46.72	4.59E-25	*Glyma20g26280*	32.60	1.14E-23
*Glyma07g07560*	17.29	1.24E-14	*Glyma09g38260*	26.82	1.05E-21	*Glyma16g21960*	26.46	1.57E-10	*Glyma20g31200*	34.41	8.13E-23
*Glyma07g07580*	20.81	1.98E-15	*Glyma09g38400*	21.84	6.74E-19	*Glyma16g32130*	21.27	1.64E-11			

The genes with bold type were previously reported to be truly associated with oil metabolism.

### Mining known and candidate genes around QEIs for seed oil-related traits

#### Known gene around the QEIs

Within 100-kb flanking genomic region for each QEI identified for seed oil-related traits, there were 863 genes in total. Among these genes, *GmFATB2B* had been verified to regulate seed oil-related traits, and homologous gene in sorghum had been reported to play important roles in drought-induced wax biosynthesis (**Table 3**; Sanjari et al., 2021; Zhou et al., 2021).

**Table 3:** Candidate gene-by-environment interactions for soybean seed oil-related traits.

Trait	Marker	Chr	Pos (bp)	LOD	r^2^(%)	*P*-value	Sign.	Gene	Homologous gene in Arabidopsis			Oil metabolism	Response to environment stress in	Gene expression trend		Reference
									Gene	Symbol	Annotation		previous studies	Heat stress	Drought stress	
SA	snp24808	5	36671535	36.95	2.71	4.46E-30	SIG	*Glyma05g31450*	*AT5G10170.1*	MIPS3	myo-inositol-1-phosphate synthase 3		heat stress		Up 239.36%	Khurana et al. (2017)
SA	snp24808	5	36671535	36.95	2.71	4.46E-30	SIG	*Glyma05g31630*	*AT4G39660.1*	AGT2	Alanine:glyoxylate aminotransferase 2			Up 125.02%		Weston et al. (2011)
SA	snp28582	6	20835485	52.04	3.88	1.97E-44	SIG	*Glyma06g23560* (*GmFATB2B*)	*AT1G08510.1*	FATB	fatty acyl-ACP thioesterases B	Fatty acid biosynthesis	drought stress			Zhou et al. (2021)
SA	snp26121	6	4685918	30.48	2.34	5.08E-24	SIG	*Glyma06g06550*	*AT5G25110.1*	CIPK25	CBL-interacting protein kinase 25				Up 234.54%	Rodrigues et al. (2015)
SA	snp32821	7	8047531	18.27	1.37	6.67E-13	SIG	*Glyma07g09570*							Up 252.16%	Rodrigues et al. (2015)
SA	snp35491	7	35825129	16.83	1.19	1.24E-11	SIG	*Glyma07g30950*	*AT1G61340.1*	FBS1	F-box family protein			Up 391.94%		Weston et al. (2011)
SA	snp67206	13	43130940	11.27	0.77	6.43E-07	SIG	*Glyma13g43350*	*AT1G79840.1*	GL2	HD-ZIP IV family of homeobox-leucine zipper protein with lipid-binding START domain	Homologous gene affects seed oil content in *Arabidopsis*	salt stress			Shen et al. (2006); Belamkar et al. (2014)
OA	snp11943	3	8113468	21.07	1.32	2.11E-15	SIG	*Glyma03g07570*	*AT5G05580.1*	FAD8	fatty acid desaturase 8	Fatty acid biosynthesis	temperature			Gibson et al. (1994)
OA	snp25014	5	38379318	10.52	0.64	2.62E-06	SUG	*Glyma05g33790* (*GmPEAMT*)	*AT3G18000.1*	PEAMT	S-adenosyl-L-methionine-dependent methyltransferases superfamily protein	Glycerophospholipid metabolism	plant stress			Ji et al. (2021)
OA	snp25644	6	1490151	13.45	0.84	1.00E-08	SIG	*Glyma06g02380*	*AT1G60420.1*		DC1 domain-containing protein				Up 585.28%	Rodrigues et al. (2015)
OA	snp36510	7	43205262	13.41	0.85	1.09E-08	SIG	*Glyma07g38390*	*AT4G15530.4*	PPDK	pyruvate orthophosphate dikinase				Up 174.98%	Rodrigues et al. (2015)
OA	snp47463	9	46005575	13.88	0.83	4.29E-09	SIG	*Glyma09g41380*	*AT1G62640.2*	KAS III	3-ketoacyl-acyl carrier protein synthase III	Fatty acid biosynthesis	temperature			Takami et al. (2010)
OIL	snp52940	11	2571951	6.47	0.79	2.28E-04	SUG	*Glyma11g03690*	*AT5G47450.1*	DELTA-TIP3	tonoplast intrinsic protein 2;3			Up 190.29%	Down 30.17%	Weston et al. (2011)

SA, stearic acid; OA, oleic acid; Chr, chromosome; Pos, position; Add, additive; Dom, dominance; SIG, significant; SUG, suggestion.

#### Candidate oil metabolism genes

The above 862 genes were compared with 1,123 oil-related genes in Zhang et al. (2016), and 24 genes were found to be associated with oil biosynthesis. The remains were used to conduct KEGG pathway analysis, and 5 genes were found to be associated with oil biosynthesis. Thus, 29 genes were found to be associated with oil biosynthesis.

#### Candidate environmental interaction genes

The mentioned-above 863 genes around QEIs were compared with DEGs between various environments in Weston et al. (2011) and Rodrigues et al. (2015). As a result, 107 genes were common, in which 40 were DEGs between different temperature conditions (Weston et al., 2011), and 69, including *MIPS3*, were DEGs between control and water deficit (Rodrigues et al., 2015), such as there were significantly different expression level for *MIPS3* between control and drought stress conditions in ZT0 (*P* ≤ 0.001), ZT4 (*P* ≤ 0.05), and ZT20 (*P* ≤ 0.001) using *t*-test (**Figure 3E**).

The plantCARE website (http://bioinformatics.psb.ugent.be/webtools/plantcare/html/) was used to identify cis-acting regulatory elements of the 107 DEGs in response to environment. As a result, 34 genes were found to have cis-elements in response to temperature, including cis-acting elements involved in low-temperature and abscisic acid responsiveness, and 57 genes were found to have cis-elements in response to drought, including cis-acting elements involved in abscisic acid responsiveness or MYB binding site involved in drought-inducibility. For example, the promoter of *MIPS3* included cis-acting element MYB binding site involved in drought-inducibility and abscisic acid responsiveness (**Figure 3F**).

The above-mentioned 34 DEGs under various temperatures were found to be co-expressed with 7,718 genes under 33.25 °C and 5,643 genes under 43.8 °C, in which there are 3,618 different co-expression genes. More importantly, 32 temperature-related DEGs were found to be co-expressed with 89 oil-metabolism-related genes. The above-mentioned 57 drought-stress-related DEGs were found to be co-expressed with 1,961 genes in control and 4,524 genes in drought stress, in which there are 3,576 different co-expression genes. More importantly, 32 drought-stress-related DEGs were found to be co-expressed with 65 oil-metabolism-related genes. For example, *MIPS3* was found to be co-expressed with 12 oil-metabolism-related genes (**Figure 3G**). It should be noted that *Glyma11g03690* was a common DEG in response to temperature and drought, and *MIPS3* was oil-metabolism-related DEG in response to drought (**Figure 3**; **Tables 3**, **S10**). These candidate genes may respond to various environment conditions and regulate oil-metabolism-related genes, and indirectly regulate seed oil-related traits.

Thus, a total of 75 candidate genes around QEIs may interact with various environment conditions and participate in oil biosynthesis, including one known oil-biosynthesis-related *GmFATB2B*, 12 highly and differentially expressed genes related to oil biosynthesis, and 32 temperature-related and 32 drought-stress-related DEGs, which are co-expressed with oil-metabolism-related genes (**Tables 3**, **S10**).

#### SNP variants

The SNP genotypes of 302 soybean in Zhou et al. (2015b) were used to identify SNP variants for the above 75 candidate genes around QEIs. As a result, all the 75 genes had SNP variants in upstream, UTR, and CDS of genes, in which all the 75 genes have SNP variants in upstream, 68 gene have SNP variants in UTR, and 60 genes have SNP variants in CDS, such as five SNPs in the promoter, one SNP in 5’UTR, and one SNP in 3’UTR for a known gene *GmFATB2B*.

Thirty soybean genome sequences were used to further verify these SNP variants. As a result, 70 genes were found to have SNP variants, in which 68 genes have SNP variants in upstream, 54 genes have SNP variants in UTR, and 38 genes have SNP variants in CDS.

#### Haplotype analysis

The SNP variants in 302 soybean accessions and 30 soybean genomes, along with seed oil content for 172 soybean accessions, were used to conduct haplotypic analysis. As a result, 67 GEIs, including the known gene *GmFATB2B2B*, 11 oil-metabolism genes, 29 candidate temperature response genes, 28 candidate drought response genes, were found by multiple comparisons to have significant differences across various haplotypes (**Table 4**).

**Table 4:** The ANOVA for soybean seed oil content among different haplotypes of genes around QEIs.

Gene	*F*-value	*P*-value	Gene	*F*-value	*P*-value	Gene	*F*-value	*P*-value	Gene	*F*-value	*P*-value
*Glyma03g05620*	5.50	1.32E-03	*Glyma06g06440*	74.42	7.20E-30	*Glyma08g23860*	52.42	6.43E-23	*Glyma12g02230*	47.07	4.80E-28
*Glyma03g05630*	4.94	8.44E-03	*Glyma06g06550*	42.68	1.19E-19	*Glyma08g23900*	40.67	1.26E-22	*Glyma12g02270*	13.28	9.79E-08
*Glyma03g07460*	12.53	5.48E-10	*Glyma06g09810*	22.71	3.15E-12	*Glyma08g23950*	54.33	8.84E-28	*Glyma12g02290*	3.44	3.45E-02
*Glyma03g07570*	14.30	5.46E-11	***GmFATB2B***	36.86	8.48E-24	*Glyma09g37450*	39.68	2.00E-25	*Glyma12g02330*	37.49	3.35E-26
*Glyma03g34740*	7.37	3.58E-06	*Glyma07g09570*	10.19	4.25E-06	*Glyma09g41270*	17.58	4.43E-17	*Glyma13g11700*	22.21	2.58E-16
*Glyma03g34770*	88.99	2.71E-33	*Glyma07g09590*	10.05	6.28E-09	*Glyma09g41380*	18.30	6.81E-10	*Glyma13g43350*	14.39	5.27E-10
*Glyma03g34830*	6.81	4.92E-05	*Glyma07g09640*	5.82	6.89E-05	*Glyma10g35381*	48.37	3.71E-17	*Glyma13g43570*	14.29	1.45E-12
*Glyma03g34880*	18.26	4.97E-12	*Glyma07g12130*	7.52	1.74E-05	*Glyma10g35430*	85.13	1.41E-16	*Glyma17g02620*	106.96	3.44E-28
*Glyma05g31450*	18.00	5.11E-14	*Glyma07g12150*	45.43	1.58E-20	*Glyma10g35950*	19.11	4.03E-08	*Glyma17g02640*	8.99	1.85E-05
*Glyma05g31630*	19.74	6.37E-13	*Glyma07g30800*	19.80	9.09E-13	*Glyma10g35960*	58.42	1.51E-19	*Glyma17g16950*	87.53	4.55E-26
*Glyma05g31640*	13.50	2.36E-09	*Glyma07g30950*	39.03	1.39E-18	*Glyma10g36180*	8.77	2.45E-04	*Glyma18g09000*	7.12	1.68E-04
*Glyma05g31670*	9.77	5.56E-07	*Glyma07g38350*	17.67	1.23E-07	*Glyma10g36200*	30.39	6.43E-21	*Glyma18g50340*	12.22	9.19E-10
*GmPEAMT*	20.42	1.41E-13	*Glyma07g38390*	34.09	6.23E-13	*Glyma10g36230*	8.32	4.67E-06	*Glyma18g50390*	17.58	2.16E-16
*Glyma05g33910*	24.72	1.37E-19	*Glyma07g38460*	29.20	2.00E-17	*Glyma10g36370*	41.01	3.85E-22	*Glyma19g10120*	19.03	2.18E-14
*Glyma05g34000*	31.19	1.68E-15	*Glyma07g38510*	94.78	1.01E-27	*Glyma11g03690*	44.95	7.93E-27	*Glyma19g29610*	25.06	1.31E-21
*Glyma06g02330*	81.29	7.98E-25	*Glyma08g08520*	37.01	4.30E-18	*Glyma11g03700*	108.56	1.83E-19	*Glyma20g24320*	5.26	6.28E-03
*Glyma06g02380*	74.99	1.38E-23	*Glyma08g08610*	31.54	2.05E-21	*Glyma12g02140*	32.67	1.01E-27			

The genes with bold type were previously reported to be truly associated with oil metabolism.

## Discussion

Seed oil content and its composition in soybean significantly vary across various genotypes and environments (**Tables S2**-**S4**). With global temperature increase steadily in recent decades, high temperature conditions, accompanied either by drought or by humidity in different areas, caused damages and losses on crop yield and quality (Zhang et al., 2019). Although some genes had been reported to regulate seed oil-related traits under these conditions (Kachroo et al., 2008; Song et al., 2013; Zhao et al., 2019; Miao et al., 2020; Zhou et al., 2021), few QEIs and GEIs have been reported owing to the limitation of methodologies in QEI detection of genome-wide association studies. Recently, our group established a new comprehensive GWAS method, 3VmrMLM, for detecting QTNs, QEIs, and QQIs under controlling all the possible polygenic backgrounds (Li et al., 2022a; Li et al., 2022b). Therefore, this study focused on the identification of QTNs, QEIs, and their known and candidate genes in various environments. As a result, 598 QTNs and 54 QEIs for seed oil content and its composition were identified in five to seven environments (**Tables S5**-**S7**; **Figures 2**, **S2**). Among these QTNs, 118 were new, and 480 had been reported in Soybase, in which 63 and 467 were the same with QTNs and QTLs, respectively, that were previously reported in Soybase (https://www.soybase.org/); 7 known and 76 new candidate genes were mined (**Tables 1**, **S9**). Around these QEIs, one oil metabolism gene *GmFATB2B* previously reported in soybean and five oil metabolism genes responsive to environment in other species (wheat and *Arabidopsis*) were identified, and 61 new candidate genes were mined (**Tables 3**, **S10**). Haplotype analysis showed that there were significant differences in seed oil content among the haplotypes of these genes, indicating the associations of these genes with seed oil content. These known and candidate genes provide gene sources for soybean breeding and molecular biology research.

### Candidate gene-by-environment interactions may directly/indirectly regulate soybean seed oil-related traits

In previous studies, some oil-related genes in response to environment had been mined, such as *GmFAD3A* (Wang et al., 2019; Li et al., 2022b), *SACPD* (Kachroo et al., 2008), and *GmPLDα1* (Zhang et al., 2019). In this study, one known (*GmFATB2B*) and 11 candidate oil-metabolism genes around QEIs were identified (**Tables 3**, **S10**; Zhou et al., 2021). Among these candidate genes, *GmPEAMT1*, encoding a phosphoethanolamine methyltransferase (PEAMT), which plays a role in lipid synthesis, may be involved in plant stress response (Ji et al., 2021). *MIPS3* is homologous to wheat *TaMIPS2*, which had been verified to be in response to heat stress (Khurana et al., 2017). *GmFAD8* is homologous to *Arabidopsis* gene *AtFAD8*, which had been verified to be in response to temperature (Gibson et al., 1994); *GL2* is ortholog of *Arabidopsis* gene *GLABRA2* that affects seed oil content in *Arabidopsis* (Shen et al., 2006), in which *GL2* had nearly zero expression level under control condition and were upregulated under salt stress (Belamkar et al., 2014); *KAS III*, in response to low temperature in *Arabidopsis* (Takami et al., 2010), catalyzes the first decarboxy condensation step in *de novo* fatty biosynthesis. These soybean genes may regulate oil metabolism and response to various environment conditions.

Main environment factors that affect seed oil content and its fatty acid compositions in soybean are drought and temperature/heat (Bellaloui et al., 2013). Ding et al. (2020) concluded that heat stress can cause protein misfolding and reactive oxygen species (ROS) accumulation in plant cells, which have negative effects on plants. In this study, 29 candidate genes were identified by differential expression, cis-acting element, and co-expression with oil-metabolism genes to be in response to temperature. Among these candidate genes, 12 and 17 were found to be up (2.73%~391.94%) and down (5.27%~72.76%) regulation under heat stress, respectively (**Tables 3** and S10). In particular, three candidate genes were found to be upregulated by more than 100% under heat stress (**Table 3**). Meanwhile, drought stress can increase production of ROS in Kaur and Asthir (2017), while in Osakabe et al. (2014), plant responses to water stress are controlled by complex regulatory events mediated by abscisic acid (ABA) signaling, ion transport, and activities of transcription factors (TFs) involved in the regulation of stomatal responses. In this study, 28 candidate genes were identified by differential expression, cis-acting element, and co-expression with oil-metabolism genes to be in response to drought. Among these candidate genes, 10 and 18 were found to be up (12.36%~585.28%) and down (1.55%~30.17%) regulation under drought, respectively (**Tables 3**, **S10**). In particular, five candidate genes were found to be upregulated by more than 100% under drought stress (**Table 3**). For example, expression level of *MIPS3* was significantly different between control and water stress (**Figure 3E**) and up-regulated by 239.36% under water stress to control condition, and *MIPS3* was homologous with wheat gene *TaMIPS2* responded to heat stress (Khurana et al., 2017). In addition, candidate heat stress gene *Glyma05g31670* was homologous to *AtOSA1*, which was response to oxidative stress (Jasinski et al., 2008); *Glyma07g12150* was homologous to *Malus BPC1*, which regulated the expression of *CCD7* involved in stress response (Yue et al., 2015); *Glyma07g30950* was homologous to *Arabidopsis* F-box gene *FBS1* (*At1g61340*) induced by drought, heat, and cold stresses (Gonzalez et al., 2017); *Glyma10g35430* was homologous to *AtNADK-1* induced by oxidative stress (Berrin et al., 2005). Candidate drought stress gene *Glyma05g33910* was homologous to *At1g73660*, which is responded to salt stress (Gao and Xiang, 2008).

To understand whether there are differences between the networks under control and environmental stress conditions, and which candidate genes in the differential pathways are in response to the environments, the expressional levels of 4,866 DEGs between control and drought stress in Rodrigues et al. (2015) were used to construct co-expression network using R package WGCNA v1.70 (Langfelder and Horvath, 2008). As a result, three co-expression modules and none co-expression grey module were identified under drought stress. The genes in each co-expression module were used to conduct KEGG pathway enrichment analysis using KOBAS (http://bioinfo.org/kobas/; Bu et al., 2021). Results showed that three co-expression modules, brown (181 genes), blue (521 genes), and turquoise (1,408 genes), were enriched in 1, 2, and 9 KEGG pathways, respectively. Among these three kinds of pathways, 1, 2, and 3 were associated with drought response, including plant hormone signal transduction, brassinosteroid biosynthesis, base excision repair, nucleotide excision repair, and mismatch repair (corrected *P*-value < 0.05; **Table S11**). Plant hormones, including ABA and brassinosteroid, play important roles in the regulation of drought stress (Ma et al., 2019; Gupta et al., 2020). Proper regulation of DNA repair is required for drought tolerance (Shim et al., 2018). DNA repair mechanisms include base excision repair, nucleotide excision repair, and DNA mismatch repair. In this study, almost all (27 in 28) candidate genes response to drought were in the brown, blue, and turquoise modules, in which there are respectively 4, 10, and 13 candidate genes.

Nine co-expression modules and none co-expression grey module were identified under control condition. KEGG pathways enrichment analysis showed that magenta (53), pink (62), black (90), red (128), green (176), yellow (343), brown (488), blue (825), and turquoise (1,475) modules were enriched in 1, 2, 0, 1, 4, 2, 0, 1, and 3 KEGG pathways, respectively. Among these pathways, plant hormone signal transduction, and base excision repair were enriched in blue and yellow modules, respectively (corrected *P*-value < 0.05; **Table S12**). In this study, a total of 9 candidate genes were in the two co-expression modules, 8 and 1 genes were respectively in blue and yellow modules.

Four KEGG pathways, including brassinosteroid biosynthesis, starch and sucrose metabolisms, mismatch repair, and nucleotide excision repair, were enriched in co-expression modules under drought stress rather than control condition. Especially, brassinosteroid biosynthesis, mismatch repair, and nucleotide excision repair were associated with drought response. Two KEGG pathways, plant hormone signal transduction and base excision repair, were enriched in co-expression module under both drought stress and control condition. However, their genes in the two pathways were different and involved in different biotechnology processes (**Figure S5**), and the linked genes were also different. For example, 16 and 20 genes enriched in plant hormone signal transduction under drought stress and control condition, respectively, but only 5 genes were common. Furthermore, these genes constructed different co-expression network. Most genes (433 in 521) of co-expression network under drought stress were different from the genes under control condition. For example, 7 candidate genes in this study were co-expressed with the genes involved in plant hormone signal transduction under drought stress rather than under control condition. Thus, we speculated that candidate genes response to drought may be accompanied by two ways. First, candidate genes may respond to drought *via* three pathways: brassinosteroid biosynthesis, mismatch repair, and nucleotide excision repair, which are involved in drought response and enriched in co-expression modules under drought stress rather under control condition. Second, candidate genes may interact synergistically with various genes in the co-expression network to respond drought.

**Figure 5:**
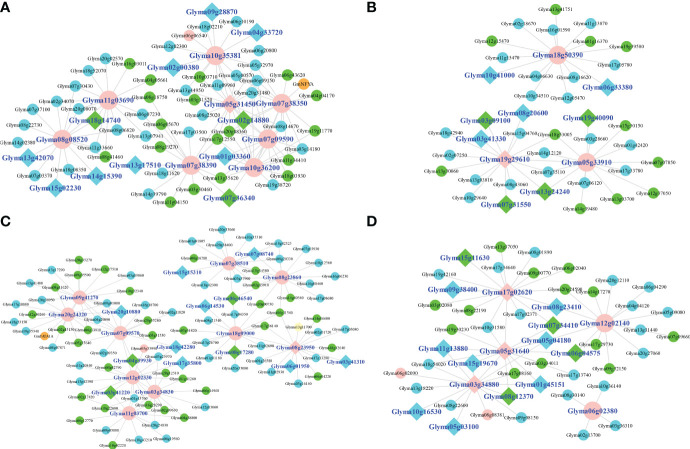
The subnetwork of candidate genes under drought stress. **(A–D)**: The blue module, brown module, turquoise, and yellow module, respectively. The known oil genes, oil metabolism genes, drought response genes, genes involved in both oil metabolism and drought response, and candidate genes were marked with orange, blue, green, yellow, and plink colors, respectively. The hub genes of each module were marked diamond shape.

The oil metabolism genes reported in Zhang et al. (2016), the genes previously confirmed to regulate seed oil-related traits, the genes of KEGG pathways involved in oil metabolism and drought response, and 28 candidate drought genes in this study were used to construct co-expression network. As a result, seven and five co-expression modules were identified under control and drought stress conditions, respectively, and all modules could be enriched to the pathways involved in oil metabolism and drought stress responses. 105 and 75 hub genes were identified under control condition and drought stress, respectively, and most hub genes were different, although 21 hub genes were common. Moreover, no candidate gene was hub gene under control condition (**Figure S6**), while six candidate genes were hub genes under drought stress (**Figure 5**), indicating the hub role of candidate genes in this study in drought stress responses. The top 5 genes tightly linked to each candidate gene in each module were analyzed. The results showed that the tightly linked genes under control condition were most significantly enriched in metabolic pathways, while the tightly linked genes under drought stress were most significantly enriched in plant hormone signal transduction. These results indicate that these candidate genes may participate in some metabolism processes, including oil metabolism, but they play an important role in response to drought stress when plants were exposed to drought stress.

These candidate genes were co-expressed with oil metabolism genes under environmental stress rather than under control condition, e.g., *MIPS3* was co-expressed with 12 oil metabolism genes (|r| > 0.9, *P* < 2.16×10^-7^), including *GPAT6* (r = 0.9194, *P* = 6.93×10^-8^) and *FATB* (r = 0.906, *P* = 2.16×10^-7^) under drought stress rather than under control condition (**Figure 3G**). Thus, we speculated that these candidate genes may act as a bridge between environmental response and regulation of genes for seed oil-related traits. For example, *MIPS3* may respond environment stress *via* the pathway of plant hormone signal transduction, and play an important role in seed stearic acid content together with oil metabolism genes *GPAT6* and *FATB.* For *MIPS3* around one QEI, two-way (genotypes and environments) ANOVA showed the significance for genotype by environment interaction for stearic acid content (*P* < 0.01), and average seed stearic acid content with genotype CC were significantly higher than that with genotype TT in NJ2011, NJ2012, WH2014, and NJ2014 (**Figure 3H**). We also found that the difference of average seed stearic acid content between genotypes CC and TT was significantly correlated with the average precipitation in late August (*P* < 0.05; **Figure 3I**). In other words, the higher precipitation, the larger SA difference. Thus, we speculated that *MIPS3* may respond to drought stress and co-expressed with oil metabolism genes, and then regulate the genes for soybean seed oil-related traits. The above results further indicated the reliability of candidate GEIs identified in this study.

### Domestication and improvement analyses of oil-metabolism-related candidate genes, confirmed by haplotype analysis, reveal some available genes in future soybean breeding

Among these known and candidate oil-metabolism-related genes around QTNs, 7 known and 76 candidate genes had SNP variants in upstream, UTR, and CDS. These variants were used to constitute haplotypes. Haplotype analysis indicated that haplotypes of each gene had significant different seed oil content using multiple comparisons (**Figures S3D**, **S4**). In domestication and improvement analysis *via* 302 soybean accessions, frequencies of elite haplotypes for all the 83 candidate genes increased from wild to landrace soybeans, and frequencies of elite haplotypes for 71 candidate genes increased from landrace to bred soybeans, showing the evidence of selection sweep for all the 83 genes (**Tables 2**, **S8**; **Figures S3E**, **S4**). Furthermore, frequencies of elite haplotypes for 50 candidate genes were more than 90% in bred soybeans, while frequencies of elite haplotypes for 8 candidate genes were less than 50% in bred soybeans and may be available in future soybean breeding, such as *SAC9* (20.00%) (**Tables S8**, **S13**).

### Pleiotropic QTNs reveal the correlation of soybean seed oil-related traits

Among QTNs for seed oil-related traits in this study, 50 QTNs were found to be associated with at least two traits (**Figure S7**), including 3 QTNs for three traits, and 47 QTNs for two traits. Interesting, there were 41 common QTNs between OA and LA, explaining their very significant correlation (-0.922, *P* < 0.001; **Figures S1**, **S7**). For example, around a common QTN snp25032 for OA, LA, and OIL, *GmMTF* were predicted to be a candidate gene in Li et al. (2015); around a common QTN snp73103 for OA, LNA, and OIL, there were a known gene *GmSWEET39/GmSWEET10a* in Miao et al. (2020). These results revealed the correlation of seed oil-related traits.

### 3VmrMLM identifies more common QTNs in single and all the environments

For QTNs identified using 3VmrMLM in single environment, 76 QTNs were repeatedly identified, and 7, 13, 11, 8, 4, and 5 QTNs were repeatedly identified for PA, SA, OA, LA, LNA, and OIL, respectively. For example, snp25032 for LA was identified in NJ2011, NJ2012, NJ2015, WH2014, WH2015, and BLUP, with the LOD scores of 7.36 ~ 23.72 and r^2^ of 4.14% ~ 11.11%.

Compared the QTNs identified in single and all the environments, there were 68 common QTNs, and 13, 18, 19, 15, 6, and 9 common QTNs were associated with PA, SA, OA, LA, LNA, and OIL, respectively. For example, snp25032 for LA was identified with large LOD score and r^2^ in single and all the environments. These common QTNs should be more reliable. For example, snp22231 for PA was identified both in single and all the environment, and known gene *GmFATB1A* was around this QTN (**Table 1**; **Figure S2**); snp4406 for OA and LA was detected in multi-environment join analysis rather than single environment, and there was a known gene *GmDGAT2D* around this QTN (**Table 1**; **Figures S2**, **S3**). The above results showed that multiple environments join analysis not only detects some common QTNs with those in single environment, but also identify new QTNs in all the environments. Furthermore, some known genes regulated quantitative traits were around these new QTNs.

In summary, 598 QTNs and 54 QEIs for soybean seed oil-related traits were identified using 3VmrMLM in this study. Around these QTNs, seven known oil metabolism genes in soybean were identified. Around these QEIs, one oil metabolism soybean gene was verified by transgenic experiments in previous study; 5 genes were verified by transgenic experiments in other species to participate in oil metabolism and have different expressional levels across various environments, although new experiments are necessary to explore these novel GEI-trait associations in soybean. In addition, 76 candidate genes and 61 candidate GEIs were predicted by multi-omics method to be associated with seed oil-related traits. This study provided more gene resource for the breeding of high oil content soybean. Although available evidence clearly indicated these genes, the molecular mechanisms of how these genes underlying abiotic stress impacts on soybean seed oil content and fatty acid compositions remain unclear and need to further investigate.

## Data availability statement

The datasets presented in this study can be found in online repositories. The names of the repository/repositories and accession number(s) can be found in the article/**Supplementary Material**.

## Author contributions

Y-MZ conceived and managed the research and revised the manuscript. J-FZ and CG analyzed datasets. J-FZ and J-YL measured the phenotypes of the traits. J-FZ and YC mined the candidate oil metabolism genes. J-FZ wrote the draft. All authors contributed to the article and approved the submitted version.

## Funding

This work was supported by the National Natural Science Foundation of China (32070557; 31871242; 32270673), and Huazhong Agricultural University Scientific & Technological Self-Innovation Foundation (2014RC020).

## Conflict of interest

The authors declare that the research was conducted in the absence of any commercial or financial relationships that could be construed as a potential conflict of interest.

## Publisher’s note

All claims expressed in this article are solely those of the authors and do not necessarily represent those of their affiliated organizations, or those of the publisher, the editors and the reviewers. Any product that may be evaluated in this article, or claim that may be made by its manufacturer, is not guaranteed or endorsed by the publisher.
