# Supplementary material for: Identification of QTN-by-environment interactions and their candidate genes for soybean seed oil-related traits using 3VmrMLM
Source: Front Plant Sci. 2022 Dec 12;13:1096457. doi: 10.3389/fpls.2022.1096457 (PMC9792120; doi:10.3389/fpls.2022.1096457)
Supplement: Supplementary file 1 [file DataSheet_1.pdf]

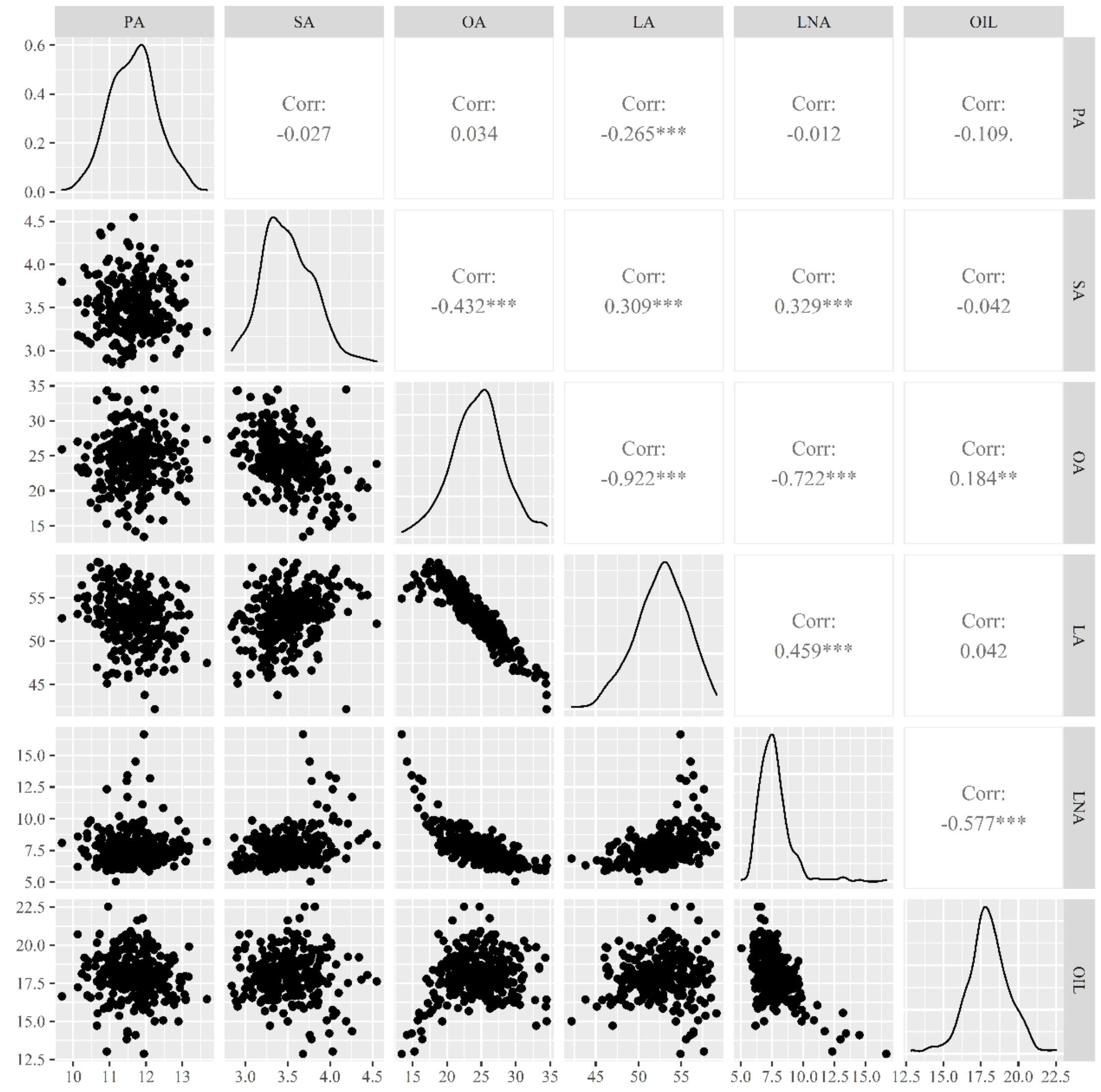

Figure S1. Simple correlation analysis for seed oil-related traits in 286 soybean accessions. The upper triangle lists correlation coefficient and its significance. The lower triangle is the scatter of BLUP value. The diagonal is density map of BLUP values. ** and ***: significance at the 0.01 and 0.001 probability levels, respectively.

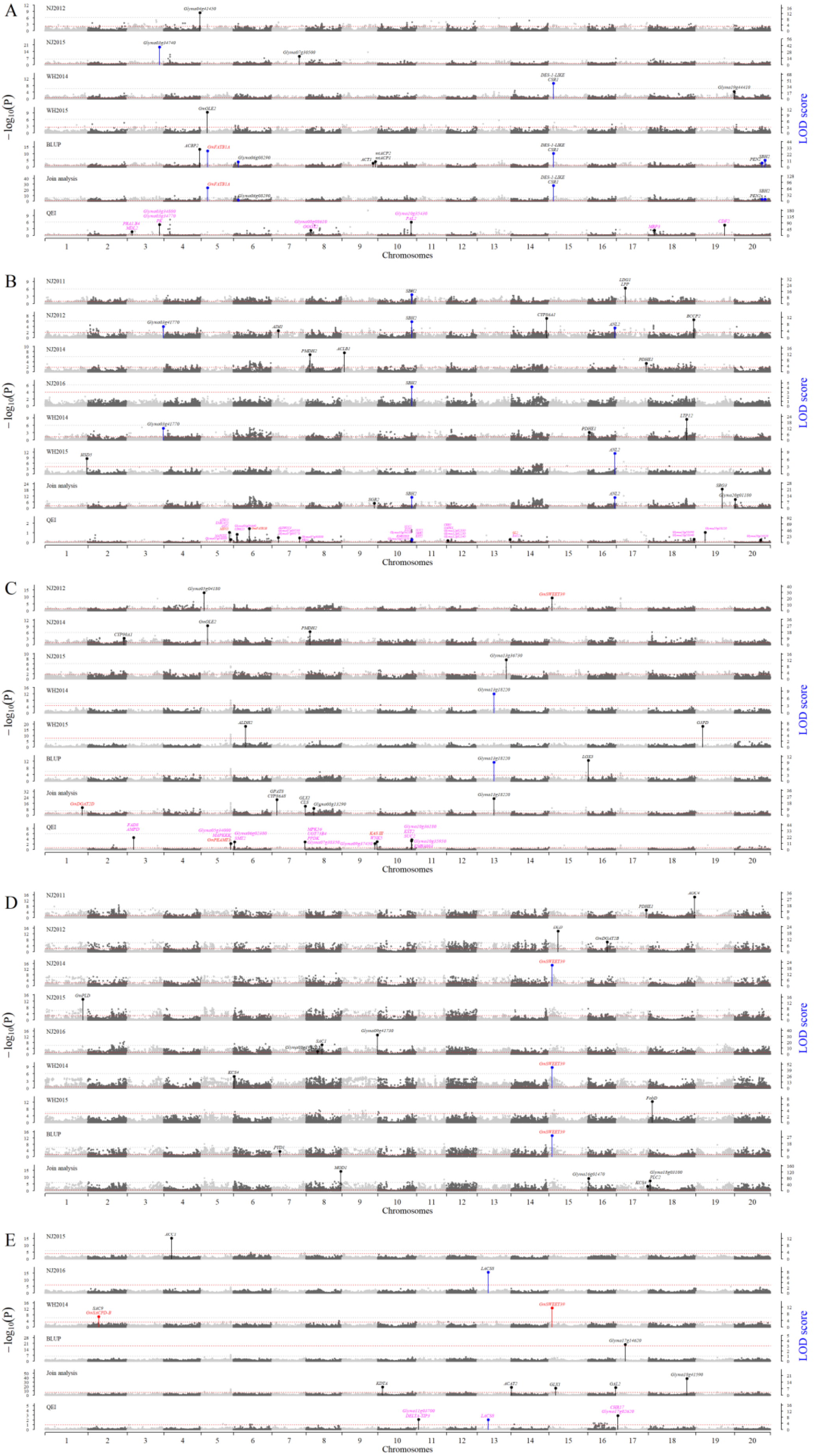
A
B
C
D
E
NJ2011
NJ2012
NJ2014
NJ2015
NJ2016
WH2014
WH2015
BLUP
Join analysis
QEI
−log10(P)
LOD score
Chromosomes

Figure S2. Manhattan plots for seed palmitic (A), stearic (B), oleic (C), linolenic (D) acids, and oil content (E) in soybean using 3VmrMLM. The small figures of NJ2011, NJ2012, NJ2014, NJ2015, NJ2016, WH2014 and WH2015 were QTNs, detected using single environment module in software IIIVmrMLM. Join analysis and QEIs were QTNs and QTN-by-environment interactions (QEIs), respectively, detected using multi-environment joint analysis module in software IIIVmrMLM. The black (one) and blue (multiple) lines indicate the number of times that the QTN/QEI was identified. Known genes, candidate genes, and gene-by-environment interactions (GEIs) were marked with red, black, and magenta colors, respectively.

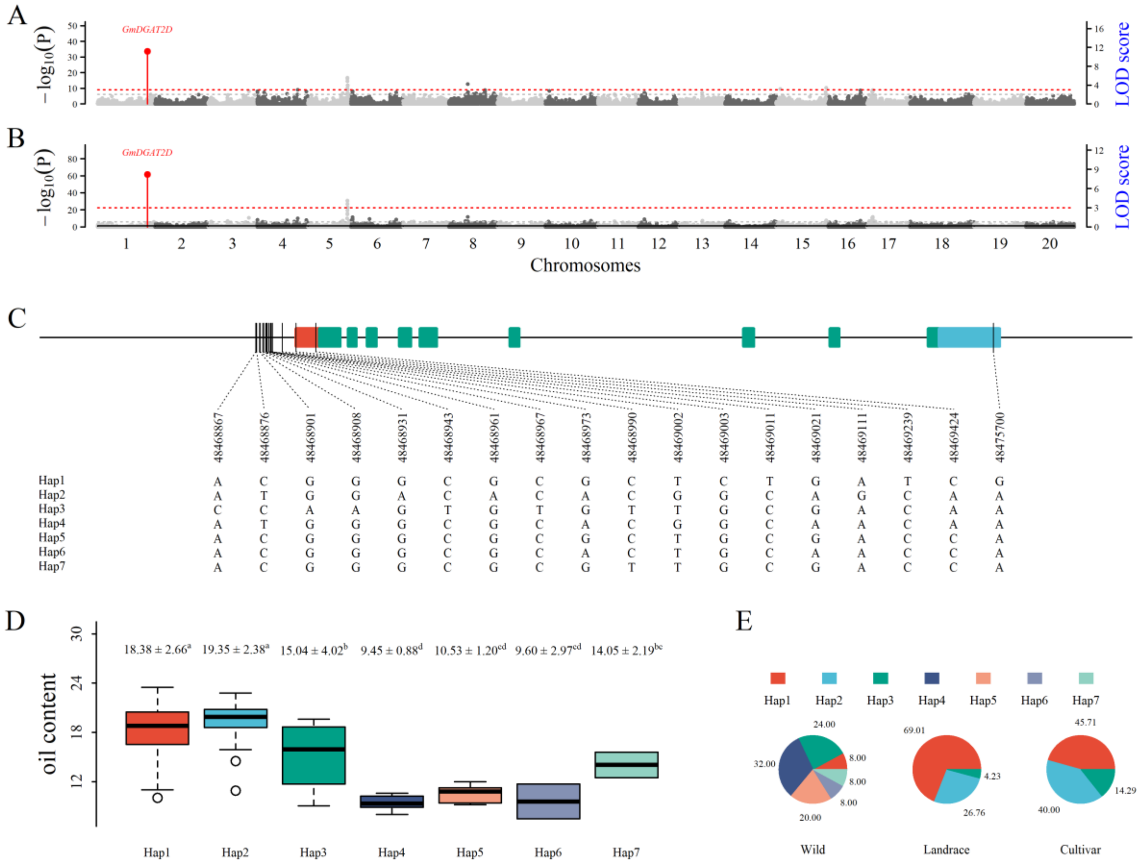

Figure S3. Haplotype analysis of candidate gene *GmDGAT2D* around QTN for soybean seed oil-related traits. (A): Manhattan plot for OA using multi-environment joint analysis. (B): Manhattan plot for LA using multi-environment joint analysis. (C): Eighteen SNPs and their haplotypes of *GmDGAT2D*. (D): The averages for seed oil content of seven *GmDGAT2D* haplotypes and their significances via multiple comparisons at the 0.05 probability level. (E): The haplotype frequencies of *GmDGAT2D* in wild, landrace, and bred soybeans.

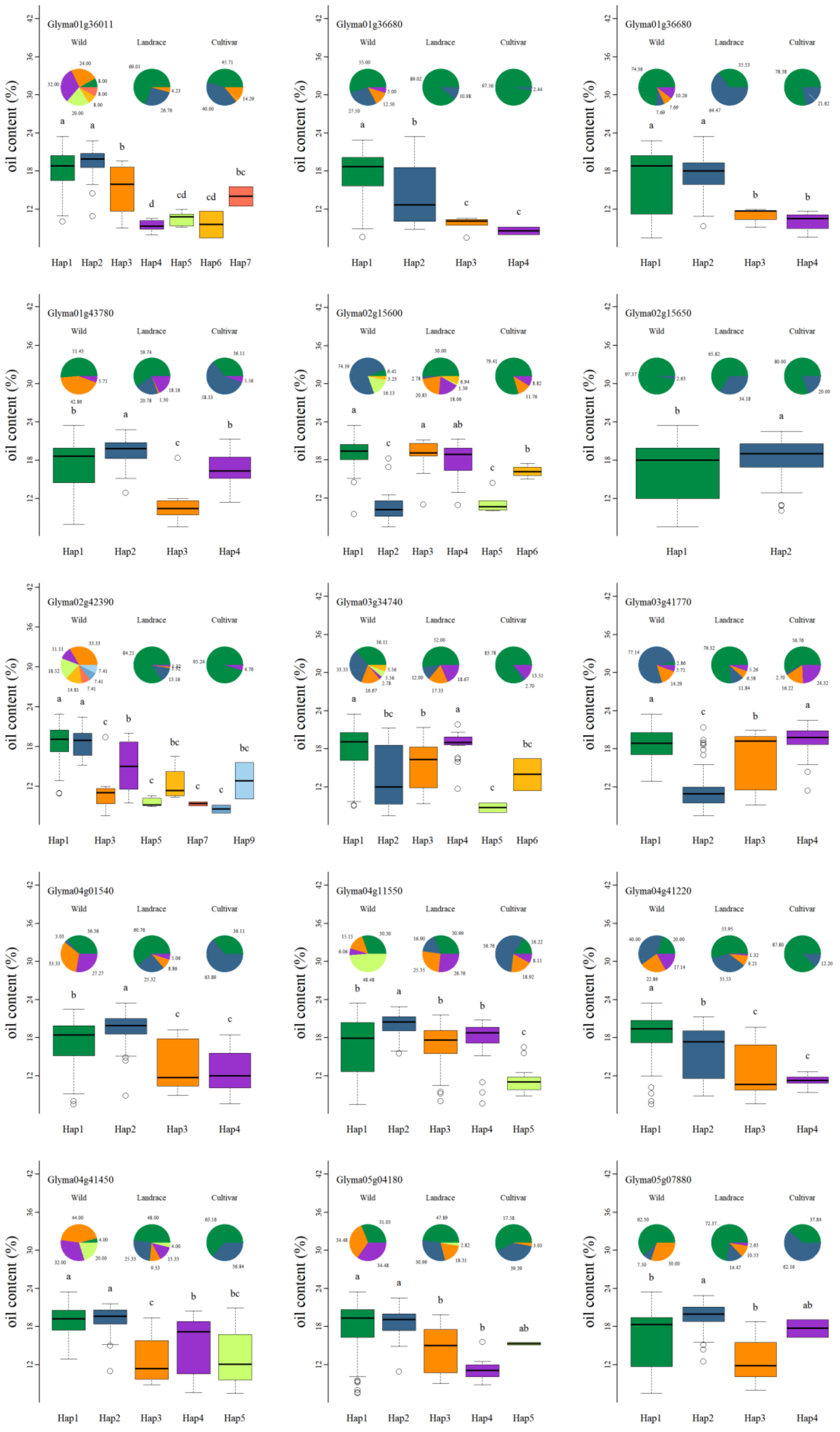

oil content (%)
Glyma01g36011
Wild
Landrace
Cultivar
Hap1 Hap2 Hap3 Hap4 Hap5 Hap6 Hap7
Glyma01g36680
Wild
Landrace
Cultivar
Hap1 Hap2 Hap3 Hap4
Glyma01g36680
Wild
Landrace
Cultivar
Hap1 Hap2 Hap3 Hap4
Glyma01g43780
Wild
Landrace
Cultivar
Hap1 Hap2 Hap3 Hap4
Glyma02g15600
Wild
Landrace
Cultivar
Hap1 Hap2 Hap3 Hap4 Hap5 Hap6
Glyma02g15650
Wild
Landrace
Cultivar
Hap1 Hap2
Glyma02g42390
Wild
Landrace
Cultivar
Hap1 Hap3 Hap5 Hap7 Hap9
Glyma03g34740
Wild
Landrace
Cultivar
Hap1 Hap2 Hap3 Hap4 Hap5 Hap6
Glyma03g41770
Wild
Landrace
Cultivar
Hap1 Hap2 Hap3 Hap4
Glyma04g01540
Wild
Landrace
Cultivar
Hap1 Hap2 Hap3 Hap4
Glyma04g11550
Wild
Landrace
Cultivar
Hap1 Hap2 Hap3 Hap4 Hap5
Glyma04g41220
Wild
Landrace
Cultivar
Hap1 Hap2 Hap3 Hap4
Glyma04g41450
Wild
Landrace
Cultivar
Hap1 Hap2 Hap3 Hap4 Hap5
Glyma05g04180
Wild
Landrace
Cultivar
Hap1 Hap2 Hap3 Hap4 Hap5
Glyma05g07880
Wild
Landrace
Cultivar
Hap1 Hap2 Hap3 Hap4

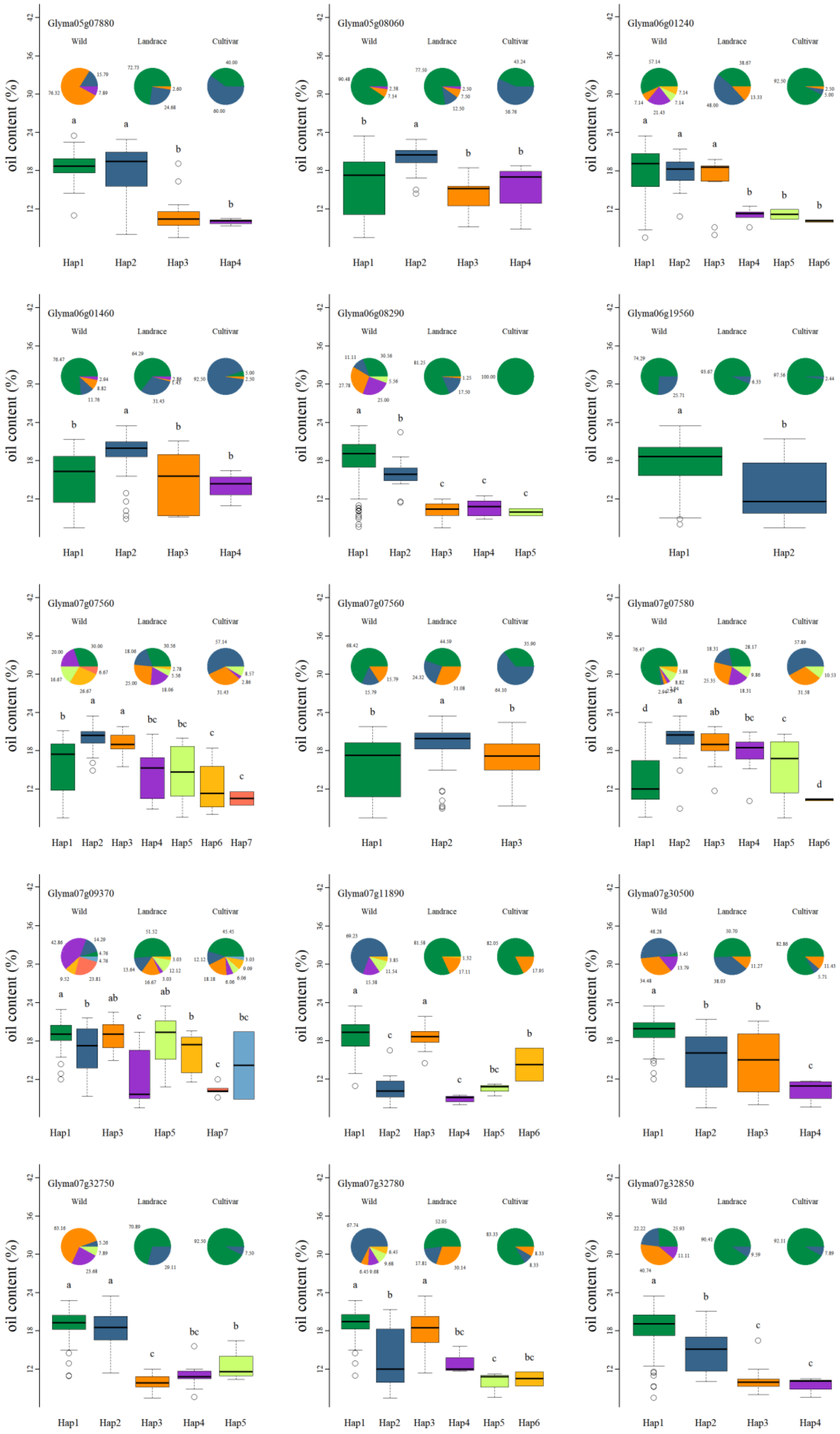
Glyma05g07880
Glyma05g08060
Glyma06g01240
Glyma06g01460
Glyma06g08290
Glyma06g19560
Glyma07g07560
Glyma07g07560
Glyma07g07580
Glyma07g09370
Glyma07g11890
Glyma07g30500
Glyma07g32750
Glyma07g32780
Glyma07g32850
Wild
Landrace
Cultivar
oil content (%)
Hap1
Hap2
Hap3
Hap4
Hap5
Hap6
Hap7

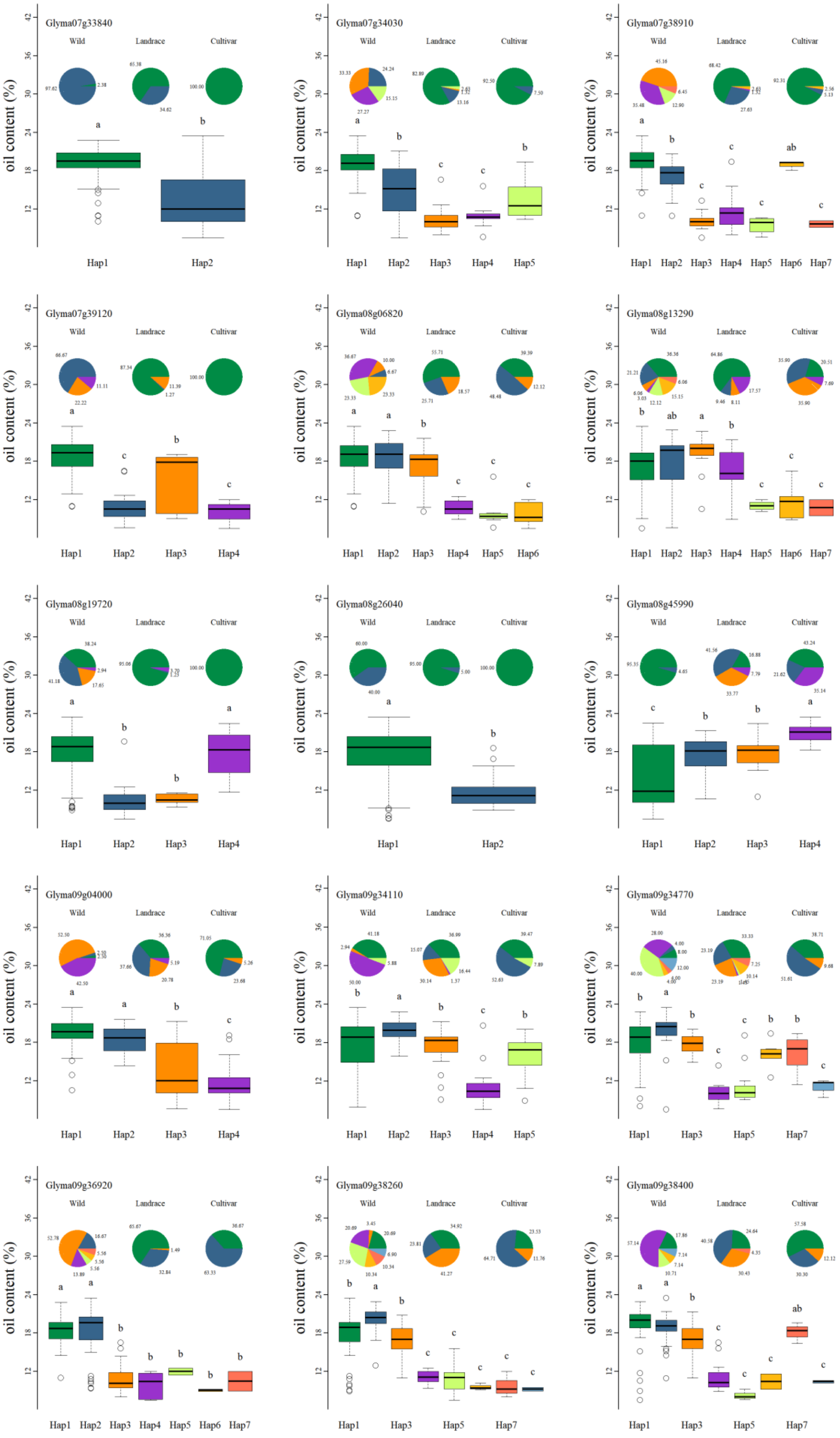
Glyma07g33840
Glyma07g34030
Glyma07g38910
Glyma07g39120
Glyma08g06820
Glyma08g13290
Glyma08g19720
Glyma08g26040
Glyma08g45990
Glyma09g04000
Glyma09g34110
Glyma09g34770
Glyma09g36920
Glyma09g38260
Glyma09g38400
Wild
Landrace
Cultivar
oil content (%)
Hap1
Hap2
Hap3
Hap4
Hap5
Hap6
Hap7

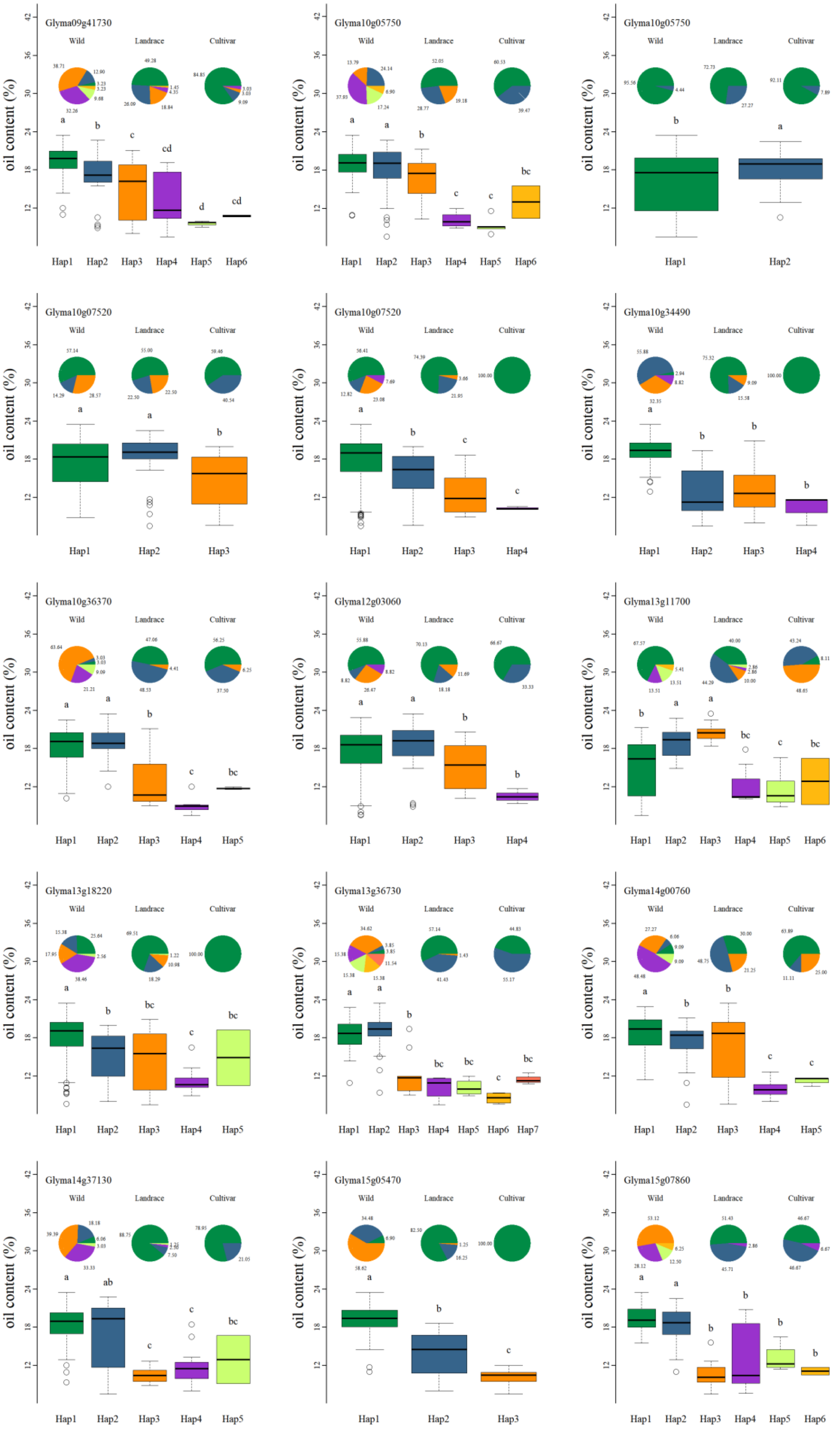
Glyma09g41730
Glyma10g05750
Glyma10g05750
Glyma10g07520
Glyma10g07520
Glyma10g34490
Glyma10g36370
Glyma12g03060
Glyma13g11700
Glyma13g18220
Glyma13g36730
Glyma14g00760
Glyma14g37130
Glyma15g05470
Glyma15g07860
Wild
Landrace
Cultivar
oil content (%)
Hap1
Hap2
Hap3
Hap4
Hap5
Hap6
Hap7

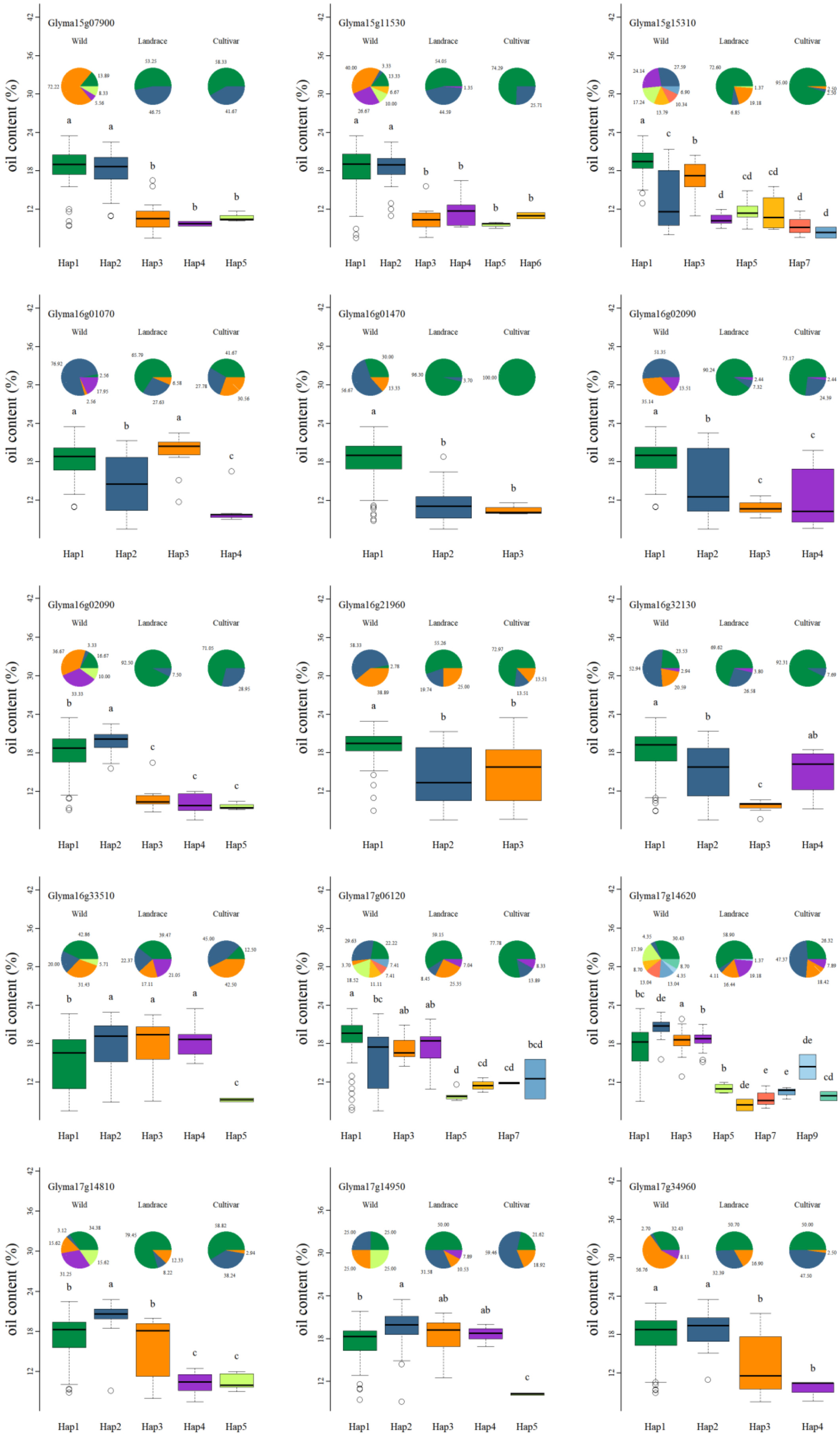

Glyma15g07900
Glyma15g11530
Glyma15g15310
Glyma16g01070
Glyma16g01470
Glyma16g02090
Glyma16g02090
Glyma16g21960
Glyma16g32130
Glyma16g33510
Glyma17g06120
Glyma17g14620
Glyma17g14810
Glyma17g14950
Glyma17g34960
Wild
Landrace
Cultivar
oil content (%)

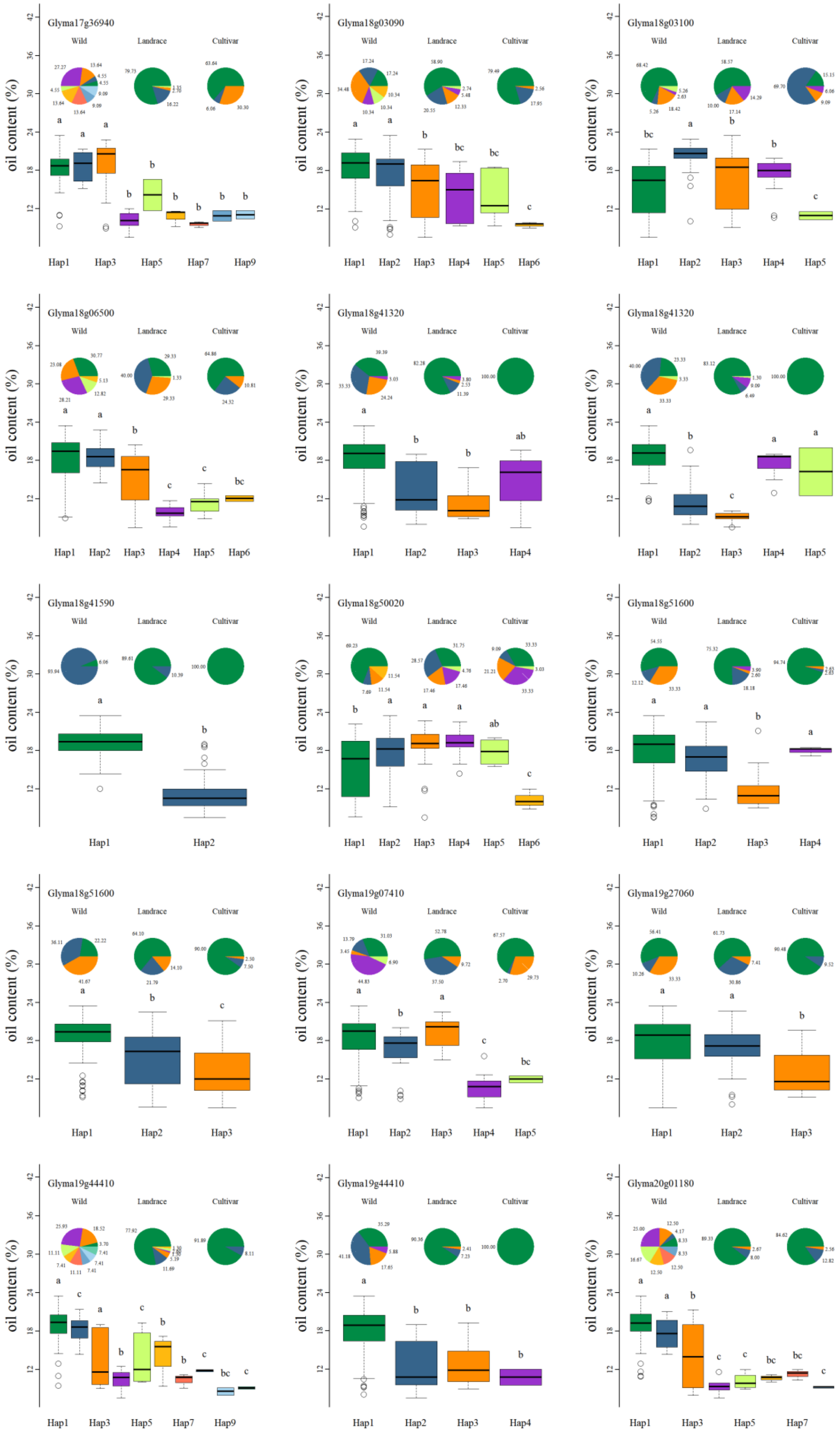
Glyma17g36940
Wild
Landrace
Cultivar
oil content (%)
Hap1 Hap3 Hap5 Hap7 Hap9
Glyma18g03090
Hap1 Hap2 Hap3 Hap4 Hap5 Hap6
Glyma18g03100
Hap1 Hap2 Hap3 Hap4 Hap5
Glyma18g06500
Hap1 Hap2 Hap3 Hap4 Hap5 Hap6
Glyma18g41320
Hap1 Hap2 Hap3 Hap4
Glyma18g41320
Hap1 Hap2 Hap3 Hap4 Hap5
Glyma18g41590
Hap1 Hap2
Glyma18g50020
Hap1 Hap2 Hap3 Hap4 Hap5 Hap6
Glyma18g51600
Hap1 Hap2 Hap3 Hap4
Glyma18g51600
Hap1 Hap2 Hap3
Glyma19g07410
Hap1 Hap2 Hap3 Hap4 Hap5
Glyma19g27060
Hap1 Hap2 Hap3
Glyma19g44410
Hap1 Hap3 Hap5 Hap7 Hap9
Glyma19g44410
Hap1 Hap2 Hap3 Hap4
Glyma20g01180
Hap1 Hap3 Hap5 Hap7

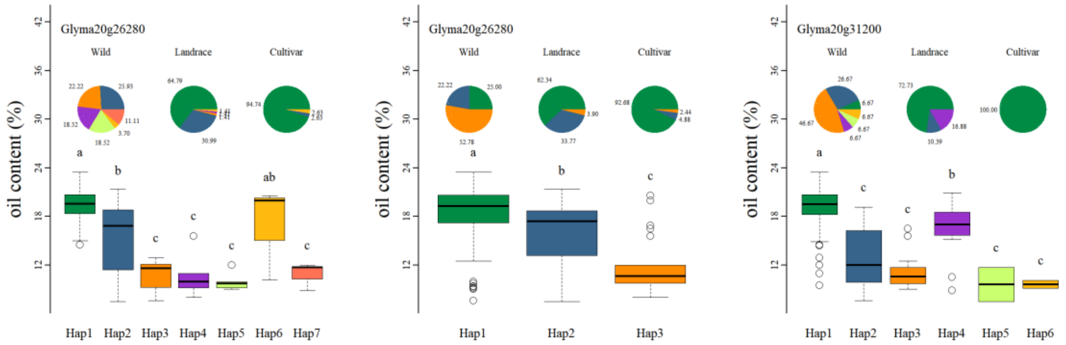

Figure S4. Haplotype analysis of 83 candidate oil metabolism genes for soybean seed oil-related traits. The haplotype hap1 ~ hap10 were marked with spring green, steel blue, dark orange, dark orchid, dark olive green, dark goldenrod, coral, sky blue, light sky blue and medium aquamarine. The letters a ~ e on the box plot indicated the haplotype were significant different via multiple comparisons.

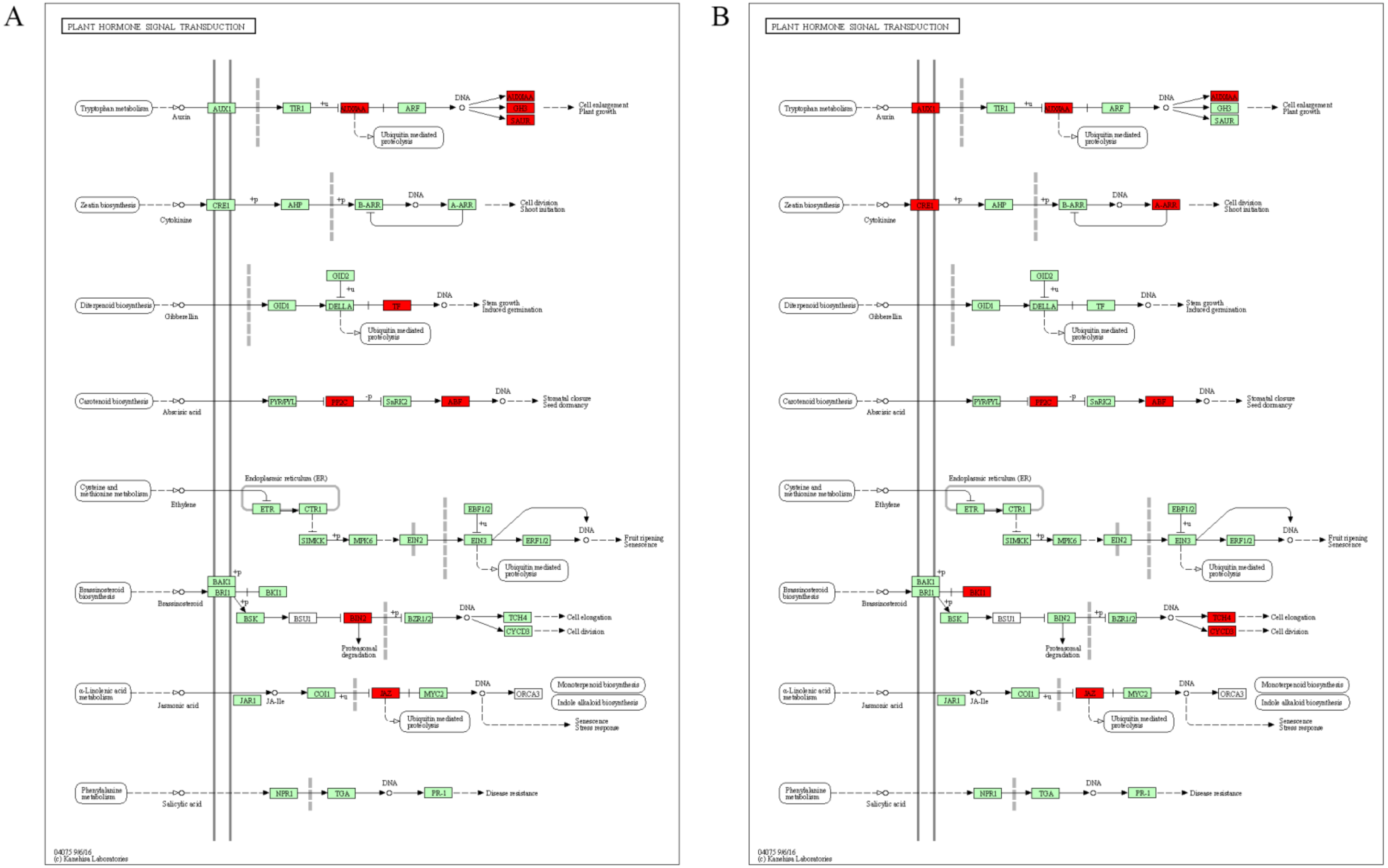

Figure S5. The genes involved in plant hormone signal transduction of KEGG pathways. (A): The genes under drought stress. (B): The genes under control condition. The input genes were marked with red color.

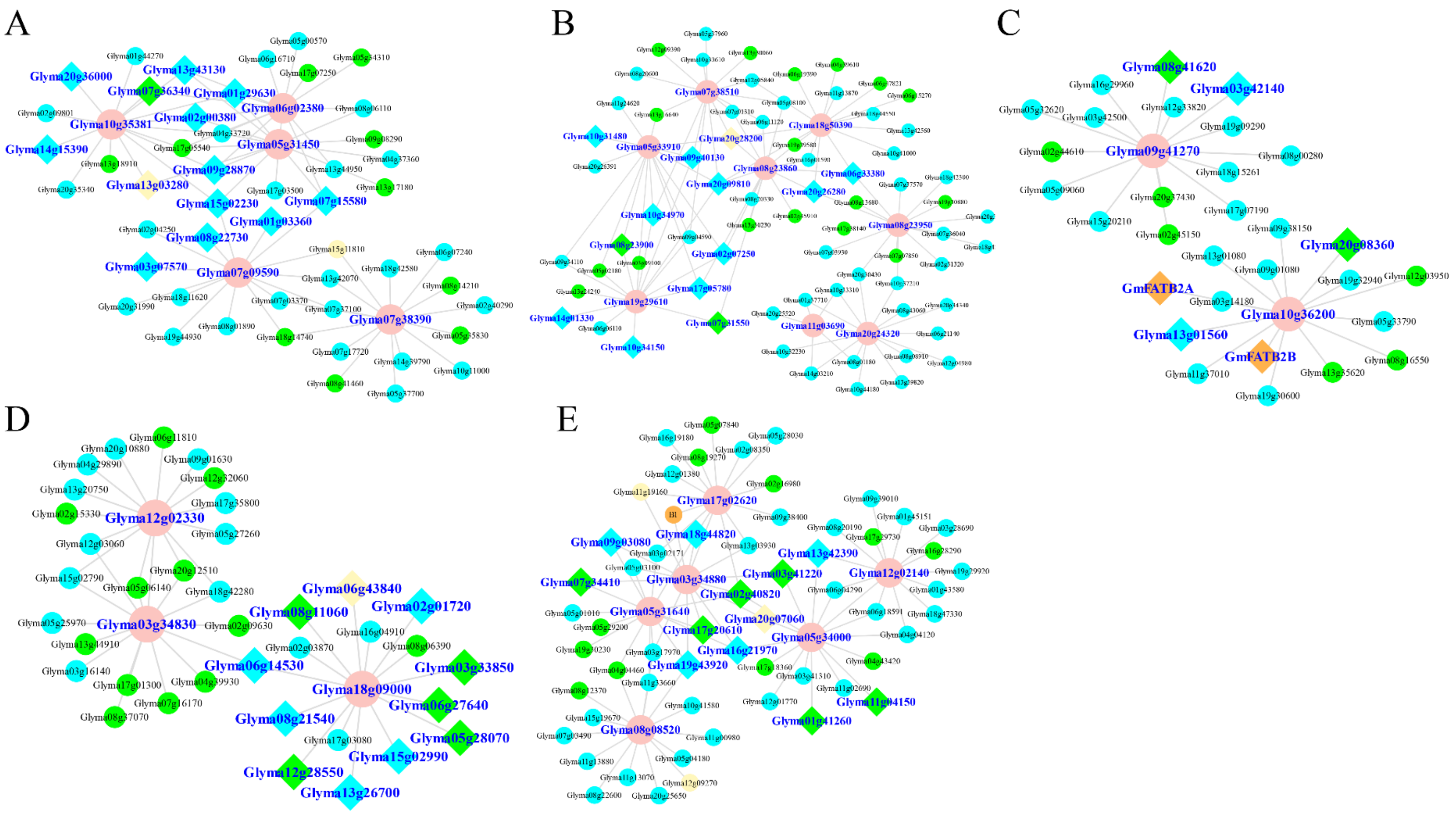

Figure S6. The subnetwork of candidate genes under control condition. (A-E): The blue, brown, green, turquoise and yellow module, respectively. The known oil genes, oil metabolism genes, drought response genes, genes involved in both oil metabolism and drought response, and candidate genes were marked with orange, blue, green, yellow, and plink colors, respectively. The hub genes of each module were marked diamond shape.

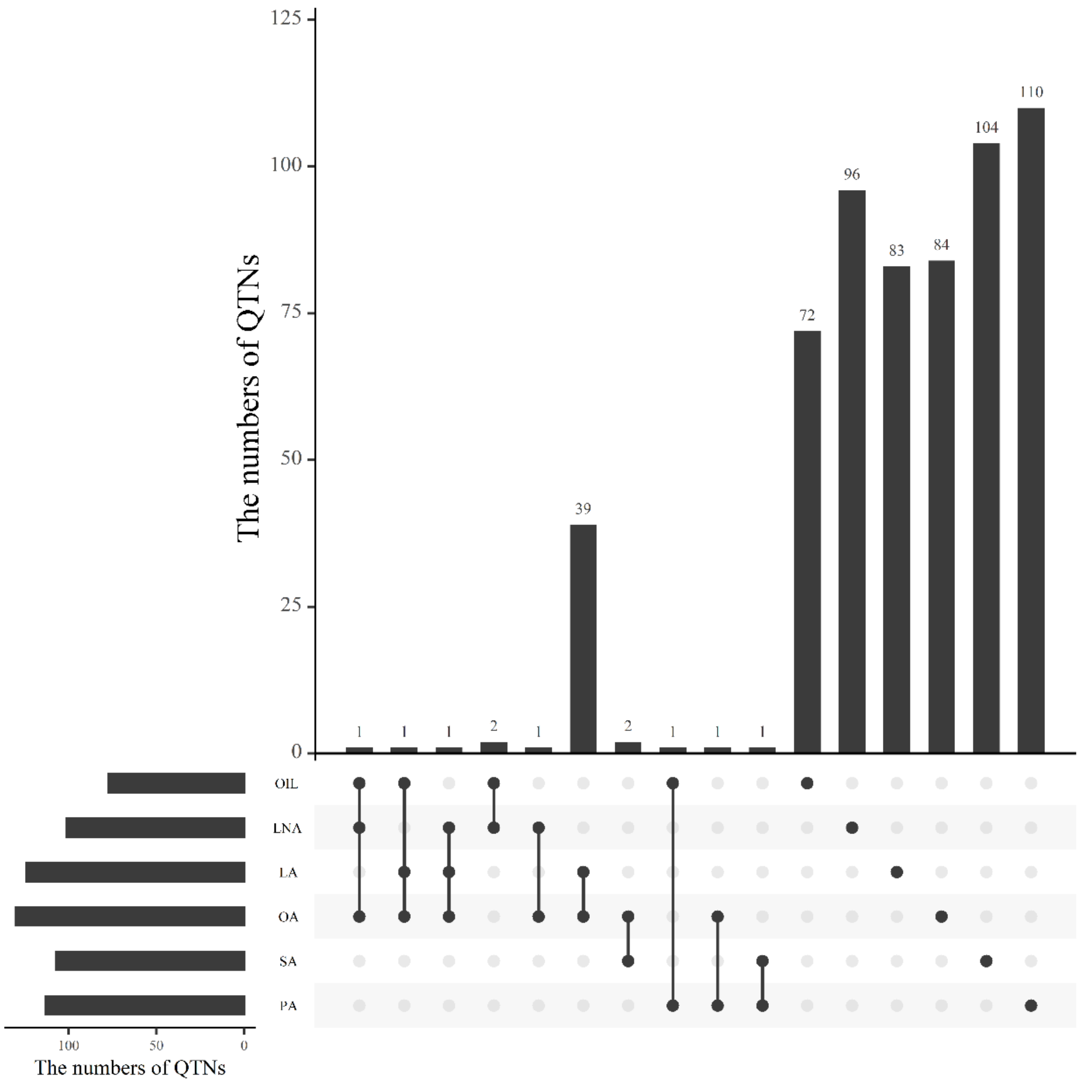

Figure S7. The number of common QTNs among soybean seed oil-related traits

Table S1. The summary of BLUP value for seed oil-related traits in 286 soybeans accessions

Trait	Mean	SD	Min	Max	Range	Skew	Kurtosis	CV
PA	11.66	0.65	9.69	13.67	3.98	0.06	-0.07	5.59
SA	3.51	0.30	2.84	4.55	1.71	0.45	0.20	8.67
OA	24.48	3.86	13.42	34.48	21.06	-0.02	0.17	15.76
LA	52.66	3.04	42.17	59.12	16.95	-0.37	-0.03	5.77
LNA	7.70	1.35	5.05	16.65	11.60	2.47	10.46	17.56
OIL	17.98	1.45	12.86	22.52	9.66	-0.14	1.15	8.05

PA: palmitic acid; SA: stearic acid; OA: oleic acid; LA: linoleic acid; LNA: linolenic acid; OIL: oil content; SD: standard deviation; Min: minimum; Max: maximum; CV: coefficient of variation; Skew: skewness; Kurtosis: kurtosis.

Table S2. Two-way (genotypes and environments) ANOVA for soybean seed oil-related traits

Trait	Genotype			Environment		
	DF	F value	P value	DF	F value	P value
PA	285	6.74	7.64E-142***	6	42.26	3.80E-48***
SA	285	8.29	6.08E-178***	6	24.48	6.63E-28***
OA	285	11.94	4.31E-252***	6	75.51	2.06E-83***
LA	285	11.53	1.37E-244***	6	65.41	4.73E-73***
LNA	285	20.07	<1.00E-300***	6	81.83	9.23E-90***
OIL	285	6.38	1.85E-111***	4	109.89	1.07E-78***

PA: palmitic acid (PA); SA: stearic acid; OA: oleic acid; LA: linoleic acid; LNA: linolenic acid; OIL: oil content; DF: degree of freedom; ***: significance at the 0.001 level. ns: no significance at the 0.05 level.

Table S3. Two-way (genotypes and years) ANOVA for soybean seed oil-related traits

Trait	Genotype			Year			Genotype × Year		
	DF	F value	P value	DF	F value	P value	DF	F value	P value
PA	285	5.29	1.03E-60***	4	41.91	1.80E-30***	1126	0.71	1.00E+00ns
SA	285	8.04	5.10E-92***	4	27.18	1.69E-20***	1126	0.98	6.14E-01ns
OA	285	8.53	8.17E-97***	4	68.53	1.42E-46***	1126	0.64	1.00E+00ns
LA	285	8.72	1.14E-98***	4	64.65	2.30E-44***	1126	0.68	1.00E+00ns
LNA	285	18.78	2.34E-168***	4	100.57	2.26E-63***	1126	1.00	4.88E-01ns
OIL	285	4.82	1.84E-54***	2	114.54	6.08E-42***	570	0.74	1.00E+00ns

PA: palmitic acid; SA: stearic acid; OA: oleic acid; LA: linoleic acid; LNA: linolenic acid; OIL: oil content; DF: degree of freedom; ***: significance at the 0.001 level. ns: no significance at the 0.05 level.

Table S4. Two-way (genotypes and locations) ANOVA for soybean seed oil-related traits

Trait	Genotype			Location			Genotype × Location		
	DF	F value	P value	DF	F value	P value	DF	F value	P value
PA	285	5.50	3.29E-103***	1	60.86	1.20E-14***	277	0.44	1.00E+00^{ns}
SA	285	7.49	7.79E-148***	1	1.33	2.49E-01^{ns}	277	0.88	9.04E-01^{ns}
OA	285	11.08	1.01E-215***	1	92.64	2.89E-21***	277	1.82	2.65E-12***
LA	285	10.64	3.53E-208***	1	12.75	3.68E-04***	277	1.79	1.10E-11***
LNA	285	17.17	2.14E-305***	1	336.44	2.22E-67***	277	0.76	9.98E-01^{ns}
OIL	285	4.29	2.06E-59***	1	5.69	1.73E-02*	277	0.77	9.95E-01^{ns}

PA: palmitic acid; SA: stearic acid; OA: oleic acid; LA: linoleic acid; LNA: linolenic acid; OIL: oil content; DF: degree of freedom; *, and ***: significance at the 0.05 and 0.001 level, respectively. ns: no significance at the 0.05 level.

Table S5. QTNs for soybean seed oil-related traits in a single environment using 3VmrMLM

Trait	Env	Marker	Chr	Pos (bp)	LOD	Add	Dom	Var	r^2(%)	P-value	Significance
PA	NJ2011	snp64	1	644991	10.58	-0.27	0.38	0.07	7.95	2.62E-11	SIG
PA	NJ2011	snp2359	1	28459059	10.88	0.29	-0.06	0.03	3.25	1.32E-11	SIG
PA	NJ2011	snp16592	4	5394126	4.48	0.18	-0.11	0.03	3.18	3.28E-05	SUG
PA	NJ2011	snp34260	7	17318313	8.81	-0.26	-0.03	0.06	6.72	1.56E-09	SIG
PA	NJ2011	snp48279	10	5170333	10.27	-0.28	0.02	0.04	4.19	5.36E-11	SIG
PA	NJ2011	snp50720	10	38230434	17.25	-0.37	0.19	0.06	6.29	5.67E-18	SIG
PA	NJ2011	snp70729	14	32377041	9.58	0.26		0.04	4.42	3.12E-11	SIG
PA	NJ2011	snp97710	19	27138371	12.62	-0.41	-0.26	0.04	4.51	2.42E-13	SIG
PA	NJ2011	snp103850	20	33174686	13.35	0.33		0.02	2.31	4.53E-15	SIG
PA	NJ2012	snp12835	3	22208216	25.34	0.43	-0.30	0.03	3.48	4.55E-26	SIG
PA	NJ2012	snp20863	4	47384506	12.56	-0.28	-0.17	0.03	3.28	2.74E-13	SIG
PA	NJ2012	snp22252	5	8146189	5.48	0.18	-0.28	0.02	2.24	3.31E-06	SUG
PA	NJ2012	snp32934	7	8506637	4.14	0.14	-0.33	0.03	2.49	7.24E-05	SUG
PA	NJ2012	snp44117	9	10787708	9.51	0.20	-0.58	0.06	5.96	3.08E-10	SIG
PA	NJ2012	snp46013	9	35905616	5.09	0.17	0.26	0.02	1.95	8.11E-06	SUG
PA	NJ2012	snp48240	10	4919971	9.25	-0.25	-0.10	0.04	4.29	5.68E-10	SIG
PA	NJ2012	snp51663	10	44968581	9.88	-0.25	0.33	0.06	6.27	1.31E-10	SIG
PA	NJ2012	snp60949	12	37256846	10.55	-0.26	0.22	0.06	5.75	2.84E-11	SIG
PA	NJ2012	snp68620	14	8295655	19.67	-0.37	0.29	0.06	5.68	2.13E-20	SIG
PA	NJ2012	snp100817	20	254816	9.91	0.25	0.04	0.02	2.22	1.24E-10	SIG
PA	NJ2014	snp16412	4	4138487	10.05	-0.30	0.72	0.12	9.72	8.95E-11	SIG
PA	NJ2014	snp23758	5	28271870	3.10	-0.06	0.28	0.03	2.38	7.98E-04	SUG
PA	NJ2014	snp24160	5	31539059	13.16	-0.38	-0.52	0.08	6.75	6.87E-14	SIG
PA	NJ2014	snp25804	6	2708047	7.85	-0.29	0.44	0.09	7.55	1.40E-08	SIG
PA	NJ2014	snp88715	18	4924159	4.66	-0.22	-0.26	0.05	3.99	2.21E-05	SUG
PA	NJ2014	snp89212	18	7902989	22.87	-0.55	0.00	0.12	10.42	1.34E-23	SIG
PA	NJ2014	snp90527	18	19364003	22.61	0.55		0.03	2.62	1.91E-24	SIG
PA	NJ2014	snp91938	18	40440905	6.13	-0.31	0.10	0.04	3.29	7.49E-07	SIG
PA	NJ2015	snp3486	1	42138015	4.05	-0.21	-0.41	0.05	2.10	8.84E-05	SUG
PA	NJ2015	snp9425	2	42056865	46.45	-0.91	-0.92	0.23	10.10	3.59E-47	SIG
PA	NJ2015	snp15168	3	42150254	38.67	-0.80		0.07	3.14	1.28E-40	SIG
PA	NJ2015	snp23758	5	28271870	6.72	-0.38	0.07	0.06	2.62	1.93E-07	SIG
PA	NJ2015	snp28716	6	21901859	14.57	0.42	0.80	0.11	4.92	2.71E-15	SIG
PA	NJ2015	snp32946	7	8579088	26.98	2.40	-0.56	0.09	3.78	1.04E-27	SIG
PA	NJ2015	snp34260	7	17318313	7.84	-0.32	0.25	0.10	4.27	1.44E-08	SIG
PA	NJ2015	snp35454	7	35530610	18.06	-0.43	1.82	0.17	7.77	8.80E-19	SIG
PA	NJ2015	snp43189	9	3663170	5.31	-0.06	-1.02	0.06	2.63	4.88E-06	SUG
PA	NJ2015	snp64268	13	25718852	34.90	0.75		0.08	3.42	7.93E-37	SIG
PA	NJ2015	snp87862	17	41079163	17.34	0.54	0.26	0.05	2.10	4.54E-18	SIG
PA	NJ2015	snp89203	18	7840442	11.22	-0.28	3.15	0.09	4.22	6.09E-12	SIG
PA	NJ2015	snp91409	18	29226972	6.75	0.13	1.47	0.06	2.70	1.78E-07	SIG

PA	NJ2015	snp98835	19	37280850	30.05	0.58	-4.11	0.17	7.52	8.97E-31	SIG
PA	NJ2016	snp16354	4	3807904	9.90	-0.03	-0.42	0.04	3.47	1.26E-10	SIG
PA	NJ2016	snp29954	6	40906027	4.37	-0.10	-0.66	0.04	3.34	4.25E-05	SUG
PA	NJ2016	snp38304	8	10788784	3.24	-0.17	0.27	0.03	2.81	5.69E-04	SUG
PA	NJ2016	snp48397	10	6366379	4.34	0.21	-0.22	0.04	3.08	4.61E-05	SUG
PA	NJ2016	snp50419	10	35128796	9.25	-0.33	0.16	0.10	8.83	5.68E-10	SIG
PA	NJ2016	snp53737	11	8085669	6.72	0.28	-0.28	0.08	6.57	1.90E-07	SIG
PA	NJ2016	snp70315	14	27529936	11.53	-0.18	0.40	0.06	5.32	2.93E-12	SIG
PA	NJ2016	snp88139	18	1060637	4.11	0.07	-0.75	0.04	3.45	7.84E-05	SUG
PA	NJ2016	snp101407	20	4667549	13.38	0.39	0.14	0.07	6.01	4.21E-14	SIG
PA	NJ2016	snp101454	20	5276539	5.94	0.08	1.50	0.05	4.29	1.15E-06	SUG
PA	WH2014	snp585	1	3879470	5.18	-0.11	0.31	0.02	2.93	6.62E-06	SUG
PA	WH2014	snp13793	3	33101336	8.55	0.17		0.02	3.97	3.49E-10	SIG
PA	WH2014	snp15508	3	44958196	24.79	-0.30	-0.35	0.06	10.17	1.62E-25	SIG
PA	WH2014	snp22252	5	8146189	9.71	0.16	-0.37	0.02	3.98	1.96E-10	SIG
PA	WH2014	snp39873	8	20845836	17.64	-0.24	-0.30	0.02	2.98	2.30E-18	SIG
PA	WH2014	snp41568	8	41311301	4.30	-0.11	0.16	0.01	2.34	5.00E-05	SUG
PA	WH2014	snp63687	13	22045116	12.54	0.19	0.41	0.04	7.56	2.92E-13	SIG
PA	WH2014	snp73350	15	5592008	43.32	-0.46	0.11	0.04	6.24	4.77E-44	SIG
PA	WH2014	snp74943	15	15461535	7.32	-0.06	0.83	0.02	4.02	4.75E-08	SIG
PA	WH2014	snp75812	15	22230281	13.50	-0.21	-0.18	0.04	7.57	3.14E-14	SIG
PA	WH2014	snp81887	16	27024859	8.49	-0.16	0.35	0.02	3.54	3.23E-09	SIG
PA	WH2014	snp94728	18	58872727	17.85	-0.26	0.24	0.02	3.04	1.42E-18	SIG
PA	WH2014	snp100677	19	49890890	20.58	-0.27		0.01	2.06	2.13E-22	SIG
PA	WH2014	snp103083	20	26279393	40.23	0.43		0.02	3.19	3.44E-42	SIG
PA	WH2015	snp10230	2	47545567	14.28	-0.25	-0.38	0.02	3.57	5.29E-15	SIG
PA	WH2015	snp22184	5	7711523	10.58	0.22	-0.18	0.02	2.58	2.62E-11	SIG
PA	WH2015	snp30709	6	46172445	9.20	-0.20	0.21	0.01	2.26	6.30E-10	SIG
PA	WH2015	snp32461	7	6439407	4.93	0.15	-0.02	0.02	3.18	1.17E-05	SUG
PA	WH2015	snp32879	7	8255337	10.79	0.22	-0.42	0.01	1.95	1.63E-11	SIG
PA	WH2015	snp42737	9	887317	3.73	-0.13	0.11	0.02	2.48	1.84E-04	SUG
PA	WH2015	snp43052	9	2803541	27.22	-0.38	0.09	0.03	5.48	6.01E-28	SIG
PA	WH2015	snp57241	12	2274173	3.98	0.00	-0.61	0.02	2.60	1.04E-04	SUG
PA	WH2015	snp64781	13	28702709	4.82	-0.15	0.02	0.01	1.98	1.52E-05	SUG
PA	WH2015	snp65526	13	32610399	18.72	0.30	0.14	0.02	2.71	1.90E-19	SIG
PA	WH2015	snp82805	16	31672357	4.65	-0.14	-0.06	0.02	2.46	2.22E-05	SUG
PA	WH2015	snp83615	16	36773852	5.18	0.15	-0.06	0.01	2.31	6.65E-06	SUG
PA	WH2015	snp84552	17	6859690	5.39	-0.16	0.00	0.01	2.24	4.11E-06	SUG
PA	WH2015	snp90138	18	16117202	7.05	-0.17		0.03	4.57	1.21E-08	SIG
PA	BLUP	snp372	1	2607025	28.32	-0.27		0.01	2.36	3.30E-30	SIG
PA	BLUP	snp20788	4	47019480	30.68	-0.28	0.10	0.02	4.13	2.09E-31	SIG
PA	BLUP	snp22231	5	8057446	28.06	0.27	-0.12	0.01	3.19	8.77E-29	SIG
PA	BLUP	snp26308	6	6123748	8.96	0.14	0.05	0.02	3.97	1.11E-09	SIG
PA	BLUP	snp44001	9	9517059	8.51	0.13	-0.21	0.02	3.67	3.11E-09	SIG

PA	BLUP	snp46685	9	40624897	6.75	-0.13	-0.05	0.01	3.16	1.80E-07	SIG
PA	BLUP	snp47176	9	43672155	9.48	-0.14	0.13	0.02	4.83	3.31E-10	SIG
PA	BLUP	snp47385	9	45304115	16.99	0.19	-0.32	0.02	4.31	1.02E-17	SIG
PA	BLUP	snp63687	13	22045116	8.27	0.01	0.56	0.02	4.18	5.32E-09	SIG
PA	BLUP	snp73350	15	5592008	23.50	-0.25	0.43	0.01	3.40	3.19E-24	SIG
PA	BLUP	snp74009	15	9912300	10.28	-0.15	-0.08	0.02	4.37	5.31E-11	SIG
PA	BLUP	snp79590	16	5466059	8.03	0.13		0.00	1.13	1.18E-09	SIG
PA	BLUP	snp83615	16	36773852	7.78	0.13	0.02	0.01	2.39	1.67E-08	SIG
PA	BLUP	snp97710	19	27138371	19.59	-0.26	-0.20	0.02	4.31	2.59E-20	SIG
PA	BLUP	snp104335	20	35860893	7.24	0.04	0.44	0.01	2.97	5.81E-08	SIG
PA	BLUP	snp104975	20	39923283	11.78	-0.10	-0.44	0.02	5.53	1.66E-12	SIG
SA	NJ2011	snp20383	4	44245421	8.24	0.10	-0.06	0.01	3.02	5.70E-09	SIG
SA	NJ2011	snp24856	5	37004968	23.42	-0.17	0.09	0.01	3.14	3.83E-24	SIG
SA	NJ2011	snp42213	8	44252695	6.24	0.08	0.00	0.01	3.15	5.74E-07	SIG
SA	NJ2011	snp46717	9	40842602	8.90	0.09	-0.23	0.01	4.63	1.25E-09	SIG
SA	NJ2011	snp51590	10	44469282	11.49	-0.12	-0.05	0.01	5.50	3.21E-12	SIG
SA	NJ2011	snp53496	11	6461699	7.98	0.10	-0.07	0.01	3.88	1.04E-08	SIG
SA	NJ2011	snp54603	11	16062768	5.78	0.08	-0.04	0.01	3.03	1.66E-06	SUG
SA	NJ2011	snp57897	12	6340883	11.21	-0.11	0.14	0.01	6.17	6.14E-12	SIG
SA	NJ2011	snp60824	12	36493003	11.59	-0.12	-0.26	0.01	3.21	2.57E-12	SIG
SA	NJ2011	snp67886	14	3441497	7.93	-0.09	-0.03	0.01	3.11	1.17E-08	SIG
SA	NJ2011	snp82676	16	31027487	7.68	0.09	0.12	0.01	3.71	2.08E-08	SIG
SA	NJ2011	snp82796	16	31568726	14.27	0.12	0.39	0.01	2.88	5.33E-15	SIG
SA	NJ2011	snp85152	17	11602669	20.31	-0.16	0.09	0.01	2.49	4.90E-21	SIG
SA	NJ2011	snp99919	19	44619057	7.79	0.10	0.10	0.01	3.07	1.61E-08	SIG
SA	NJ2012	snp408	1	2755382	6.84	-0.07	-0.05	0.01	3.98	1.46E-07	SIG
SA	NJ2012	snp15761	3	47212539	6.66	0.07	0.09	0.00	2.38	2.18E-07	SIG
SA	NJ2012	snp20369	4	44219419	9.19	0.09	-0.08	0.01	4.74	6.53E-10	SIG
SA	NJ2012	snp24600	5	35101815	4.85	-0.06	-0.03	0.00	2.76	1.43E-05	SUG
SA	NJ2012	snp28716	6	21901859	5.10	-0.06	-0.03	0.00	1.74	8.02E-06	SUG
SA	NJ2012	snp32787	7	7895362	4.24	0.06	-0.11	0.00	2.67	5.78E-05	SUG
SA	NJ2012	snp33154	7	9746352	16.52	0.12	0.03	0.00	2.46	2.99E-17	SIG
SA	NJ2012	snp41132	8	38405662	5.00	-0.01	0.42	0.00	2.90	1.00E-05	SUG
SA	NJ2012	snp45990	9	35713881	8.02	0.08	0.02	0.01	4.82	9.66E-09	SIG
SA	NJ2012	snp51590	10	44469282	9.49	-0.09	-0.06	0.01	4.95	3.21E-10	SIG
SA	NJ2012	snp71936	14	46333223	11.35	-0.09	-0.22	0.00	2.87	4.48E-12	SIG
SA	NJ2012	snp74854	15	14962581	7.33	0.08	0.08	0.00	3.55	4.70E-08	SIG
SA	NJ2012	snp83303	16	35331833	5.76	0.07	0.09	0.00	2.65	1.72E-06	SUG
SA	NJ2012	snp88986	18	6594001	3.37	-0.05	0.09	0.00	2.09	4.31E-04	SUG
SA	NJ2012	snp94841	18	59249935	10.56	-0.06	0.49	0.01	5.47	2.74E-11	SIG
SA	NJ2012	snp99919	19	44619057	13.74	0.11	0.05	0.01	6.60	1.81E-14	SIG
SA	NJ2014	snp16423	4	4177327	5.20	-0.07	0.11	0.01	2.47	6.25E-06	SUG
SA	NJ2014	snp16495	4	4780999	11.24	-0.11	-0.01	0.01	3.03	5.71E-12	SIG
SA	NJ2014	snp19355	4	37164512	9.37	-0.03	-0.34	0.01	3.68	4.29E-10	SIG

SA	NJ2014	snp20369	4	44219419	5.01	0.07	-0.01	0.00	1.91	9.75E-06	SUG
SA	NJ2014	snp28660	6	21403869	13.88	-0.11	0.29	0.01	5.42	1.31E-14	SIG
SA	NJ2014	snp37412	8	4895288	11.57	0.11	0.00	0.00	1.57	2.72E-12	SIG
SA	NJ2014	snp43067	9	2918055	12.98	-0.12	-0.12	0.01	5.29	1.04E-13	SIG
SA	NJ2014	snp45091	9	26301341	7.23	-0.09	-0.02	0.00	1.54	5.92E-08	SIG
SA	NJ2014	snp50203	10	33433543	12.37	0.05	0.72	0.01	4.96	4.28E-13	SIG
SA	NJ2014	snp57885	12	6270764	8.24	-0.09	-0.04	0.01	3.99	5.75E-09	SIG
SA	NJ2014	snp71149	14	40769103	13.86	-0.12	0.15	0.01	4.31	1.39E-14	SIG
SA	NJ2014	snp72923	15	2496058	7.82	0.08	-0.15	0.01	3.86	1.52E-08	SIG
SA	NJ2014	snp87436	17	38343914	5.98	-0.08	0.08	0.01	2.52	1.06E-06	SUG
SA	NJ2014	snp87552	17	38989203	5.76	-0.08	-0.08	0.00	1.68	1.73E-06	SUG
SA	NJ2014	snp105892	20	45832145	12.60	0.11	0.14	0.01	5.99	2.51E-13	SIG
SA	NJ2015	snp3600	1	43054301	5.86	0.11	-0.12	0.01	4.90	1.38E-06	SUG
SA	NJ2015	snp17208	4	9107467	5.08	0.03	0.34	0.01	3.78	8.31E-06	SUG
SA	NJ2015	snp29030	6	28231300	18.19	-0.21	0.06	0.03	13.13	6.50E-19	SIG
SA	NJ2015	snp34259	7	17318295	7.57	-0.13	-0.09	0.01	5.39	2.72E-08	SIG
SA	NJ2015	snp34515	7	19236178	12.86	-0.17	-0.12	0.01	6.20	1.38E-13	SIG
SA	NJ2015	snp66198	13	36854282	7.25	-0.09	0.17	0.02	6.71	5.62E-08	SIG
SA	NJ2015	snp77397	15	42763346	4.72	0.03	-0.46	0.01	4.11	1.89E-05	SUG
SA	NJ2015	snp88009	18	29117	8.64	-0.13	0.18	0.01	5.02	2.28E-09	SIG
SA	NJ2016	snp32803	7	7987723	3.95	0.10	0.00	0.01	4.22	1.12E-04	SUG
SA	NJ2016	snp51595	10	44535628	4.19	-0.10	0.10	0.01	4.45	6.49E-05	SUG
SA	NJ2016	snp60292	12	32790779	14.61	-0.19	-0.11	0.01	6.63	2.45E-15	SIG
SA	NJ2016	snp65981	13	35411064	9.45	-0.15	0.05	0.01	6.05	3.56E-10	SIG
SA	NJ2016	snp83221	16	34246889	5.84	0.15	0.05	0.01	5.61	1.46E-06	SUG
SA	NJ2016	snp87353	17	37865706	4.69	0.10	-0.08	0.01	5.01	2.04E-05	SUG
SA	WH2014	snp4553	1	49517900	16.60	0.14	-0.23	0.02	8.00	2.52E-17	SIG
SA	WH2014	snp15762	3	47243699	11.91	0.12	-0.03	0.01	2.91	1.23E-12	SIG
SA	WH2014	snp26121	6	4685918	13.76	-0.13	0.33	0.01	2.74	1.72E-14	SIG
SA	WH2014	snp30784	6	46730163	7.71	-0.09	0.05	0.00	1.68	1.97E-08	SIG
SA	WH2014	snp30999	6	48106897	5.02	0.01	-0.25	0.01	1.93	9.53E-06	SUG
SA	WH2014	snp43058	9	2816883	39.03	-0.25	0.20	0.02	6.31	9.43E-40	SIG
SA	WH2014	snp45101	9	26467734	8.52	-0.09	0.14	0.01	3.51	3.03E-09	SIG
SA	WH2014	snp45975	9	35606339	10.09	0.11	-0.06	0.01	3.43	8.20E-11	SIG
SA	WH2014	snp46711	9	40787234	3.31	-0.06	-0.03	0.00	1.34	4.93E-04	SUG
SA	WH2014	snp47717	10	1029109	19.83	-0.16	-0.17	0.01	3.65	1.47E-20	SIG
SA	WH2014	snp55265	11	24417809	3.41	0.06	-0.06	0.00	1.37	3.90E-04	SUG
SA	WH2014	snp57864	12	6104790	7.38	-0.09	-0.03	0.01	3.06	4.13E-08	SIG
SA	WH2014	snp68616	14	8289482	7.61	-0.10	0.03	0.01	2.90	2.47E-08	SIG
SA	WH2014	snp78912	16	1594037	7.73	0.00	0.46	0.01	3.17	1.87E-08	SIG
SA	WH2014	snp79529	16	5172417	20.65	-0.16	-0.30	0.01	2.47	2.26E-21	SIG
SA	WH2014	snp92846	18	50082146	21.11	-0.17	-0.07	0.01	4.23	7.80E-22	SIG
SA	WH2015	snp5250	1	54716009	6.30	0.09	0.08	0.01	3.62	5.07E-07	SIG
SA	WH2015	snp17234	4	9245211	11.04	-0.12	0.09	0.01	4.98	9.19E-12	SIG

SA	WH2015	snp28660	6	21403869	9.24	-0.10	0.15	0.01	3.81	5.80E-10	SIG
SA	WH2015	snp41575	8	41336434	10.18	0.11	-0.05	0.01	4.27	6.61E-11	SIG
SA	WH2015	snp53496	11	6461699	5.65	0.08	-0.04	0.01	3.02	2.22E-06	SUG
SA	WH2015	snp55883	11	30957122	5.96	0.08	0.10	0.01	3.29	1.09E-06	SUG
SA	WH2015	snp67334	13	43935334	6.44	0.09		0.00	2.33	5.22E-08	SIG
SA	WH2015	snp71149	14	40769103	33.84	-0.23	0.10	0.03	13.65	1.46E-34	SIG
SA	WH2015	snp83298	16	35319982	8.45	0.09	-0.30	0.01	5.15	3.58E-09	SIG
SA	WH2015	snp85342	17	12802791	18.13	-0.16	0.05	0.01	5.09	7.41E-19	SIG
SA	BLUP	snp9451	2	42193331	4.16	0.04	0.02	0.00	1.40	6.93E-05	SUG
SA	BLUP	snp16495	4	4780999	14.18	-0.08	0.06	0.00	4.03	6.64E-15	SIG
SA	BLUP	snp18220	4	18887156	4.99	0.05	-0.03	0.00	2.15	1.02E-05	SUG
SA	BLUP	snp20369	4	44219419	4.09	0.04	-0.04	0.00	1.59	8.08E-05	SUG
SA	BLUP	snp30999	6	48106897	8.22	0.01	-0.20	0.00	3.62	6.08E-09	SIG
SA	BLUP	snp31148	6	49038769	6.27	0.05	0.07	0.00	1.88	5.36E-07	SIG
SA	BLUP	snp45101	9	26467734	4.52	-0.04	0.06	0.00	1.96	2.99E-05	SUG
SA	BLUP	snp45990	9	35713881	7.76	0.06	-0.02	0.00	3.69	1.76E-08	SIG
SA	BLUP	snp53498	11	6475294	16.28	-0.09	0.05	0.00	2.45	5.22E-17	SIG
SA	BLUP	snp55255	11	24371125	8.47	0.06	-0.03	0.00	3.85	3.37E-09	SIG
SA	BLUP	snp57864	12	6104790	10.50	-0.07	-0.04	0.00	4.92	3.16E-11	SIG
SA	BLUP	snp67886	14	3441497	8.61	-0.06	0.01	0.00	3.08	2.49E-09	SIG
SA	BLUP	snp71149	14	40769103	21.44	-0.10	0.05	0.01	6.19	3.61E-22	SIG
SA	BLUP	snp83221	16	34246889	4.86	0.06	0.00	0.00	2.22	1.39E-05	SUG
SA	BLUP	snp89619	18	11853399	12.64	-0.01	0.28	0.01	5.83	2.29E-13	SIG
SA	BLUP	snp92825	18	49852918	26.17	-0.12	-0.01	0.00	3.31	6.72E-27	SIG
SA	BLUP	snp99871	19	44283115	11.80	0.07	-0.22	0.00	3.95	1.57E-12	SIG
OA	NJ2011	snp5982	2	4110866	42.70	2.54	-0.49	1.31	4.69	2.03E-43	SIG
OA	NJ2011	snp9477	2	42382374	17.98	1.46	-1.86	2.10	7.54	1.05E-18	SIG
OA	NJ2011	snp14246	3	35978459	3.61	0.63	-0.68	0.39	1.41	2.45E-04	SUG
OA	NJ2011	snp16028	4	1524786	8.90	-1.02	0.02	0.98	3.53	1.27E-09	SIG
OA	NJ2011	snp24461	5	33879285	13.30	-0.34	8.12	1.50	5.40	5.00E-14	SIG
OA	NJ2011	snp30182	6	42784786	11.36	-1.12	-1.89	0.93	3.34	4.33E-12	SIG
OA	NJ2011	snp31390	7	99598	7.64	0.95	-0.32	0.38	1.36	2.31E-08	SIG
OA	NJ2011	snp53468	11	6225981	11.60	1.18	0.06	0.86	3.09	2.49E-12	SIG
OA	NJ2011	snp57257	12	2387406	6.50	-0.81	1.52	0.60	2.15	3.18E-07	SIG
OA	NJ2011	snp68010	14	4251041	14.43	-1.30	1.37	1.10	3.96	3.72E-15	SIG
OA	NJ2011	snp73422	15	6013922	20.05	-1.56	-1.61	0.58	2.10	8.84E-21	SIG
OA	NJ2011	snp74712	15	14043785	4.02	-0.66	-0.61	0.43	1.53	9.62E-05	SUG
OA	NJ2011	snp80932	16	18975130	22.89	1.66	-2.05	1.96	7.04	1.30E-23	SIG
OA	NJ2011	snp82395	16	29683087	10.36	-1.10	0.60	1.00	3.59	4.38E-11	SIG
OA	NJ2011	snp94377	18	57090843	7.44	0.96	0.11	0.87	3.11	3.62E-08	SIG
OA	NJ2011	snp100070	19	45536111	5.79	-0.78	-0.82	0.61	2.18	1.61E-06	SUG
OA	NJ2011	snp100081	19	45688463	9.82	-0.32	2.46	1.08	3.88	1.53E-10	SIG
OA	NJ2012	snp14246	3	35978459	3.40	0.53	0.29	0.27	1.90	4.02E-04	SUG
OA	NJ2012	snp16820	4	6684385	8.76	0.86	-0.43	0.40	2.83	1.75E-09	SIG

OA	NJ2012	snp21648	5	3363617	29.47	-1.69	-3.05	0.53	3.75	3.37E-30	SIG
OA	NJ2012	snp25633	6	1436603	5.65	0.69	-0.92	0.48	3.39	2.25E-06	SUG
OA	NJ2012	snp37472	8	5458823	4.26	-0.16	5.35	0.33	2.35	5.44E-05	SUG
OA	NJ2012	snp42071	8	43518470	5.84	0.70	-0.23	0.21	1.48	1.43E-06	SUG
OA	NJ2012	snp46874	9	41732530	5.35	-0.65	0.65	0.35	2.45	4.50E-06	SUG
OA	NJ2012	snp48397	10	6366379	8.53	-0.85	-0.26	0.51	3.59	2.96E-09	SIG
OA	NJ2012	snp53301	11	5071119	24.07	1.52	0.02	0.47	3.32	8.52E-25	SIG
OA	NJ2012	snp56162	11	33853486	10.95	1.00	0.06	0.29	2.04	1.12E-11	SIG
OA	NJ2012	snp73103	15	3855027	21.11	1.40	1.09	0.35	2.49	7.77E-22	SIG
OA	NJ2012	snp74705	15	14013456	11.33	0.94	-4.97	0.36	2.51	4.69E-12	SIG
OA	NJ2012	snp82395	16	29683087	9.98	-0.94	0.12	0.70	4.92	1.04E-10	SIG
OA	NJ2014	snp4822	1	51429468	5.55	0.87	-0.57	0.54	2.64	2.82E-06	SUG
OA	NJ2014	snp10218	2	47425835	9.32	1.13	1.00	0.53	2.62	4.78E-10	SIG
OA	NJ2014	snp10500	2	49370610	7.66	-0.23	-3.71	0.90	4.40	2.18E-08	SIG
OA	NJ2014	snp14303	3	36330316	9.71	-0.35	4.92	1.32	6.49	1.96E-10	SIG
OA	NJ2014	snp22209	5	7887398	27.35	2.09	1.08	0.67	3.26	4.48E-28	SIG
OA	NJ2014	snp37412	8	4895288	18.58	-1.68	0.12	0.73	3.60	2.64E-19	SIG
OA	NJ2014	snp49687	10	26715804	4.32	-0.74		0.34	1.64	8.21E-06	SUG
OA	NJ2014	snp52642	11	416072	7.93	0.96	-1.91	1.09	5.34	1.17E-08	SIG
OA	NJ2014	snp81975	16	27654752	5.57	0.88		0.38	1.87	4.05E-07	SIG
OA	NJ2014	snp84948	17	10221486	6.10	-0.87	1.33	0.72	3.54	7.91E-07	SIG
OA	NJ2014	snp92730	18	49114891	5.20	0.77	-1.78	0.70	3.44	6.28E-06	SUG
OA	NJ2014	snp98484	19	34856552	8.96	-1.16	0.34	0.81	3.98	1.11E-09	SIG
OA	NJ2015	snp10372	2	48653339	5.86	-0.78	-1.42	0.54	3.42	1.40E-06	SUG
OA	NJ2015	snp30327	6	43952755	5.85	-0.73	1.39	0.63	3.93	1.42E-06	SUG
OA	NJ2015	snp37099	8	2769635	6.11	-0.74	2.42	0.44	2.79	7.75E-07	SIG
OA	NJ2015	snp39472	8	18244147	4.57	-0.65	1.38	0.47	2.95	2.68E-05	SUG
OA	NJ2015	snp45845	9	34357146	7.53	-0.41	-4.36	0.75	4.69	2.97E-08	SIG
OA	NJ2015	snp46892	9	41827963	4.74	-0.72	-1.07	0.42	2.66	1.82E-05	SUG
OA	NJ2015	snp53033	11	3180237	13.03	1.28	-0.84	0.21	1.32	9.36E-14	SIG
OA	NJ2015	snp66380	13	38054635	12.32	1.12	-1.73	1.36	8.52	4.80E-13	SIG
OA	NJ2015	snp88791	18	5426595	30.87	2.03	-1.54	0.93	5.84	1.36E-31	SIG
OA	NJ2015	snp96804	19	9057353	5.19	0.70	1.15	0.43	2.72	6.52E-06	SUG
OA	NJ2016	snp312	1	2312575	8.48	-0.51	4.19	1.14	4.43	3.31E-09	SIG
OA	NJ2016	snp4827	1	51457488	11.95	1.35	-0.50	1.42	5.48	1.11E-12	SIG
OA	NJ2016	snp15569	3	45409871	10.90	0.02	7.37	1.48	5.73	1.26E-11	SIG
OA	NJ2016	snp27546	6	14112644	23.18	-1.92	0.95	0.88	3.40	6.56E-24	SIG
OA	NJ2016	snp29567	6	38013620	7.95	1.08	-0.28	0.92	3.55	1.13E-08	SIG
OA	NJ2016	snp34353	7	17960593	4.05	-0.12	4.07	0.56	2.15	8.85E-05	SUG
OA	NJ2016	snp39463	8	18183299	5.03	-0.75	1.68	0.62	2.39	9.44E-06	SUG
OA	NJ2016	snp47711	10	981062	19.77	1.77	0.10	1.50	5.81	1.69E-20	SIG
OA	NJ2016	snp60569	12	34796251	6.68	-0.87	-1.70	0.85	3.30	2.08E-07	SIG
OA	NJ2016	snp66436	13	38327941	8.10	1.03	2.33	0.47	1.81	8.00E-09	SIG
OA	NJ2016	snp68007	14	4250998	8.65	-1.10	-1.63	0.63	2.42	2.22E-09	SIG

OA	NJ2016	snp72826	15	1932147	6.11	-0.92	-0.82	0.72	2.79	7.85E-07	SIG
OA	NJ2016	snp78114	15	47867395	4.93	0.85	-0.16	0.42	1.64	1.18E-05	SUG
OA	NJ2016	snp81975	16	27654752	12.55	1.38		0.93	3.62	2.91E-14	SIG
OA	WH2014	snp15838	4	75684	6.48	1.27	-0.12	0.98	1.93	3.33E-07	SIG
OA	WH2014	snp16590	4	5373534	11.66	1.72	0.49	1.39	2.72	2.20E-12	SIG
OA	WH2014	snp25032	5	38490643	4.80	-1.08	0.62	1.09	2.15	1.57E-05	SUG
OA	WH2014	snp25633	6	1436603	12.61	1.88	-0.63	3.26	6.40	2.45E-13	SIG
OA	WH2014	snp30327	6	43952755	6.75	-1.31	-0.37	1.47	2.88	1.80E-07	SIG
OA	WH2014	snp34838	7	25467893	6.26	-1.08	1.69	1.13	2.22	5.44E-07	SIG
OA	WH2014	snp51402	10	42975806	6.55	0.43	-4.76	1.48	2.92	2.80E-07	SIG
OA	WH2014	snp63683	13	21999755	7.87	1.35	-1.59	1.78	3.50	1.36E-08	SIG
OA	WH2014	snp67654	14	2005374	7.42	-1.23	-2.63	1.31	2.58	3.77E-08	SIG
OA	WH2014	snp71709	14	44994932	3.69	0.90	1.31	0.79	1.56	2.05E-04	SUG
OA	WH2014	snp78237	15	48590094	6.38	0.50	17.66	1.25	2.46	4.19E-07	SIG
OA	WH2014	snp82033	16	27822655	9.40	-0.39	-2.28	0.95	1.87	3.98E-10	SIG
OA	WH2014	snp89451	18	10496802	13.19	1.81	1.77	0.74	1.46	6.41E-14	SIG
OA	WH2014	snp89470	18	10590866	4.49	1.05	-0.99	0.98	1.92	3.22E-05	SUG
OA	WH2014	snp103925	20	33702042	10.26	1.62	0.48	1.34	2.62	5.50E-11	SIG
OA	WH2014	snp104226	20	35265033	4.50	0.13	6.02	0.95	1.87	3.14E-05	SUG
OA	WH2015	snp8885	2	37728774	40.01	3.03	-0.23	2.43	7.85	9.87E-41	SIG
OA	WH2015	snp15680	3	46445726	9.88	-1.32	0.32	1.14	3.68	1.32E-10	SIG
OA	WH2015	snp25032	5	38490643	12.42	-1.49	-0.76	2.04	6.60	3.79E-13	SIG
OA	WH2015	snp25637	6	1452457	5.60	0.91	-1.30	0.89	2.87	2.53E-06	SUG
OA	WH2015	snp27810	6	15772859	7.01	0.07	4.36	1.13	3.65	9.78E-08	SIG
OA	WH2015	snp30327	6	43952755	4.47	-0.88	0.06	0.66	2.13	3.41E-05	SUG
OA	WH2015	snp51619	10	44672512	7.70	1.15	-0.11	0.92	2.97	2.00E-08	SIG
OA	WH2015	snp52053	10	47257578	9.28	1.26	0.85	1.36	4.39	5.31E-10	SIG
OA	WH2015	snp57870	12	6128990	6.61	1.08	0.00	1.06	3.42	2.48E-07	SIG
OA	WH2015	snp82587	16	30485788	7.22	0.38	-4.18	1.13	3.64	5.97E-08	SIG
OA	WH2015	snp96763	19	8799955	7.03	-0.53	6.81	1.02	3.28	9.43E-08	SIG
OA	BLUP	snp9477	2	42382374	8.80	0.63	0.20	0.31	2.06	1.59E-09	SIG
OA	BLUP	snp15838	4	75684	10.19	0.66	0.16	0.25	1.68	6.53E-11	SIG
OA	BLUP	snp19595	4	39190702	69.99	-2.20	-2.85	0.41	2.73	1.03E-70	SIG
OA	BLUP	snp25215	5	39902352	8.32	0.60	0.17	0.33	2.23	4.79E-09	SIG
OA	BLUP	snp25629	6	1351463	4.70	0.41	0.60	0.17	1.15	1.99E-05	SUG
OA	BLUP	snp29577	6	38049083	4.30	0.22	-0.59	0.16	1.04	5.01E-05	SUG
OA	BLUP	snp30327	6	43952755	17.09	-0.88	-0.50	0.65	4.39	8.12E-18	SIG
OA	BLUP	snp39472	8	18244147	4.98	-0.39	1.04	0.19	1.26	1.04E-05	SUG
OA	BLUP	snp42769	9	1029823	8.08	0.48	-1.46	0.26	1.78	8.25E-09	SIG
OA	BLUP	snp48047	10	3539911	33.78	1.21	3.26	0.35	2.36	1.66E-34	SIG
OA	BLUP	snp55734	11	29913995	3.38	0.11	2.18	0.11	0.75	4.14E-04	SUG
OA	BLUP	snp57860	12	6039145	16.38	0.77	-2.08	0.59	3.98	4.21E-17	SIG
OA	BLUP	snp63683	13	21999755	9.14	0.62	-0.39	0.34	2.25	7.18E-10	SIG
OA	BLUP	snp65615	13	33136412	8.83	-0.43	-2.50	0.20	1.37	1.47E-09	SIG

OA	BLUP	snp78780	16	735028	10.09	0.61	-1.01	0.38	2.57	8.05E-11	SIG
OA	BLUP	snp79831	16	6824526	8.14	0.17	2.54	0.27	1.80	7.21E-09	SIG
OA	BLUP	snp82676	16	31027487	4.35	-0.36	-0.79	0.15	1.00	4.50E-05	SUG
OA	BLUP	snp84696	17	8069391	7.91	-0.54	0.97	0.21	1.42	1.23E-08	SIG
OA	BLUP	snp95545	18	62146771	6.96	0.52	0.67	0.22	1.51	1.10E-07	SIG
OA	BLUP	snp99133	19	39053992	67.94	2.17	-0.82	0.51	3.42	1.17E-68	SIG
OA	BLUP	snp100081	19	45688463	7.80	-0.32	1.17	0.33	2.18	1.59E-08	SIG
LA	NJ2011	snp9477	2	42382374	5.70	-0.69	0.40	0.40	2.39	1.99E-06	SUG
LA	NJ2011	snp16028	4	1524786	12.30	1.01	-0.57	0.98	5.84	4.99E-13	SIG
LA	NJ2011	snp25032	5	38490643	14.46	1.07	1.16	1.11	6.63	3.51E-15	SIG
LA	NJ2011	snp25555	6	751417	4.41	-0.58	0.39	0.28	1.65	3.91E-05	SUG
LA	NJ2011	snp27810	6	15772859	10.02	0.09	-3.46	0.78	4.69	9.51E-11	SIG
LA	NJ2011	snp53468	11	6225981	7.80	-0.79	-0.13	0.39	2.31	1.58E-08	SIG
LA	NJ2011	snp57257	12	2387406	11.85	1.00	0.01	0.71	4.26	1.42E-12	SIG
LA	NJ2011	snp68010	14	4251041	12.18	1.01	0.20	0.54	3.24	6.62E-13	SIG
LA	NJ2011	snp73422	15	6013922	20.48	1.32	1.34	0.42	2.49	3.33E-21	SIG
LA	NJ2011	snp82171	16	28447572	6.62	0.75	-0.15	0.53	3.18	2.38E-07	SIG
LA	NJ2011	snp86022	17	18642208	10.27	0.73	-8.53	0.32	1.91	5.35E-11	SIG
LA	NJ2011	snp88702	18	4812515	5.63	0.67	-0.19	0.38	2.24	2.34E-06	SUG
LA	NJ2011	snp88748	18	5130475	7.51	0.27	-4.47	0.59	3.52	3.12E-08	SIG
LA	NJ2011	snp94377	18	57090843	7.47	-0.79	-0.54	0.60	3.61	3.38E-08	SIG
LA	NJ2011	snp97740	19	27409100	12.30	-0.48	-1.17	0.36	2.18	5.02E-13	SIG
LA	NJ2011	snp100070	19	45536111	7.41	0.64	1.58	0.53	3.20	3.93E-08	SIG
LA	NJ2011	snp104263	20	35431550	6.41	-0.29	2.84	0.47	2.83	3.93E-07	SIG
LA	NJ2012	snp7011	2	11259810	8.84	-0.67	0.48	0.23	2.65	1.43E-09	SIG
LA	NJ2012	snp13680	3	32378949	13.69	0.86	0.36	0.63	7.16	2.04E-14	SIG
LA	NJ2012	snp14779	3	39486850	11.10	0.74	0.31	0.17	1.96	7.95E-12	SIG
LA	NJ2012	snp14796	3	39554541	9.37	-0.68	-0.46	0.45	5.04	4.26E-10	SIG
LA	NJ2012	snp16830	4	6717632	10.31	-0.70	-0.79	0.23	2.64	4.85E-11	SIG
LA	NJ2012	snp19240	4	36641266	26.74	-1.23	0.76	0.24	2.68	1.85E-27	SIG
LA	NJ2012	snp19476	4	38210946	8.65	0.65	0.32	0.25	2.88	2.25E-09	SIG
LA	NJ2012	snp25032	5	38490643	10.66	0.72	0.71	0.49	5.57	2.20E-11	SIG
LA	NJ2012	snp31476	7	836451	4.20	-0.24	1.95	0.19	2.17	6.27E-05	SUG
LA	NJ2012	snp40574	8	31058218	8.46	-1.21	-0.12	0.28	3.20	3.50E-09	SIG
LA	NJ2012	snp40726	8	34172036	16.73	-0.93	-0.12	0.18	2.02	1.85E-17	SIG
LA	NJ2012	snp51534	10	44061217	16.55	-0.92	0.09	0.21	2.32	2.83E-17	SIG
LA	NJ2012	snp51973	10	46679113	8.19	0.62	-1.35	0.22	2.54	6.42E-09	SIG
LA	NJ2012	snp53301	11	5071119	21.57	-1.08	0.85	0.26	2.96	2.69E-22	SIG
LA	NJ2012	snp57872	12	6138661	7.56	-0.61	0.50	0.26	2.92	2.76E-08	SIG
LA	NJ2012	snp81373	16	24196224	14.26	0.86	-0.78	0.44	4.97	5.50E-15	SIG
LA	NJ2014	snp22226	5	8031324	6.88	-0.83	0.39	0.30	2.31	1.32E-07	SIG
LA	NJ2014	snp25646	6	1499357	8.38	0.94	-0.52	0.64	4.99	4.17E-09	SIG
LA	NJ2014	snp30327	6	43952755	6.83	0.82	-0.74	0.63	4.90	1.48E-07	SIG
LA	NJ2014	snp51348	10	42589120	8.07	0.07	3.33	0.73	5.66	8.44E-09	SIG

LA	NJ2014	snp63683	13	21999755	4.80	-0.70	-0.07	0.40	3.11	1.57E-05	SUG
LA	NJ2014	snp74391	15	12373841	8.89	-0.92	-2.20	0.35	2.74	1.29E-09	SIG
LA	NJ2014	snp78940	16	1823964	7.41	-0.86	0.83	0.51	3.98	3.90E-08	SIG
LA	NJ2014	snp81975	16	27654752	8.45	-0.94		0.43	3.35	4.42E-10	SIG
LA	NJ2014	snp82685	16	31087981	7.21	0.88	-0.69	0.75	5.86	6.14E-08	SIG
LA	NJ2014	snp86732	17	32745157	6.44	0.84	0.12	0.49	3.79	3.65E-07	SIG
LA	NJ2014	snp91792	18	37825762	6.17	0.01	-1.54	0.48	3.70	6.71E-07	SIG
LA	NJ2015	snp15965	4	1022737	5.31	-0.59	0.44	0.20	1.89	4.94E-06	SUG
LA	NJ2015	snp16575	4	5246376	8.68	0.52	-4.60	0.41	3.86	2.11E-09	SIG
LA	NJ2015	snp25032	5	38490643	10.42	0.85	-0.22	0.67	6.38	3.81E-11	SIG
LA	NJ2015	snp30327	6	43952755	4.33	0.46	-0.98	0.27	2.57	4.66E-05	SUG
LA	NJ2015	snp42889	9	1879551	9.68	-0.81	0.31	0.42	3.96	2.09E-10	SIG
LA	NJ2015	snp46758	9	41114554	7.64	-0.65	1.22	0.47	4.45	2.29E-08	SIG
LA	NJ2015	snp51219	10	41503251	4.86	-1.38	0.07	0.30	2.86	1.39E-05	SUG
LA	NJ2015	snp57221	12	2065602	6.49	0.14	3.74	0.36	3.44	3.21E-07	SIG
LA	NJ2015	snp82759	16	31456994	5.81	0.63	0.00	0.33	3.13	1.56E-06	SUG
LA	NJ2015	snp88793	18	5426661	26.12	-1.42	-0.34	0.29	2.76	7.54E-27	SIG
LA	NJ2015	snp94113	18	55874196	3.74	-0.48		0.23	2.22	3.29E-05	SUG
LA	NJ2015	snp102846	20	23724879	52.61	2.30	4.33	0.25	2.40	2.47E-53	SIG
LA	NJ2016	snp10187	2	47294286	6.23	0.78	-0.16	0.32	1.95	5.93E-07	SIG
LA	NJ2016	snp15569	3	45409871	7.49	-0.14	-4.98	0.66	3.97	3.24E-08	SIG
LA	NJ2016	snp15838	4	75684	9.07	-0.96	0.01	0.55	3.31	8.52E-10	SIG
LA	NJ2016	snp29580	6	38054550	6.15	-0.08	1.43	0.47	2.85	7.15E-07	SIG
LA	NJ2016	snp34353	7	17960593	6.65	-0.10	-4.43	0.61	3.66	2.26E-07	SIG
LA	NJ2016	snp35897	7	38879620	15.08	-1.25	0.15	0.41	2.44	8.35E-16	SIG
LA	NJ2016	snp37099	8	2769635	7.92	0.86	-1.71	0.43	2.61	1.20E-08	SIG
LA	NJ2016	snp42897	9	1931670	11.02	-1.06	-1.05	0.33	1.98	9.66E-12	SIG
LA	NJ2016	snp48194	10	4490676	18.20	1.39	1.47	0.66	3.95	6.30E-19	SIG
LA	NJ2016	snp54597	11	15962795	7.08	0.48	4.63	0.54	3.25	8.27E-08	SIG
LA	NJ2016	snp55236	11	24194171	4.93	0.70	0.16	0.45	2.74	1.17E-05	SUG
LA	NJ2016	snp68010	14	4251041	10.15	1.01	0.85	0.52	3.16	7.05E-11	SIG
LA	NJ2016	snp72826	15	1932147	5.23	0.70	0.73	0.43	2.58	5.91E-06	SUG
LA	NJ2016	snp73422	15	6013922	17.66	1.36	1.23	0.44	2.63	2.17E-18	SIG
LA	NJ2016	snp78138	15	48099784	6.99	-0.82	-0.71	0.34	2.02	1.02E-07	SIG
LA	NJ2016	snp104475	20	36671087	5.04	0.64	1.36	0.45	2.73	9.18E-06	SUG
LA	WH2014	snp15838	4	75684	5.65	-1.03	-0.53	0.63	1.97	2.25E-06	SUG
LA	WH2014	snp16590	4	5373534	9.17	-1.30	1.72	0.98	3.07	6.69E-10	SIG
LA	WH2014	snp25032	5	38490643	7.36	1.20	0.18	1.32	4.14	4.33E-08	SIG
LA	WH2014	snp25633	6	1436603	8.97	-1.37	0.73	1.75	5.50	1.07E-09	SIG
LA	WH2014	snp34838	7	25467893	4.56	0.74	-1.44	0.70	2.20	2.73E-05	SUG
LA	WH2014	snp51402	10	42975806	8.67	-0.21	5.11	1.53	4.82	2.12E-09	SIG
LA	WH2014	snp63737	13	22330877	5.52	-1.05	-0.36	1.02	3.21	3.01E-06	SUG
LA	WH2014	snp71709	14	44994932	4.68	-0.89	-1.43	0.79	2.48	2.08E-05	SUG
LA	WH2014	snp89451	18	10496802	11.73	-1.51	0.94	0.71	2.24	1.88E-12	SIG

LA	WH2014	snp89470	18	10590866	12.43	-0.10	1.96	1.00	3.14	3.75E-13	SIG
LA	WH2014	snp97160	19	15500421	5.34	-0.27	-10.57	0.91	2.88	4.59E-06	SUG
LA	WH2014	snp103925	20	33702042	12.25	-1.54	1.99	1.48	4.67	5.64E-13	SIG
LA	WH2015	snp5752	2	2642931	7.23	0.87	0.88	0.59	2.74	5.85E-08	SIG
LA	WH2015	snp15680	3	46445726	6.38	0.83	0.23	0.44	2.05	4.17E-07	SIG
LA	WH2015	snp25032	5	38490643	20.71	1.58	-1.07	2.39	11.11	1.94E-21	SIG
LA	WH2015	snp28562	6	20639509	6.54	-0.12	-9.40	0.66	3.07	2.90E-07	SIG
LA	WH2015	snp30327	6	43952755	4.13	0.66	-0.38	0.40	1.84	7.47E-05	SUG
LA	WH2015	snp32381	7	6113541	6.03	-0.04	-4.07	0.62	2.90	9.40E-07	SUG
LA	WH2015	snp50691	10	38063911	11.34	1.12	1.14	0.94	4.38	4.56E-12	SIG
LA	WH2015	snp51619	10	44672512	7.59	-0.90	-0.60	0.55	2.54	2.60E-08	SIG
LA	WH2015	snp82191	16	28611203	10.76	-0.12	4.01	1.08	5.00	1.74E-11	SIG
LA	WH2015	snp84683	17	7976679	9.21	1.00	-0.19	0.39	1.80	6.23E-10	SIG
LA	WH2015	snp90968	18	22820455	7.94	0.77	-2.29	0.75	3.51	1.14E-08	SIG
LA	WH2015	snp91115	18	24528317	6.01	0.16	-3.99	0.65	3.03	9.74E-07	SUG
LA	WH2015	snp98935	19	37877576	9.84	1.06	0.90	0.73	3.39	1.44E-10	SIG
LA	BLUP	snp9477	2	42382374	6.13	-0.45	0.01	0.16	1.77	7.44E-07	SIG
LA	BLUP	snp15838	4	75684	4.23	-0.36	0.04	0.08	0.85	5.84E-05	SUG
LA	BLUP	snp19595	4	39190702	81.74	2.20	2.54	0.40	4.38	1.82E-82	SIG
LA	BLUP	snp25032	5	38490643	23.72	0.93	0.27	0.79	8.58	1.89E-24	SIG
LA	BLUP	snp25550	6	720481	9.24	-0.11	2.35	0.28	3.03	5.72E-10	SIG
LA	BLUP	snp25644	6	1490151	6.58	-0.47	0.01	0.19	2.11	2.61E-07	SIG
LA	BLUP	snp26290	6	5956754	5.10	0.38	0.45	0.11	1.20	7.92E-06	SUG
LA	BLUP	snp30327	6	43952755	8.95	0.54	-0.30	0.26	2.80	1.13E-09	SIG
LA	BLUP	snp31476	7	836451	6.83	-0.17	2.24	0.21	2.26	1.49E-07	SIG
LA	BLUP	snp57860	12	6039145	14.92	-0.71	0.39	0.35	3.84	1.21E-15	SIG
LA	BLUP	snp60725	12	35774274	12.37	-0.04	-3.31	0.37	3.96	4.24E-13	SIG
LA	BLUP	snp63683	13	21999755	9.95	-0.52	0.93	0.30	3.20	1.13E-10	SIG
LA	BLUP	snp78780	16	735028	10.79	-0.58	-0.62	0.27	2.96	1.61E-11	SIG
LA	BLUP	snp79831	16	6824526	10.59	0.00	-2.68	0.31	3.41	2.55E-11	SIG
LA	BLUP	snp84696	17	8069391	6.14	0.43	-0.45	0.11	1.19	7.21E-07	SIG
LA	BLUP	snp95545	18	62146771	8.15	-0.51	-0.13	0.21	2.27	7.16E-09	SIG
LA	BLUP	snp100593	19	49513282	18.61	0.77	1.98	0.22	2.34	2.45E-19	SIG
LNA	NJ2011	snp8455	2	32958811	58.38	-1.10	1.05	0.22	7.83	4.19E-59	SIG
LNA	NJ2011	snp23219	5	23428593	41.23	-0.84	-0.78	0.11	3.90	5.92E-42	SIG
LNA	NJ2011	snp24121	5	31329679	5.15	-0.17	3.14	0.04	1.46	7.09E-06	SUG
LNA	NJ2011	snp44006	9	9605129	4.46	0.21	0.41	0.05	1.90	3.45E-05	SUG
LNA	NJ2011	snp65514	13	32535792	4.67	-0.24		0.05	1.67	3.56E-06	SUG
LNA	NJ2011	snp69645	14	18242578	10.15	-0.34	1.01	0.04	1.28	7.12E-11	SIG
LNA	NJ2011	snp69931	14	22294811	13.25	-0.44	0.21	0.02	0.87	5.62E-14	SIG
LNA	NJ2011	snp72513	14	49492467	7.78	-0.32	-0.17	0.09	3.10	1.67E-08	SIG
LNA	NJ2011	snp74217	15	11308133	14.03	-0.45		0.04	1.42	9.12E-16	SIG
LNA	NJ2011	snp82039	16	27893117	4.86	0.26	-0.23	0.07	2.38	1.37E-05	SUG
LNA	NJ2011	snp87557	17	39024091	10.97	-0.39	-0.42	0.06	2.20	1.08E-11	SIG

LNA	NJ2011	snp95117	18	60388330	30.37	-0.69		0.06	2.15	2.84E-32	SIG
LNA	NJ2012	snp40839	8	35164191	28.60	-0.65	-0.64	0.12	4.86	2.50E-29	SIG
LNA	NJ2012	snp42530	8	46480366	26.71	-0.64	1.04	0.05	1.96	1.97E-27	SIG
LNA	NJ2012	snp48557	10	7706982	30.79	-0.68	-1.75	0.04	1.67	1.63E-31	SIG
LNA	NJ2012	snp74273	15	11765119	19.89	-0.53	0.43	0.12	4.85	1.30E-20	SIG
LNA	NJ2012	snp81558	16	25313553	9.37	-0.35	0.10	0.07	2.81	4.27E-10	SIG
LNA	NJ2012	snp82374	16	29588805	29.28	-0.65	-0.82	0.10	4.31	5.30E-30	SIG
LNA	NJ2012	snp86671	17	32163219	16.47	-0.48		0.03	1.10	3.09E-18	SIG
LNA	NJ2012	snp86912	17	34522807	32.34	-0.65	4.71	0.13	5.26	4.58E-33	SIG
LNA	NJ2012	snp94336	18	56824133	4.93	-0.24	-0.34	0.04	1.80	1.18E-05	SUG
LNA	NJ2014	snp2529	1	30567509	8.52	-0.16	1.30	0.08	3.90	3.02E-09	SIG
LNA	NJ2014	snp13949	3	34407125	46.50	-0.80	-0.33	0.10	5.11	3.14E-47	SIG
LNA	NJ2014	snp21165	5	206343	67.66	-1.07	0.94	0.14	7.30	2.19E-68	SIG
LNA	NJ2014	snp40835	8	35126453	15.87	-0.41	0.28	0.04	2.25	1.36E-16	SIG
LNA	NJ2014	snp41168	8	38682870	9.53	0.10	-1.98	0.09	4.69	2.95E-10	SIG
LNA	NJ2014	snp53703	11	7771599	5.44	-0.05	-0.84	0.05	2.47	3.63E-06	SUG
LNA	NJ2014	snp73103	15	3855027	21.14	-0.47	0.70	0.07	3.54	7.34E-22	SIG
LNA	NJ2014	snp81455	16	24683066	10.88	-0.35	0.16	0.10	5.41	1.32E-11	SIG
LNA	NJ2014	snp93816	18	54770974	30.92	-0.60	-0.51	0.03	1.55	1.20E-31	SIG
LNA	NJ2014	snp96712	19	8457219	16.24	-0.40	-1.17	0.03	1.35	5.79E-17	SIG
LNA	NJ2015	snp386	1	2647246	30.38	-0.70	-0.56	0.05	1.98	4.14E-31	SIG
LNA	NJ2015	snp4487	1	49157127	14.54	-0.45	0.49	0.05	2.06	2.87E-15	SIG
LNA	NJ2015	snp8536	2	33701230	20.41	-0.56	-0.27	0.05	1.89	3.90E-21	SIG
LNA	NJ2015	snp9197	2	40328031	29.51	-0.72	0.48	0.04	1.50	3.07E-30	SIG
LNA	NJ2015	snp37662	8	6810236	21.09	-0.57	-0.54	0.08	3.41	8.23E-22	SIG
LNA	NJ2015	snp53164	11	4257613	23.74	-0.58	-1.09	0.07	2.96	1.84E-24	SIG
LNA	NJ2015	snp71898	14	46078679	27.49	-0.63	2.97	0.13	5.51	3.25E-28	SIG
LNA	NJ2016	snp668	1	4450294	4.81	-0.24	0.17	0.04	1.19	1.56E-05	SUG
LNA	NJ2016	snp6706	2	9225070	3.89	0.21		0.04	1.12	2.33E-05	SUG
LNA	NJ2016	snp38281	8	10618759	7.69	0.06	0.63	0.06	1.70	2.05E-08	SIG
LNA	NJ2016	snp38903	8	14811585	4.36	-0.23	0.12	0.05	1.45	4.37E-05	SUG
LNA	NJ2016	snp39805	8	20452423	15.71	-0.46	-0.53	0.07	2.22	1.97E-16	SIG
LNA	NJ2016	snp43158	9	3532214	6.58	-0.27	0.41	0.05	1.66	2.64E-07	SIG
LNA	NJ2016	snp47504	9	46335989	32.15	-0.69	-0.69	0.06	1.86	7.06E-33	SIG
LNA	NJ2016	snp58037	12	7385714	32.06	-0.71	-0.11	0.07	2.18	8.70E-33	SIG
LNA	NJ2016	snp64304	13	26152178	23.17	-0.56	0.15	0.07	2.11	6.74E-24	SIG
LNA	NJ2016	snp71094	14	39547704	19.13	0.44	1.48	0.04	1.17	7.46E-20	SIG
LNA	NJ2016	snp81338	16	23985700	48.72	-0.92	-0.57	0.10	2.93	1.91E-49	SIG
LNA	NJ2016	snp86712	17	32509252	16.16	-0.45		0.04	1.28	6.32E-18	SIG
LNA	NJ2016	snp91455	18	30296328	53.38	-0.96	-1.41	0.11	3.47	4.16E-54	SIG
LNA	NJ2016	snp92606	18	48368243	18.11	-0.48	-0.44	0.08	2.58	7.81E-19	SIG
LNA	NJ2016	snp104642	20	37616538	6.85	0.30	0.02	0.08	2.49	1.41E-07	SIG
LNA	WH2014	snp4206	1	47482360	24.70	-0.57	-0.35	0.03	1.47	2.01E-25	SIG
LNA	WH2014	snp13949	3	34407125	41.47	-0.75	-0.04	0.07	3.48	3.38E-42	SIG

LNA	WH2014	snp25099	5	38972557	5.15	-0.22	-0.13	0.03	1.80	7.16E-06	SUG
LNA	WH2014	snp25587	6	961556	25.72	-0.54	-0.81	0.07	3.47	1.91E-26	SIG
LNA	WH2014	snp41426	8	40563528	8.94	0.20	-1.57	0.07	3.93	1.14E-09	SIG
LNA	WH2014	snp55248	11	24326740	16.40	-0.41	0.97	0.03	1.83	4.00E-17	SIG
LNA	WH2014	snp62879	13	11652041	11.93	-0.34	-0.73	0.02	1.18	1.17E-12	SIG
LNA	WH2014	snp73103	15	3855027	44.81	-0.79	-0.08	0.11	5.66	1.57E-45	SIG
LNA	WH2014	snp92716	18	48985284	22.93	-0.50	1.23	0.06	3.08	1.17E-23	SIG
LNA	WH2014	snp94361	18	56993597	19.68	-0.47	-0.70	0.05	2.51	2.08E-20	SIG
LNA	WH2014	snp94705	18	58830263	3.94	-0.20	0.11	0.04	1.96	1.14E-04	SUG
LNA	WH2015	snp14751	3	39308521	20.18	-0.46	0.02	0.05	2.01	6.58E-21	SIG
LNA	WH2015	snp18698	4	27935787	20.11	0.37	2.34	0.05	2.41	7.85E-21	SIG
LNA	WH2015	snp22010	5	5638135	58.00	-0.93	-1.05	0.05	2.44	1.00E-58	SIG
LNA	WH2015	snp30334	6	43979045	5.80	-0.20	-0.51	0.05	2.22	1.60E-06	SUG
LNA	WH2015	snp33770	7	14354830	7.24	-0.26	0.07	0.06	2.63	5.79E-08	SIG
LNA	WH2015	snp40881	8	35577366	15.04	-0.34	2.89	0.06	2.61	9.18E-16	SIG
LNA	WH2015	snp58037	12	7385714	36.24	-0.66	1.55	0.10	4.64	5.71E-37	SIG
LNA	WH2015	snp79857	16	6986880	10.05	-0.29	-0.90	0.02	1.01	8.91E-11	SIG
LNA	WH2015	snp82587	16	30485788	7.43	-0.11	0.95	0.06	2.82	3.73E-08	SIG
LNA	WH2015	snp88723	18	5041385	7.02	-0.26	-0.01	0.03	1.33	9.57E-08	SIG
LNA	WH2015	snp92993	18	50974424	21.95	-0.48	-0.13	0.03	1.45	1.14E-22	SIG
LNA	WH2015	snp95857	19	1780843	7.55	-0.26	-0.13	0.06	2.68	2.80E-08	SIG
LNA	BLUP	snp33232	7	10138673	7.18	-0.23	0.34	0.03	1.57	6.62E-08	SIG
LNA	BLUP	snp40839	8	35164191	17.23	-0.37	-0.43	0.04	2.10	5.91E-18	SIG
LNA	BLUP	snp55271	11	24443070	41.63	-0.59	-1.95	0.07	4.08	2.33E-42	SIG
LNA	BLUP	snp58037	12	7385714	36.05	-0.59	1.09	0.08	4.25	8.97E-37	SIG
LNA	BLUP	snp70677	14	31140033	38.23	-0.58	1.13	0.09	4.79	5.89E-39	SIG
LNA	BLUP	snp73103	15	3855027	28.83	-0.50	-0.52	0.05	2.58	1.47E-29	SIG
LNA	BLUP	snp86592	17	31191973	36.17	-0.58	0.24	0.04	2.27	6.77E-37	SIG
LNA	BLUP	snp92589	18	48276099	18.57	-0.40	-0.07	0.07	3.63	2.67E-19	SIG
OIL	NJ2014	snp7267	2	13462826	14.34	0.79	-0.82	0.29	6.05	4.62E-15	SIG
OIL	NJ2014	snp25031	5	38490635	10.60	0.67	0.60	0.43	8.94	2.52E-11	SIG
OIL	NJ2014	snp51383	10	42855511	13.86	0.72	-1.61	0.32	6.75	1.39E-14	SIG
OIL	NJ2014	snp56606	11	37035535	11.87	-0.70	1.12	0.28	5.88	1.35E-12	SIG
OIL	NJ2014	snp65191	13	30634520	30.54	-1.23	0.04	0.22	4.66	2.88E-31	SIG
OIL	NJ2014	snp92527	18	47740890	51.47	1.77	1.75	0.44	9.27	3.43E-52	SIG
OIL	NJ2015	snp17323	4	9983937	12.23	0.69	1.12	0.33	5.87	5.96E-13	SIG
OIL	NJ2015	snp28784	6	22461135	33.34	-1.31	-0.60	0.29	5.14	4.61E-34	SIG
OIL	NJ2015	snp30227	6	43062373	5.56	-0.04	1.86	0.21	3.75	2.77E-06	SUG
OIL	NJ2015	snp35607	7	36747805	6.93	-0.53	-0.45	0.27	4.73	1.16E-07	SIG
OIL	NJ2015	snp36511	7	43205271	5.76	0.48	0.49	0.16	2.76	1.74E-06	SUG
OIL	NJ2015	snp47775	10	1654017	9.62	0.63	-0.21	0.11	2.03	2.40E-10	SIG
OIL	NJ2015	snp87644	17	39673242	7.52	0.55	-0.90	0.17	3.01	3.01E-08	SIG
OIL	NJ2015	snp92606	18	48368243	4.12	0.26	-1.39	0.14	2.56	7.62E-05	SUG
OIL	NJ2015	snp94673	18	58724929	5.29	0.43	-1.09	0.12	2.20	5.18E-06	SUG

OIL	NJ2015	snp98736	19	36713181	4.22	-0.42	0.22	0.12	2.18	6.02E-05	SUG
OIL	NJ2015	snp103484	20	30573160	13.54	0.80	0.06	0.16	2.89	2.88E-14	SIG
OIL	NJ2016	snp9460	2	42249678	40.38	1.28	-0.14	0.21	5.75	4.16E-41	SIG
OIL	NJ2016	snp25031	5	38490635	4.88	0.39	-0.06	0.14	3.79	1.32E-05	SUG
OIL	NJ2016	snp32265	7	5441000	17.20	-0.77	-0.80	0.16	4.33	6.30E-18	SIG
OIL	NJ2016	snp41118	8	38282260	4.32	-0.01	-2.23	0.12	3.27	4.82E-05	SUG
OIL	NJ2016	snp50956	10	39787680	9.95	-0.56	0.42	0.17	4.70	1.12E-10	SIG
OIL	NJ2016	snp63233	13	14402337	7.95	-0.50	-0.21	0.15	4.05	1.13E-08	SIG
OIL	NJ2016	snp88395	18	2635738	5.58	0.04	1.54	0.15	3.99	2.65E-06	SUG
OIL	NJ2016	snp94475	18	57687310	5.79	0.42	0.26	0.08	2.13	1.64E-06	SUG
OIL	NJ2016	snp103917	20	33662034	15.81	-0.72	-0.11	0.36	9.66	1.56E-16	SIG
OIL	WH2014	snp7349	2	14128122	6.29	0.45	-0.17	0.13	3.09	5.17E-07	SIG
OIL	WH2014	snp8050	2	27843353	4.97	0.33	-1.29	0.10	2.34	1.08E-05	SUG
OIL	WH2014	snp23758	5	28271870	6.16	-0.51	0.30	0.17	3.92	6.99E-07	SIG
OIL	WH2014	snp23785	5	28483791	19.46	0.83	-0.40	0.13	3.00	3.44E-20	SIG
OIL	WH2014	snp25040	5	38574145	6.11	0.43	0.26	0.18	4.10	7.84E-07	SIG
OIL	WH2014	snp28055	6	17187723	11.46	0.61	-0.13	0.16	3.63	3.50E-12	SIG
OIL	WH2014	snp32265	7	5441000	12.82	-0.66	1.06	0.14	3.20	1.52E-13	SIG
OIL	WH2014	snp41959	8	42971491	15.01	-0.68	-1.24	0.14	3.26	9.73E-16	SIG
OIL	WH2014	snp48066	10	3696055	7.36	-0.26	2.77	0.21	4.82	4.41E-08	SIG
OIL	WH2014	snp51917	10	46344586	15.15	0.72	0.69	0.19	4.39	7.01E-16	SIG
OIL	WH2014	snp73103	15	3855027	11.48	0.61	-0.19	0.07	1.61	3.34E-12	SIG
OIL	WH2014	snp90162	18	16296555	3.63	0.33	-0.15	0.10	2.39	2.37E-04	SUG
OIL	WH2014	snp104283	20	35564773	10.97	-0.59	-0.20	0.31	7.24	1.07E-11	SIG
OIL	WH2015	snp11738	3	5803486	11.31	-0.57	0.68	0.13	2.92	4.89E-12	SIG
OIL	WH2015	snp25032	5	38490643	9.57	0.52	0.21	0.25	5.63	2.68E-10	SIG
OIL	WH2015	snp31249	6	49813111	7.99	-0.47	-0.22	0.18	3.93	1.03E-08	SIG
OIL	WH2015	snp32056	7	4382383	10.24	0.54	0.27	0.18	4.01	5.77E-11	SIG
OIL	WH2015	snp32174	7	5040905	27.61	-0.89	-2.22	0.16	3.49	2.47E-28	SIG
OIL	WH2015	snp43431	9	5442208	10.16	-0.55	-0.05	0.12	2.78	6.87E-11	SIG
OIL	WH2015	snp48979	10	12460090	10.39	0.55	-0.89	0.09	2.05	4.09E-11	SIG
OIL	WH2015	snp52368	10	49564516	3.55	0.31	0.19	0.09	2.03	2.81E-04	SUG
OIL	WH2015	snp73499	15	6481297	4.72	0.35		0.10	2.26	3.15E-06	SUG
OIL	WH2015	snp77623	15	44193203	6.03	-0.59	0.24	0.17	3.89	9.38E-07	SUG
OIL	WH2015	snp78979	16	1925475	5.53	0.22	-1.74	0.15	3.45	2.94E-06	SUG
OIL	WH2015	snp80991	16	19265833	12.32	0.57	0.88	0.30	6.73	4.85E-13	SIG
OIL	WH2015	snp95779	19	1291229	5.59	0.41	0.01	0.15	3.29	2.59E-06	SUG
OIL	WH2015	snp104284	20	35588276	8.11	-0.04	-3.40	0.21	4.62	7.85E-09	SIG
OIL	BLUP	snp25031	5	38490635	14.21	0.43	0.13	0.17	8.02	6.15E-15	SIG
OIL	BLUP	snp32265	7	5441000	11.69	-0.39	0.06	0.04	2.01	2.06E-12	SIG
OIL	BLUP	snp36613	7	43947021	3.21	0.19	-0.13	0.03	1.49	6.19E-04	SUG
OIL	BLUP	snp39185	8	16500872	6.36	0.26	0.34	0.02	1.05	4.34E-07	SIG
OIL	BLUP	snp55248	11	24326740	23.73	0.56	-0.46	0.07	3.39	1.86E-24	SIG
OIL	BLUP	snp56606	11	37035535	7.64	-0.28	0.73	0.06	2.65	2.31E-08	SIG

OIL	BLUP	snp71629	14	44495900	4.58	0.05	0.87	0.05	2.21	2.64E-05	SUG
OIL	BLUP	snp85112	17	11381352	3.23	-0.19	-0.25	0.03	1.60	5.88E-04	SUG
OIL	BLUP	snp88787	18	5359420	11.45	0.37	-0.01	0.09	4.11	3.54E-12	SIG
OIL	BLUP	snp90162	18	16296555	3.39	0.19	-0.23	0.04	1.75	4.04E-04	SUG
OIL	BLUP	snp95858	19	1780893	11.78	0.38	-0.19	0.13	6.13	1.68E-12	SIG
OIL	BLUP	snp103489	20	30581992	14.24	0.41	0.23	0.04	1.86	5.78E-15	SIG
OIL	BLUP	snp104284	20	35588276	6.32	0.14	-1.69	0.06	2.90	4.84E-07	SIG

PA: palmitic acid; SA: stearic acid; OA: oleic acid; LA: linoleic acid; LNA: linolenic acid; OIL: oil content; Env: environment; NJ2011, NJ2012, NJ2014, NJ2015 and NJ2016: Jiangpu experimental station of Nanjing Agricultural University in 2011, 2012, 2014, 2015 and 2016, respectively; WH2014 and WH2015: Wuhan experimental stations of Huazhong Agricultural University in 2014 and 2015, respectively; BLUP: best linear unbiased prediction; Chr: chromosome; Pos: position; Add: additive; Dom: dominance; Var: variance; SIG: significant (-log10(P) ≥ 6.04); SUG: suggestion (LOD ≥ 3).

Table S6. QTNs for soybean seed oil-related traits in all the environments using 3VmrMLM

Trait	Marker	Chr	Pos (bp)	LOD (Q)	Add	Dom	Var	r^2 (%)	P-value	Significance
PA	snp6550	2	8020378	6.94	0.08	-0.04	0.01	0.45	1.15E-07	SIG
PA	snp10230	2	47545567	30.88	-0.16	-0.49	0.01	0.88	1.32E-31	SIG
PA	snp12518	3	18821058	38.07	-0.20	0.05	0.01	0.99	8.45E-39	SIG
PA	snp13862	3	33766980	6.48	0.08	0.00	0.01	0.37	3.28E-07	SIG
PA	snp16420	4	4161316	8.78	-0.10	-0.04	0.01	0.66	1.67E-09	SIG
PA	snp21227	5	622923	9.57	-0.10	0.00	0.01	0.64	2.68E-10	SIG
PA	snp21981	5	5401577	19.53	-0.14	0.04	0.01	0.36	2.96E-20	SIG
PA	snp22231	5	8057446	68.17	0.28	-0.24	0.02	1.18	6.74E-69	SIG
PA	snp25804	6	2708047	3.42	-0.06	0.09	0.00	0.26	3.78E-04	SUG
PA	snp26308	6	6123748	6.78	0.08	0.05	0.01	0.43	1.67E-07	SIG
PA	snp32471	7	6463073	6.29	0.08	-0.05	0.01	0.45	5.14E-07	SIG
PA	snp34260	7	17318313	5.26	-0.07	0.06	0.01	0.37	5.44E-06	SUG
PA	snp44903	9	21153630	27.61	0.17	0.07	0.01	0.90	2.44E-28	SIG
PA	snp47385	9	45304115	16.54	0.13	-0.18	0.01	0.55	2.90E-17	SIG
PA	snp50420	10	35130459	6.44	-0.08	0.06	0.01	0.47	3.66E-07	SIG
PA	snp53737	11	8085669	15.93	0.13	-0.11	0.02	1.27	1.17E-16	SIG
PA	snp53788	11	8398437	7.06	0.08	0.08	0.01	0.49	8.72E-08	SIG
PA	snp60905	12	37076650	23.22	-0.14	-0.41	0.01	1.00	6.00E-24	SIG
PA	snp62193	13	6124251	11.82	0.11	0.17	0.01	0.51	1.52E-12	SIG
PA	snp63687	13	22045116	19.34	0.00	0.59	0.02	1.43	4.58E-20	SIG
PA	snp65526	13	32610399	74.03	0.28	0.33	0.01	1.02	9.41E-75	SIG
PA	snp66022	13	35687169	6.05	-0.07	0.19	0.01	0.39	8.85E-07	SIG
PA	snp67717	14	2549865	17.01	0.02	0.53	0.02	1.20	9.82E-18	SIG
PA	snp72115	14	47225911	30.15	-0.18	-0.09	0.01	0.41	7.15E-31	SIG
PA	snp72140	14	47366647	9.75	-0.04	-0.39	0.01	0.59	1.78E-10	SIG
PA	snp73350	15	5592008	79.50	-0.31	0.05	0.02	1.18	3.19E-80	SIG
PA	snp75103	15	16641263	6.62	0.08	0.07	0.01	0.48	2.38E-07	SIG
PA	snp78230	15	48569042	10.92	-0.11	0.00	0.01	0.49	1.19E-11	SIG
PA	snp79687	16	6022476	4.40	-0.06	-0.11	0.00	0.22	3.96E-05	SUG
PA	snp81943	16	27545905	7.59	-0.09	-0.09	0.01	0.50	2.58E-08	SIG
PA	snp83615	16	36773852	10.82	0.11	0.07	0.01	0.46	1.50E-11	SIG
PA	snp85069	17	11159168	40.62	-0.21	0.20	0.01	1.05	2.43E-41	SIG
PA	snp87865	17	41081736	6.06	0.00	0.54	0.01	0.43	8.76E-07	SIG
PA	snp90109	18	15919510	13.66	-0.11	-0.24	0.01	0.88	2.19E-14	SIG
PA	snp97698	19	27062925	30.12	0.18	-0.14	0.01	1.08	7.66E-31	SIG
PA	snp104335	20	35860893	8.90	0.00	0.35	0.01	0.60	1.26E-09	SIG
PA	snp104975	20	39923283	10.36	-0.01	-0.34	0.01	0.70	4.38E-11	SIG
SA	snp9451	2	42193331	10.08	0.03	0.13	0.00	0.53	8.28E-11	SIG
SA	snp16518	4	4924161	49.46	-0.09	0.05	0.00	1.73	3.48E-50	SIG
SA	snp18752	4	28666272	23.79	-0.06	-0.07	0.00	0.83	1.62E-24	SIG
SA	snp20383	4	44245421	22.90	0.05	-0.10	0.00	1.34	1.26E-23	SIG

SA	snp21288	5	1175202	17.30	-0.05	0.09	0.00	0.97	5.00E-18	SIG
SA	snp24600	5	35101815	18.91	-0.05	0.03	0.00	1.34	1.22E-19	SIG
SA	snp29030	6	28231300	12.50	-0.03	-0.18	0.00	0.68	3.14E-13	SIG
SA	snp30999	6	48106897	18.06	0.00	-0.18	0.00	1.16	8.81E-19	SIG
SA	snp38216	8	10265612	33.38	-0.06	0.26	0.00	1.37	4.22E-34	SIG
SA	snp42423	8	45772197	25.30	-0.06	0.09	0.00	0.72	4.98E-26	SIG
SA	snp43778	9	7603719	15.69	-0.05	0.03	0.00	0.70	2.05E-16	SIG
SA	snp45975	9	35606339	38.89	0.08	0.09	0.00	2.01	1.31E-39	SIG
SA	snp46717	9	40842602	11.63	0.04	-0.01	0.00	0.74	2.36E-12	SIG
SA	snp47001	9	42417831	5.61	-0.03	-0.06	0.00	0.18	2.44E-06	SUG
SA	snp48019	10	3334509	68.11	-0.10	-0.36	0.00	0.89	7.80E-69	SIG
SA	snp51590	10	44469282	12.29	-0.04	0.00	0.00	0.77	5.13E-13	SIG
SA	snp52150	10	47948265	6.94	0.03	0.07	0.00	0.42	1.15E-07	SIG
SA	snp55255	11	24371125	9.06	0.04	0.05	0.00	0.59	8.71E-10	SIG
SA	snp57864	12	6104790	47.91	-0.08	-0.20	0.01	3.34	1.23E-48	SIG
SA	snp58388	12	9608584	5.79	0.01	0.10	0.00	0.38	1.63E-06	SUG
SA	snp60495	12	34329030	8.83	0.04	-0.04	0.00	0.41	1.47E-09	SIG
SA	snp67886	14	3441497	20.70	-0.06	0.07	0.00	1.21	1.98E-21	SIG
SA	snp71149	14	40769103	41.00	-0.08	0.08	0.00	1.76	1.00E-41	SIG
SA	snp73686	15	7617269	11.13	-0.04	0.03	0.00	0.79	7.38E-12	SIG
SA	snp79529	16	5172417	23.27	-0.06	-0.07	0.00	0.42	5.42E-24	SIG
SA	snp82676	16	31027487	4.99	0.02	-0.07	0.00	0.35	1.01E-05	SUG
SA	snp82796	16	31568726	26.63	0.06	0.22	0.00	0.72	2.36E-27	SIG
SA	snp83221	16	34246889	4.23	0.03	0.01	0.00	0.26	5.87E-05	SUG
SA	snp83279	16	35261831	12.03	-0.04	0.07	0.00	0.92	9.42E-13	SIG
SA	snp84602	17	7451556	10.70	0.04	0.03	0.00	0.56	1.99E-11	SIG
SA	snp87869	17	41091544	13.91	-0.05	-0.05	0.00	0.57	1.23E-14	SIG
SA	snp88021	18	64350	19.36	-0.05	-0.06	0.00	0.71	4.38E-20	SIG
SA	snp89619	18	11853399	32.68	-0.01	0.27	0.00	2.23	2.08E-33	SIG
SA	snp92824	18	49852899	44.78	-0.08	-0.04	0.00	0.67	1.65E-45	SIG
SA	snp98399	19	34215110	21.31	-0.06	0.10	0.00	0.64	4.91E-22	SIG
SA	snp98797	19	37012487	10.57	-0.02	0.41	0.00	0.63	2.69E-11	SIG
SA	snp99919	19	44619057	8.57	0.04	-0.04	0.00	0.51	2.70E-09	SIG
SA	snp100911	20	852754	9.83	-0.02	-0.17	0.00	0.53	1.49E-10	SIG
OA	snp1331	1	10180892	13.22	-0.67	0.20	0.22	0.77	6.01E-14	SIG
OA	snp4406	1	48527274	11.18	0.03	-1.62	0.18	0.62	6.62E-12	SIG
OA	snp5982	2	4110866	33.55	0.77	0.47	0.10	0.35	2.83E-34	SIG
OA	snp9477	2	42382374	16.49	0.56	-0.29	0.26	0.91	3.25E-17	SIG
OA	snp10500	2	49370610	19.02	-0.27	-1.92	0.26	0.92	9.48E-20	SIG
OA	snp10627	2	50163292	10.92	0.29	1.04	0.17	0.59	1.21E-11	SIG
OA	snp11042	3	1344095	5.25	0.25	-0.70	0.08	0.29	5.57E-06	SUG
OA	snp15838	4	75684	18.73	0.58	-0.29	0.21	0.74	1.85E-19	SIG
OA	snp15896	4	446635	9.25	-0.19	2.27	0.15	0.53	5.69E-10	SIG
OA	snp20748	4	46845246	20.10	-0.42	4.80	0.20	0.72	7.91E-21	SIG

OA	snp21128	4	49187867	27.74	0.33	-5.93	0.41	1.43	1.80E-28	SIG
OA	snp22299	5	8446050	22.93	0.65	0.76	0.06	0.22	1.19E-23	SIG
OA	snp24202	5	31833925	9.49	-0.37	0.93	0.17	0.58	3.27E-10	SIG
OA	snp24342	5	32890704	19.32	-0.60	-0.08	0.33	1.14	4.84E-20	SIG
OA	snp25032	5	38490643	23.18	-0.65	0.13	0.39	1.36	6.65E-24	SIG
OA	snp25620	6	1260164	55.41	-1.00	-0.80	0.25	0.89	3.91E-56	SIG
OA	snp25644	6	1490151	12.93	0.41	1.09	0.20	0.71	1.18E-13	SIG
OA	snp30194	6	42857641	4.03	-0.28	0.01	0.05	0.17	9.40E-05	SUG
OA	snp31390	7	99598	27.30	0.69	0.80	0.18	0.62	4.99E-28	SIG
OA	snp32179	7	5054259	4.29	0.21	-1.67	0.04	0.15	5.08E-05	SUG
OA	snp32422	7	6246072	22.66	-0.58	-1.02	0.38	1.33	2.17E-23	SIG
OA	snp36568	7	43611859	13.29	0.59	-0.15	0.17	0.59	5.14E-14	SIG
OA	snp36954	8	1784895	6.07	-0.29	0.76	0.11	0.38	8.55E-07	SIG
OA	snp38127	8	9802623	10.21	0.42	0.63	0.18	0.63	6.12E-11	SIG
OA	snp39442	8	18087113	31.13	-0.74	1.71	0.08	0.29	7.39E-32	SIG
OA	snp41429	8	40580928	13.37	-0.07	4.49	0.22	0.79	4.32E-14	SIG
OA	snp42071	8	43518470	25.44	0.63	1.30	0.17	0.61	3.65E-26	SIG
OA	snp46056	9	36142660	30.59	-0.14	-2.99	0.48	1.68	2.56E-31	SIG
OA	snp51630	10	44741594	16.82	0.54	0.57	0.06	0.21	1.52E-17	SIG
OA	snp52053	10	47257578	15.70	0.53	0.18	0.24	0.83	2.01E-16	SIG
OA	snp52611	11	223519	11.26	-0.16	3.02	0.19	0.66	5.47E-12	SIG
OA	snp52680	11	612019	28.92	-0.72	-0.51	0.50	1.74	1.21E-29	SIG
OA	snp53589	11	7020279	37.70	0.82	-0.16	0.26	0.92	1.99E-38	SIG
OA	snp63124	13	13551174	11.87	-0.44	-0.92	0.08	0.27	1.35E-12	SIG
OA	snp63683	13	21999755	23.99	0.65	0.56	0.35	1.22	1.03E-24	SIG
OA	snp64502	13	27428372	9.92	-0.44	-0.06	0.18	0.64	1.22E-10	SIG
OA	snp68010	14	4251041	24.83	-0.67	-0.33	0.23	0.81	1.47E-25	SIG
OA	snp72924	15	2519632	15.21	-0.52	-0.42	0.26	0.91	6.15E-16	SIG
OA	snp84696	17	8069391	15.67	-0.48	1.03	0.19	0.65	2.13E-16	SIG
OA	snp100081	19	45688463	14.84	-0.08	1.21	0.23	0.82	1.43E-15	SIG
OA	snp103749	20	32532670	4.84	0.21	0.37	0.04	0.13	1.46E-05	SUG
LA	snp4406	1	48527274	8.22	-0.03	1.15	0.09	0.49	6.10E-09	SIG
LA	snp9477	2	42382374	21.67	-0.52	0.45	0.24	1.34	2.13E-22	SIG
LA	snp10484	2	49266005	33.67	0.09	2.64	0.36	2.00	2.15E-34	SIG
LA	snp10627	2	50163292	8.22	-0.29	-0.44	0.09	0.50	6.03E-09	SIG
LA	snp15965	4	1022737	36.85	-0.70	0.04	0.22	1.23	1.41E-37	SIG
LA	snp19595	4	39190702	187.68	1.62	3.32	0.24	1.35	2.13E-188	SIG
LA	snp25032	5	38490643	68.34	0.93	0.66	0.81	4.51	4.60E-69	SIG
LA	snp25126	5	39281380	6.77	-0.01	-1.07	0.07	0.41	1.71E-07	SIG
LA	snp25644	6	1490151	17.65	-0.44	0.73	0.21	1.17	2.25E-18	SIG
LA	snp26290	6	5956754	10.60	0.35	-0.31	0.10	0.56	2.50E-11	SIG
LA	snp30327	6	43952755	33.81	0.66	-0.20	0.38	2.09	1.57E-34	SIG
LA	snp34615	7	19847892	6.18	0.00	1.02	0.07	0.36	6.66E-07	SIG
LA	snp35733	7	37646716	13.81	-0.31	-1.79	0.10	0.56	1.56E-14	SIG

LA	snp35787	7	38032815	3.56	-0.17	-0.61	0.03	0.14	2.77E-04	SUG
LA	snp42889	9	1879551	9.29	-0.32	0.51	0.08	0.44	5.18E-10	SIG
LA	snp42918	9	2093064	17.60	0.23	-3.73	0.17	0.96	2.49E-18	SIG
LA	snp47014	9	42559235	12.04	-0.15	1.95	0.15	0.82	9.05E-13	SIG
LA	snp51532	10	44039274	52.67	-0.81	-0.25	0.15	0.84	2.16E-53	SIG
LA	snp52053	10	47257578	10.53	-0.34	-0.44	0.10	0.58	2.98E-11	SIG
LA	snp60500	12	34376573	12.00	-0.38	-0.21	0.08	0.45	1.01E-12	SIG
LA	snp63683	13	21999755	16.18	-0.45	0.09	0.17	0.94	6.57E-17	SIG
LA	snp65615	13	33136412	7.70	0.17	1.49	0.06	0.35	1.99E-08	SIG
LA	snp67026	13	41898777	12.13	-0.38	-0.23	0.09	0.49	7.43E-13	SIG
LA	snp68010	14	4251041	12.77	0.40	-0.17	0.09	0.50	1.69E-13	SIG
LA	snp68383	14	6738549	13.43	0.37	0.70	0.12	0.67	3.72E-14	SIG
LA	snp73359	15	5637661	3.62	0.21	-0.08	0.04	0.23	2.39E-04	SUG
LA	snp73939	15	9420222	10.88	0.31	-0.65	0.12	0.64	1.31E-11	SIG
LA	snp78780	16	735028	15.93	-0.44	-0.29	0.15	0.85	1.19E-16	SIG
LA	snp84224	17	4309255	20.79	0.51	0.03	0.17	0.97	1.63E-21	SIG
LA	snp87462	17	38481517	14.93	-0.42	0.71	0.12	0.69	1.18E-15	SIG
LA	snp88033	18	171641	17.93	0.46	0.32	0.09	0.51	1.17E-18	SIG
LA	snp89429	18	10377738	5.75	-0.28	-0.06	0.06	0.32	1.77E-06	SUG
LA	snp91904	18	39528565	20.48	-0.49	0.70	0.07	0.40	3.34E-21	SIG
LA	snp98695	19	36444979	15.89	0.43	-0.52	0.15	0.81	1.29E-16	SIG
LA	snp99594	19	42267502	5.97	-0.03	-3.05	0.07	0.37	1.08E-06	SUG
LA	snp100593	19	49513282	13.85	0.41	-0.60	0.06	0.36	1.42E-14	SIG
LA	snp104226	20	35265033	16.81	0.06	-2.81	0.19	1.08	1.54E-17	SIG
LNA	snp5827	2	2933071	63.47	0.32	1.39	0.02	0.94	3.42E-64	SIG
LNA	snp21824	5	4230645	37.06	-0.28	-0.22	0.01	0.24	8.82E-38	SIG
LNA	snp22141	5	7106633	112.57	-0.49		0.01	0.33	9.45E-115	SIG
LNA	snp24143	5	31471019	79.78	-0.41		0.01	0.27	7.00E-82	SIG
LNA	snp24551	5	34782642	40.74	-0.29		0.01	0.19	1.05E-42	SIG
LNA	snp26919	6	10299264	88.35	0.42	-2.21	0.03	1.22	4.51E-89	SIG
LNA	snp35581	7	36457795	14.40	0.14	-0.76	0.01	0.45	3.97E-15	SIG
LNA	snp39731	8	19942418	99.25	-0.46	-0.17	0.04	1.56	5.65E-100	SIG
LNA	snp40839	8	35164191	13.93	-0.15	-0.46	0.01	0.33	1.18E-14	SIG
LNA	snp41994	8	43123507	138.05	-0.55	0.92	0.04	1.37	8.95E-139	SIG
LNA	snp42376	8	45289240	124.06	-0.52	-0.04	0.02	0.79	8.79E-125	SIG
LNA	snp43158	9	3532214	31.18	-0.25	-0.15	0.04	1.42	6.66E-32	SIG
LNA	snp53164	11	4257613	20.63	-0.20	-0.09	0.01	0.29	2.32E-21	SIG
LNA	snp53301	11	5071119	44.06	-0.29	-0.75	0.02	0.71	8.71E-45	SIG
LNA	snp54909	11	18115230	40.39	-0.28	0.07	0.01	0.50	4.08E-41	SIG
LNA	snp57755	12	5278680	51.88	0.14	2.45	0.08	2.89	1.34E-52	SIG
LNA	snp62108	13	5557490	35.20	-0.26	-0.15	0.02	0.80	6.26E-36	SIG
LNA	snp64054	13	23929969	112.34	0.50	-0.08	0.01	0.44	4.57E-113	SIG
LNA	snp69771	14	19263306	131.29	-0.53	1.10	0.04	1.40	5.25E-132	SIG
LNA	snp69931	14	22294811	30.61	-0.23	-0.88	0.01	0.40	2.49E-31	SIG

LNA	snp70677	14	31140033	120.34	-0.51	0.52	0.04	1.53	4.59E-121	SIG
LNA	snp78830	16	1001721	78.82	-0.40	-0.03	0.02	0.84	1.51E-79	SIG
LNA	snp83860	17	1360642	29.41	-0.24	-0.40	0.00	0.16	3.87E-30	SIG
LNA	snp84987	17	10652089	29.95	0.19	-1.73	0.03	1.10	1.13E-30	SIG
LNA	snp87820	17	40852330	27.89	-0.24	0.25	0.00	0.18	1.28E-28	SIG
LNA	snp87915	17	41251739	94.00	0.46		0.01	0.32	3.84E-96	SIG
LNA	snp88318	18	2072966	62.56	-0.35	0.66	0.03	1.03	2.77E-63	SIG
LNA	snp94361	18	56993597	47.67	-0.29	-1.06	0.03	1.12	2.13E-48	SIG
LNA	snp95066	18	60190486	17.16	-0.17	-0.43	0.01	0.44	6.97E-18	SIG
OIL	snp7295	2	13653272	7.76	0.09	-1.35	0.05	0.96	1.72E-08	SIG
OIL	snp15689	3	46548091	4.39	-0.17	-0.10	0.02	0.38	4.09E-05	SUG
OIL	snp24247	5	32129897	5.00	0.18	0.21	0.03	0.58	9.89E-06	SUG
OIL	snp25031	5	38490635	18.77	0.35	-0.49	0.13	2.48	1.68E-19	SIG
OIL	snp26441	6	7206352	5.36	0.15	-0.50	0.03	0.65	4.41E-06	SUG
OIL	snp32265	7	5441000	22.74	-0.42	0.11	0.05	0.93	1.80E-23	SIG
OIL	snp35607	7	36747805	8.12	-0.22	0.35	0.05	1.03	7.53E-09	SIG
OIL	snp36613	7	43947021	13.18	0.30	0.28	0.07	1.40	6.63E-14	SIG
OIL	snp39185	8	16500872	17.93	0.35	0.49	0.04	0.70	1.18E-18	SIG
OIL	snp48392	10	6295038	8.83	0.23	0.37	0.06	1.11	1.49E-09	SIG
OIL	snp55833	11	30706647	13.40	0.30	-0.24	0.04	0.69	3.98E-14	SIG
OIL	snp57380	12	3171005	11.26	-0.27	0.38	0.07	1.32	5.53E-12	SIG
OIL	snp64585	13	27855968	4.33	-0.17		0.02	0.41	8.05E-06	SUG
OIL	snp67418	14	372901	8.53	0.01	1.61	0.05	1.04	2.94E-09	SIG
OIL	snp73156	15	4293912	6.47	0.20	0.33	0.02	0.43	3.40E-07	SIG
OIL	snp73806	15	8569014	7.65	0.24	0.07	0.05	0.93	2.25E-08	SIG
OIL	snp82224	16	28711718	5.78	-0.21	0.05	0.03	0.60	1.65E-06	SUG
OIL	snp83568	16	36490513	8.24	0.05	1.28	0.05	0.92	5.70E-09	SIG
OIL	snp87576	17	39156892	22.12	0.40	0.27	0.03	0.48	7.61E-23	SIG
OIL	snp88351	18	2259289	12.94	0.30	-0.22	0.06	1.18	1.14E-13	SIG
OIL	snp88787	18	5359420	11.06	0.28	0.07	0.05	0.91	8.69E-12	SIG
OIL	snp92901	18	50451737	17.58	0.25	2.40	0.06	1.19	2.62E-18	SIG
OIL	snp94601	18	58420889	11.18	0.28	0.18	0.06	1.23	6.67E-12	SIG
OIL	snp95858	19	1780893	7.37	0.23	0.02	0.05	0.86	4.23E-08	SIG
OIL	snp99118	19	38970397	6.63	0.02	-2.58	0.04	0.85	2.36E-07	SIG
OIL	snp103489	20	30581992	46.08	0.58	-0.25	0.10	1.81	8.42E-47	SIG

PA: palmitic acid; SA: stearic acid; OA: oleic acid; LA: linoleic acid; LNA: linolenic acid; OIL: oil content; Chr: chromosome; Pos: position; Add: additive; Dom: dominance; Var: variance; SIG: significant (-log10(P) ≥ 6.04); SUG: suggestion (LOD ≥ 3).

Table S7. QEIs for soybean seed oil-related traits in all the environments using 3VmrMLM

Trait	Marker	Chr	Pos (bp)	LOD (QE)	Var	r^2 (%)	P-value	Significance
PA	snp11768	3	5944719	25.93	0.03	1.90	8.13E-20	SIG
PA	snp11788	3	6164027	21.10	0.02	1.53	1.99E-15	SIG
PA	snp15168	3	42150254	77.65	0.08	6.10	3.64E-74	SIG
PA	snp22240	5	8101709	119.94	0.14	10.05	1.60E-110	SIG
PA	snp23758	5	28271870	18.65	0.02	1.45	3.10E-13	SIG
PA	snp37550	8	6046707	36.66	0.04	2.72	8.33E-30	SIG
PA	snp51483	10	43583670	95.02	0.10	7.58	4.15E-86	SIG
PA	snp89203	18	7840442	36.59	0.04	2.75	9.75E-30	SIG
PA	snp90060	18	15503852	11.82	0.01	0.84	2.28E-07	SIG
PA	snp97471	19	21798341	43.99	0.05	3.42	5.39E-41	SIG
PA	snp98835	19	37280850	74.60	0.08	6.05	3.27E-66	SIG
SA	snp6745	2	9401500	43.34	0.01	3.11	3.99E-36	SIG
SA	snp24808	5	36671535	36.95	0.01	2.71	4.46E-30	SIG
SA	snp25032	5	38490643	10.82	0.00	0.79	1.50E-06	SUG
SA	snp26121	6	4685918	30.48	0.00	2.34	5.08E-24	SIG
SA	snp28582	6	20835485	52.04	0.01	3.88	1.97E-44	SIG
SA	snp32821	7	8047531	18.27	0.00	1.37	6.67E-13	SIG
SA	snp35491	7	35825129	16.83	0.00	1.19	1.24E-11	SIG
SA	snp51538	10	44092582	4.62	0.00	0.33	4.62E-02	SUG
SA	snp51575	10	44410452	11.23	0.00	0.82	6.97E-07	SIG
SA	snp51590	10	44469282	9.47	0.00	0.66	1.77E-05	SUG
SA	snp57125	12	1384742	7.15	0.00	0.50	1.00E-03	SUG
SA	snp60292	12	32790779	38.41	0.01	2.82	1.87E-31	SIG
SA	snp67206	13	43130940	11.27	0.00	0.77	6.43E-07	SIG
SA	snp69105	14	11765489	19.48	0.00	1.40	5.68E-14	SIG
SA	snp94922	18	59528591	11.97	0.00	0.84	1.71E-07	SIG
SA	snp97034	19	12199744	37.68	0.01	2.82	9.17E-31	SIG
SA	snp103976	20	33904632	9.00	0.00	0.67	4.13E-05	SUG
OA	snp11943	3	8113468	21.07	0.38	1.32	2.11E-15	SIG
OA	snp25014	5	38379318	10.52	0.18	0.64	2.62E-06	SUG
OA	snp25633	6	1436603	11.46	0.21	0.72	4.56E-07	SIG
OA	snp25644	6	1490151	13.45	0.24	0.84	1.00E-08	SIG
OA	snp33258	7	10264105	8.17	0.15	0.54	1.76E-04	SUG
OA	snp36510	7	43205262	13.41	0.24	0.85	1.09E-08	SIG
OA	snp47064	9	42919313	10.66	0.19	0.65	2.01E-06	SUG
OA	snp47463	9	46005575	13.88	0.24	0.83	4.29E-09	SIG
OA	snp51556	10	44206757	15.15	0.27	0.95	3.58E-10	SIG
OA	snp51573	10	44405881	17.16	0.29	1.02	6.37E-12	SIG
OA	snp54194	11	11277577	17.16	0.30	1.04	6.33E-12	SIG
OA	snp63708	13	22157524	35.50	0.61	2.14	1.02E-28	SIG
OA	snp63721	13	22237579	27.65	0.47	1.64	2.10E-21	SIG
OA	snp97160	19	15500421	24.40	0.44	1.54	2.04E-18	SIG

LA	snp11943	3	8113468	22.47	0.27	1.52	1.15E-16	SIG
LA	snp24284	5	32409023	6.57	0.08	0.43	2.55E-03	SUG
LA	snp25032	5	38490643	13.30	0.16	0.87	1.33E-08	SIG
LA	snp25633	6	1436603	8.09	0.10	0.58	2.03E-04	SUG
LA	snp26465	6	7393451	29.81	0.35	1.95	3.79E-27	SIG
LA	snp33280	7	10409939	11.19	0.13	0.73	7.50E-07	SIG
LA	snp39463	8	18183299	8.69	0.10	0.55	7.15E-05	SUG
LA	snp51590	10	44469282	7.40	0.09	0.49	6.54E-04	SUG
LA	snp54194	11	11277577	15.96	0.19	1.04	7.05E-11	SIG
LA	snp63721	13	22237579	9.36	0.11	0.60	2.17E-05	SUG
LA	snp66754	13	40247049	26.61	0.32	1.77	1.93E-20	SIG
LA	snp85477	17	13741950	29.31	0.38	2.08	6.22E-23	SIG
LA	snp97160	19	15500421	17.32	0.21	1.18	4.58E-12	SIG
OIL	snp28789	6	22492749	37.05	0.25	4.77	9.64E-33	SIG
OIL	snp52940	11	2571951	6.47	0.04	0.79	2.28E-04	SUG
OIL	snp63233	13	14402337	6.43	0.04	0.81	2.47E-04	SUG
OIL	snp83910	17	1802351	9.11	0.06	1.17	1.37E-06	SUG
OIL	snp98241	19	32712483	25.87	0.17	3.28	4.99E-22	SIG

PA: palmitic acid; SA: stearic acid; OA: oleic acid; LA: linoleic acid; LNA: linolenic acid; OIL: oil content; Chr: chromosome; Pos: position; Add: additive; Dom: dominance; Var: variance; SIG: significant (-log10(P) ≥ 6.04); SUG: suggestion (LOD ≥ 3).

Table S8. Haplotype analyses for 83 candidate genes of soybean seed oil-related traits

Gene	SNP				Wild soybeans		Landrace soybeans		Bred soybeans	
	No.	Genome region	No. haplotype	Elite haplotype	No. haplotype	% Elite haplotype	No. haplotype	% Elite haplotype	No. haplotype	% Elite haplotype
Glyma01g36011	18	UP, 5'UTR, 3'UTR	7	Hap1, Hap2	6	8.00	3	95.77	3	85.71
Glyma01g36680	2	3'UTR, CDS	4	Hap1	4	55.00	2	89.02	2	97.56
Glyma01g36680	4	UP	4	Hap1, Hap2	4	82.05	2	100.00	2	100.00
Glyma01g43780	4	UP, 5'UTR	4	Hap2	3	0.00	4	20.78	3	58.33
Glyma02g15600	11	UP	6	Hap1, Hap3	4	6.45	6	70.83	3	91.18
Glyma02g15650	2	UP	2	Hap2	2	2.63	2	34.18	2	20.00
Glyma02g42390	28	UP	9	Hap1, Hap2	7	0.00	4	97.37	2	95.24
Glyma03g34740	5	3'UTR, UP	6	Hap1, Hap4	6	38.89	4	70.67	3	97.30
Glyma03g41770	4	UP, 5'UTR	4	Hap1, Hap4	4	8.57	4	81.58	4	81.08
Glyma04g01540	3	UP, 5'UTR, CDS	4	Hap2	4	3.03	4	25.32	2	63.89
Glyma04g11550	8	UP, 5'UTR, CDS, 3'UTR	5	Hap2	4	0.00	4	16.90	4	56.76
Glyma04g41220	4	UP	4	Hap1	4	20.00	4	53.95	2	87.80
Glyma04g41450	8	UP, 5'UTR, CDS	5	Hap1, Hap2	4	4.00	5	73.33	2	100.00
Glyma05g04180	7	3'UTR	5	Hap1, Hap2	3	31.03	4	78.87	3	96.97
Glyma05g07880	7	UP, 5'UTR	4	Hap2	3	7.50	4	14.47	2	62.16
Glyma05g07880	3	5'UTR, 3'UTR	4	Hap1, Hap2	3	15.79	3	97.40	2	100.00
Glyma05g08060	3	5'UTR, 3'UTR	4	Hap2	3	0.00	4	12.50	2	56.76
Glyma06g01240	7	3'UTR, CDS, 5'UTR, UP	6	Hap1, Hap2, Hap3	5	64.29	3	100.00	3	100.00
Glyma06g01460	12	CDS, UP	4	Hap2	4	11.76	4	31.43	3	92.50
Glyma06g08290	4	3'UTR, UP	5	Hap1	5	30.56	3	81.25	1	100.00
Glyma06g19560	2	3'UTR	2	Hap1	2	74.29	2	93.67	2	97.56
Glyma07g07560	8	UP, 5'UTR	7	Hap2, Hap3	5	0.00	6	43.06	4	88.57
Glyma07g07560	2	3'UTR	3	Hap2	3	15.79	3	24.32	2	64.10
Glyma07g07580	6	5'UTR, UP	6	Hap2	6	2.94	5	18.31	3	57.89
Glyma07g09370	16	UP, CDS	8	Hap1	6	4.76	6	51.52	7	45.45
Glyma07g11890	20	3'UTR, 5'UTR, UP	6	Hap1, Hap3	4	0.00	3	98.68	2	100.00
Glyma07g30500	7	3'UTR, 5'UTR, UP	4	Hap1	4	3.45	3	50.70	3	82.86
Glyma07g32750	6	CDS, 5'UTR, UP	5	Hap1, Hap2	4	5.26	2	100.00	2	100.00
Glyma07g32780	7	3'UTR, CDS, UP	6	Hap1, Hap3	5	6.45	3	82.19	3	91.67
Glyma07g32850	15	UP	4	Hap1	4	25.93	2	90.41	2	92.11

Glyma07g33840	2	UP	2	Hap1	2	2.38	2	65.38	1	100.00
Glyma07g34030	5	CDS, 5'UTR, UP	5	Hap1	4	0.00	4	82.89	2	92.50
Glyma07g38910	10	UP, 5'UTR, CDS, 3'UTR	7	Hap1	4	0.00	4	68.42	3	92.31
Glyma07g39120	7	5'UTR, UP, CDS	4	Hap1	3	0.00	3	87.34	1	100.00
Glyma08g06820	9	UP, 5'UTR	6	Hap1, Hap2	5	6.67	3	81.43	3	87.88
Glyma08g13290	5	UP, CDS, 3'UTR	7	Hap3	7	6.06	4	8.11	4	35.90
Glyma08g19720	9	UP	4	Hap1, Hap4	4	41.18	3	98.77	1	100.00
Glyma08g26040	5	UP	2	Hap1	2	60.00	2	95.00	1	100.00
Glyma08g45990	3	UP	4	Hap4	2	0.00	4	7.79	3	35.14
Glyma09g04000	3	UP	4	Hap1, Hap2	4	5.00	4	74.03	3	94.74
Glyma09g34110	6	UP, CDS, 3'UTR	5	Hap2	4	0.00	5	15.07	3	52.63
Glyma09g34770	25	UP, 5'UTR	8	Hap2	7	4.00	7	23.19	3	51.61
Glyma09g36920	16	UP	7	Hap1, Hap2	6	16.67	3	98.51	2	100.00
Glyma09g38260	31	3'UTR, 5'UTR, UP	8	Hap2	7	0.00	3	23.81	3	64.71
Glyma09g38400	10	UP	8	Hap1, Hap2	5	17.86	4	65.22	3	87.88
Glyma09g41730	17	3'UTR, CDS, UP	6	Hap1	6	3.23	5	49.28	4	84.85
Glyma10g05750	16	UP, 5'UTR	6	Hap1, Hap2	5	24.14	3	80.82	2	100.00
Glyma10g05750	2	5'UTR	2	Hap2	2	4.44	2	27.27	2	7.89
Glyma10g07520	2	3'UTR, CDS	3	Hap1, Hap2	3	71.43	3	77.50	2	100.00
Glyma10g07520	2	5'UTR, UP	4	Hap1	4	56.41	3	74.39	1	100.00
Glyma10g34490	18	3'UTR, CDS, UP	4	Hap1	4	2.94	3	75.32	1	100.00
Glyma10g36370	15	UP, 5'UTR, 3'UTR	5	Hap1, Hap2	5	6.06	3	95.59	3	93.75
Glyma12g03060	6	UP	4	Hap1, Hap2	4	64.71	3	88.31	2	100.00
Glyma13g11700	4	UP	6	Hap2, Hap3	4	0.00	5	54.29	3	91.89
Glyma13g18220	3	CDS, UP	5	Hap1	5	25.64	4	69.51	1	100.00
Glyma13g36730	11	3'UTR, CDS, 5'UTR, UP	7	Hap1, Hap2	7	7.69	3	98.57	2	100.00
Glyma14g00760	5	UP	5	Hap1	5	9.09	3	30.00	3	63.89
Glyma14g37130	5	UP, 5'UTR, 3'UTR	5	Hap1	5	6.06	4	88.75	2	78.95
Glyma15g05470	7	3'UTR, CDS	3	Hap1	3	6.90	3	82.50	1	100.00
Glyma15g07860	17	3'UTR, CDS, UP	6	Hap1, Hap2	4	0.00	3	97.14	3	93.33
Glyma15g07900	4	5'UTR, 3'UTR	5	Hap1, Hap2	4	13.89	2	100.00	2	100.00
Glyma15g11530	10	3'UTR, CDS, 5'UTR, UP	6	Hap1, Hap2	6	16.67	3	98.65	2	100.00
Glyma15g15310	9	UP, CDS, 3'UTR	8	Hap1	6	0.00	4	72.60	3	95.00
Glyma16g01070	3	CDS, 5'UTR, UP	4	Hap1, Hap3	4	5.13	3	72.37	3	72.22
Glyma16g01470	5	CDS, 5'UTR	3	Hap1	3	30.00	2	96.30	1	100.00
Glyma16g02090	3	5'UTR	4	Hap1	3	0.00	3	90.24	3	73.17

Glyma16g02090	6	UP	5	Hap2	5	3.33	2	7.50	2	28.95
Glyma16g21960	2	stop	3	Hap1	3	2.78	3	55.26	3	72.97
Glyma16g32130	3	3'UTR, CDS, 5'UTR	4	Hap1	4	23.53	3	69.62	2	92.31
Glyma16g33510	5	CDS, UP	5	Hap2, Hap3, Hap4	4	51.43	4	60.53	3	87.50
Glyma17g06120	8	UP, CDS	8	Hap1	7	22.22	4	59.15	3	77.78
Glyma17g14620	10	3'UTR, CDS, UP	10	Hap3	8	0.00	5	16.44	4	18.42
Glyma17g14810	6	UP	5	Hap2	5	3.12	3	8.22	3	38.24
Glyma17g14950	37	3'UTR, 5'UTR, UP	5	Hap2	4	25.00	4	31.58	3	59.46
Glyma17g34960	3	UP	4	Hap1, Hap2	4	35.14	3	83.10	3	97.50
Glyma17g36940	12	UP, 5'UTR, CDS, 3'UTR	9	Hap1, Hap2, Hap3	9	22.73	4	98.65	3	100.00
Glyma18g03090	10	3'UTR, CDS, UP	6	Hap1, Hap2	6	34.48	5	79.45	3	97.44
Glyma18g03100	7	CDS, 5'UTR, UP	5	Hap2	5	5.26	4	10.00	4	69.70
Glyma18g06500	7	3'UTR, 5'UTR, UP	6	Hap1, Hap2	5	30.77	4	69.33	3	89.19
Glyma18g41320	3	3'UTR	4	Hap1	4	39.39	4	82.28	1	100.00
Glyma18g41320	8	UP	5	Hap1, Hap4, Hap5	4	26.67	4	93.51	1	100.00
Glyma18g41590	6	UP	2	Hap1	2	6.06	2	89.61	1	100.00
Glyma18g50020	27	UP, 3'UTR	6	Hap2, Hap3, Hap4	4	19.23	5	63.49	5	63.64
Glyma18g51600	4	5'UTR, CDS, 3'UTR	4	Hap1, Hap2, Hap4	3	66.67	4	97.40	3	97.37
Glyma18g51600	4	3'UTR	3	Hap1	3	22.22	3	64.10	3	90.00
Glyma19g07410	7	3'UTR, 5'UTR, UP	5	Hap1, Hap3	5	34.48	3	62.50	3	97.30
Glyma19g27060	2	5'UTR, UP	3	Hap1, Hap2	3	66.67	3	92.59	2	100.00
Glyma19g44410	17	UP, 5'UTR	10	Hap1, Hap3	9	22.22	6	83.12	2	91.89
Glyma19g44410	3	CDS, 3'UTR	4	Hap1	4	35.29	3	90.36	1	100.00
Glyma20g01180	11	UP, 5'UTR, CDS, 3'UTR	8	Hap1, Hap2	8	12.50	3	97.33	3	97.44
Glyma20g26280	12	UP	7	Hap1	6	0.00	5	64.79	3	94.74
Glyma20g26280	5	5'UTR, 3'UTR	3	Hap1	3	25.00	3	62.34	3	92.68
Glyma20g31200	5	CDS, UP	6	Hap1	6	6.67	3	72.73	1	100.00

CDS: coding sequence; UTR: untranslated region; UP: upstream.

Table S9. 76 candidate genes around QTNs for soybean seed oi-related traits

Traits	Dataset	Marker	Chr	Pos (bp)	LOD	Add	Dom	r^2(%)	P-value	Signifi	Gene	Homologous gene in Arabidopsis		
												Gene	Symbol	Annotation
PA	II	snp20863	4	47384506	12.56	-0.28	-0.17	3.28	2.74E-13	SIG	*Glyma04g41450*	*AT5G24240.1*		Phosphatidylinositol 3- and 4-kinase; Ubiquitin family protein
PA	IV	snp15168	3	42150254	38.67	-0.80	NA	3.14	1.28E-40	SIG	*Glyma03g34740*	*AT3G52990.1*	PK	Pyruvate kinase family protein
PA	IV	snp35454	7	35530610	18.06	-0.43	1.82	7.77	8.80E-19	SIG	*Glyma07g30500*	*AT1G50090.1*		D-aminoacid aminotransferase-like PLP-dependent enzymes superfamily protein
PA	IX	snp26308	6	6123748	6.78	0.08	0.05	0.43	1.67E-07	SIG	*Glyma06g08290*	*AT2G25890.1*		Oleosin family protein
PA	IX	snp73350	15	5592008	79.50	-0.31	0.05	1.18	3.19E-80	SIG	*Glyma15g07860*	*AT3G48560.1*	CSR1	chlorsulfuron/imidazolinone resistant 1
PA	IX	snp73350	15	5592008	79.50	-0.31	0.05	1.18	3.19E-80	SIG	*Glyma15g07900*	*AT4G04930.1*	DES-1-LIKE	fatty acid desaturase family protein
PA	IX	snp104335	20	35860893	8.90	0.00	0.35	0.60	1.26E-09	SIG	*Glyma20g26280*	*AT3G19420.1*	PEN2	PTEN 2
PA	IX	snp104975	20	39923283	10.36	-0.01	-0.34	0.70	4.38E-11	SIG	*Glyma20g31200*	*AT1G14290.1*	SBH2	sphingoid base hydroxylase 2
PA	VI	snp73350	15	5592008	43.32	-0.46	0.11	6.24	4.77E-44	SIG	*Glyma15g07860*	*AT3G48560.1*	CSR1	chlorsulfuron/imidazolinone resistant 1
PA	VI	snp73350	15	5592008	43.32	-0.46	0.11	6.24	4.77E-44	SIG	*Glyma15g07900*	*AT4G04930.1*	DES-1-LIKE	fatty acid desaturase family protein
PA	VI	snp100677	19	49890890	20.58	-0.27	NA	2.06	2.13E-22	SIG	*Glyma19g44410*	*AT3G61680.1*		alpha/beta-Hydrolases superfamily protein
PA	VII	snp22184	5	7711523	10.58	0.22	-0.18	2.58	2.62E-11	SIG	*Glyma05g07880* (*GmOLE2*)	*AT3G18570.1*	OLE	Oleosin family protein
PA	VIII	snp20788	4	47019480	30.68	-0.28	0.10	4.13	2.09E-31	SIG	*Glyma04g41220*	*AT4G27780.1*	ACBP2	acyl-CoA binding protein 2
PA	VIII	snp26308	6	6123748	8.96	0.14	0.05	3.97	1.11E-09	SIG	*Glyma06g08290*	*AT2G25890.1*		Oleosin family protein
PA	VIII	snp46685	9	40624897	6.75	-0.13	-0.05	3.16	1.80E-07	SIG	*Glyma09g34110*	*AT1G32200.2*	ACT1	phospholipid/glycerol acyltransferase family protein
PA	VIII	snp47176	9	43672155	9.48	-0.14	0.13	4.83	3.31E-10	SIG	*Glyma09g38260*	*AT2G44620.1*	mtACP1	mitochondrial acyl carrier protein 1
PA	VIII	snp47176	9	43672155	9.48	-0.14	0.13	4.83	3.31E-10	SIG	*Glyma09g38400*	*AT1G65290.1*	mtACP2	mitochondrial acyl carrier protein 2
PA	VIII	snp73350	15	5592008	23.50	-0.25	0.43	3.40	3.19E-24	SIG	*Glyma15g07860*	*AT3G48560.1*	CSR1	chlorsulfuron/imidazolinone resistant 1
PA	VIII	snp73350	15	5592008	23.50	-0.25	0.43	3.40	3.19E-24	SIG	*Glyma15g07900*	*AT4G04930.1*	DES-1-LIKE	fatty acid desaturase family protein
PA	VIII	snp104335	20	35860893	7.24	0.04	0.44	2.97	5.81E-08	SIG	*Glyma20g26280*	*AT3G19420.1*	PEN2	PTEN 2
PA	VIII	snp104975	20	39923283	11.78	-0.10	-0.44	5.53	1.66E-12	SIG	*Glyma20g31200*	*AT1G14290.1*	SBH2	sphingoid base hydroxylase 2

SA	I	snp51590	10	44469282	11.49	-0.12	-0.05	5.50	3.21E-12	SIG	*Glyma10g36370*	*AT1G14290.1*	SBH2	sphingoid base hydroxylase 2
SA	I	snp85152	17	11602669	20.31	-0.16	0.09	2.49	4.90E-21	SIG	*Glyma17g14810*	*AT4G22550.1*	LPPβ	Phosphatidic acid phosphatase (PAP2) family protein
SA	I	snp85152	17	11602669	20.31	-0.16	0.09	2.49	4.90E-21	SIG	*Glyma17g14950*	*AT4G17260.1*	LDG1	Lactate/malate dehydrogenase family protein
SA	II	snp15761	3	47212539	6.66	0.07	0.09	2.38	2.18E-07	SIG	*Glyma03g41770*	*AT3G61680.1*		alpha/beta-Hydrolases superfamily protein
SA	II	snp32787	7	7895362	4.24	0.06	-0.11	2.67	5.78E-05	SUG	*Glyma07g09370*	*AT4G29010.1*	AIM1	Enoyl-CoA hydratase/isomerase family
SA	II	snp51590	10	44469282	9.49	-0.09	-0.06	4.95	3.21E-10	SIG	*Glyma10g36370*	*AT1G14290.1*	SBH2	sphingoid base hydroxylase 2
SA	II	snp71936	14	46333223	11.35	-0.09	-0.22	2.87	4.48E-12	SIG	*Glyma14g37130*	*AT5G58860.1*	CYP86A1	cytochrome P450, family 86, subfamily A, polypeptide 1
SA	II	snp83303	16	35331833	5.76	0.07	0.09	2.65	1.72E-06	SUG	*Glyma16g32130*	*AT4G00730.1*	ANL2	Homeobox-leucine zipper family protein / lipid-binding START domain-containing protein
SA	II	snp94841	18	59249935	10.56	-0.06	0.49	5.47	2.74E-11	SIG	*Glyma18g50020*	*AT5G15530.1*	BCCP2	biotin carboxyl carrier protein 2
SA	III	snp37412	8	4895288	11.57	0.11	0.00	1.57	2.72E-12	SIG	*Glyma08g06820*	*AT5G09660.1*	PMDH2	peroxisomal NAD-malate dehydrogenase 2
SA	III	snp43067	9	2918055	12.98	-0.12	-0.12	5.29	1.04E-13	SIG	*Glyma09g04000*	*AT3G06650.1*	ACLB1	ATP-citrate lyase B-1
SA	III	snp87552	17	38989203	5.76	-0.08	-0.08	1.68	1.73E-06	SUG	*Glyma17g34960*	*AT2G34590.1*	PDHE1β	Transketolase family protein
SA	IX	snp47001	9	42417831	5.61	-0.03	-0.06	0.18	2.44E-06	SUG	*Glyma09g36920*	*AT1G31480.1*	SGR2	shoot gravitropism 2 (SGR2)
SA	IX	snp51590	10	44469282	12.29	-0.04	0.00	0.77	5.13E-13	SIG	*Glyma10g36370*	*AT1G14290.1*	SBH2	sphingoid base hydroxylase 2
SA	IX	snp83279	16	35261831	12.03	-0.04	0.07	0.92	9.42E-13	SIG	*Glyma16g32130*	*AT4G00730.1*	ANL2	Homeobox-leucine zipper family protein / lipid-binding START domain-containing protein
SA	IX	snp98399	19	34215110	21.31	-0.06	0.10	0.64	4.91E-22	SIG	*Glyma19g27060*	*AT3G02040.1*	SRG3	senescence-related gene 3
SA	IX	snp100911	20	852754	9.83	-0.02	-0.17	0.53	1.49E-10	SIG	*Glyma20g01180*	*AT2G33150.1*		peroxisomal 3-ketoacyl-CoA thiolase 3
SA	V	snp51595	10	44535628	4.19	-0.10	0.10	4.45	6.49E-05	SUG	*Glyma10g36370*	*AT1G14290.1*	SBH2	sphingoid base hydroxylase 2
SA	VI	snp15762	3	47243699	11.91	0.12	-0.03	2.91	1.23E-12	SIG	*Glyma03g41770*	*AT3G61680.1*		alpha/beta-Hydrolases superfamily protein
SA	VI	snp78912	16	1594037	7.73	0.00	0.46	3.17	1.87E-08	SIG	*Glyma16g02090*	*AT1G01090.1*	PDHE1α	pyruvate dehydrogenase E1 alpha
SA	VI	snp92846	18	50082146	21.11	-0.17	-0.07	4.23	7.80E-22	SIG	*Glyma18g41320*	*AT3G53980.2*	LTP12	Bifunctional inhibitor/lipid-transfer protein/seed storage 2S albumin superfamily protein
SA	VII	snp5250	1	54716009	6.30	0.09	0.08	3.62	5.07E-07	SIG	*Glyma01g43780*	*AT4G10020.1*	HSD5	hydroxysteroid dehydrogenase 5
SA	VII	snp83298	16	35319982	8.45	0.09	-0.30	5.15	3.58E-09	SIG	*Glyma16g32130*	*AT4G00730.1*	ANL2	Homeobox-leucine zipper family protein / lipid-binding START

														domain-containing protein
OA	II	snp21648	5	3363617	29.47	-1.69	-3.05	3.75	3.37E-30	SIG	*Glyma05g04180*	*AT1G62790.2*		Bifunctional inhibitor/lipid-transfer protein/seed storage 2S albumin superfamily protein
OA	III	snp10218	2	47425835	9.32	1.13	1.00	2.62	4.78E-10	SIG	*Glyma02g42390*	*AT5G05690.1*	CYP90A1	Cytochrome P450 superfamily protein
OA	III	snp22209	5	7887398	27.35	2.09	1.08	3.26	4.48E-28	SIG	*Glyma05g07880* (*GmOLE2*)	*AT3G18570.1*	OLE	Oleosin family protein
OA	III	snp37412	8	4895288	18.58	-1.68	0.12	3.60	2.64E-19	SIG	*Glyma08g06820*	*AT5G09660.1*	PMDH2	peroxisomal NAD-malate dehydrogenase 2
OA	IV	snp66380	13	38054635	12.32	1.12	-1.73	8.52	4.80E-13	SIG	*Glyma13g36730*	*AT3G58610.3*		ketol-acid reductoisomerase
OA	IX	snp32422	7	6246072	22.66	-0.58	-1.02	1.33	2.17E-23	SIG	*Glyma07g07560*	*AT2G45970.1*	CYP86A8	cytochrome P450, family 86, subfamily A, polypeptide 8
OA	IX	snp32422	7	6246072	22.66	-0.58	-1.02	1.33	2.17E-23	SIG	*Glyma07g07580*	*AT4G00400.1*	GPAT8	glycerol-3-phosphate acyltransferase 8
OA	IX	snp36568	7	43611859	13.29	0.59	-0.15	0.59	5.14E-14	SIG	*Glyma07g38910*	*AT4G04870.1*	CLS	cardiolipin synthase
OA	IX	snp36568	7	43611859	13.29	0.59	-0.15	0.59	5.14E-14	SIG	*Glyma07g39120*	*AT1G11840.6*	GLX1	glyoxalase I homolog
OA	IX	snp38127	8	9802623	10.21	0.42	0.63	0.63	6.12E-11	SIG	*Glyma08g13290*	*AT2G31810.1*		ACT domain-containing small subunit of acetolactate synthase protein
OA	IX	snp63683	13	21999755	23.99	0.65	0.56	1.22	1.03E-24	SIG	*Glyma13g18220*	*AT2G42450.1*		alpha/beta-Hydrolases superfamily protein
OA	VI	snp63683	13	21999755	7.87	1.35	-1.59	3.50	1.36E-08	SIG	*Glyma13g18220*	*AT2G42450.1*		alpha/beta-Hydrolases superfamily protein
OA	VII	snp27810	6	15772859	7.01	0.07	4.36	3.65	9.78E-08	SIG	*Glyma06g19560*	*AT3G48000.1*	ALDH2	aldehyde dehydrogenase 2B4
OA	VII	snp96763	19	8799955	7.03	-0.53	6.81	3.28	9.43E-08	SIG	*Glyma19g07410*	*AT5G40610.1*	G3PD	NAD-dependent glycerol-3-phosphate dehydrogenase family protein
OA	VIII	snp63683	13	21999755	9.14	0.62	-0.39	2.25	7.18E-10	SIG	*Glyma13g18220*	*AT2G42450.1*		alpha/beta-Hydrolases superfamily protein
OA	VIII	snp78780	16	735028	10.09	0.61	-1.01	2.57	8.05E-11	SIG	*Glyma16g01070*	*AT1G17420.1*	LOX3	lipoxygenase 3
LA	I	snp27810	6	15772859	10.02	0.09	-3.46	4.69	9.51E-11	SIG	*Glyma06g19560*	*AT3G48000.1*	ALDH2	aldehyde dehydrogenase 2B4
LA	I	snp88748	18	5130475	7.51	0.27	-4.47	3.52	3.12E-08	SIG	*Glyma18g06500*	*AT2G30200.1*	FabD	catalytics; transferases; [acyl-carrier-protein] S-malonyltransferases; binding
LA	III	snp51348	10	42589120	8.07	0.07	3.33	5.66	8.44E-09	SIG	*Glyma10g34490*	*AT3G52990.1*	PK90	Pyruvate kinase family protein
LA	III	snp63683	13	21999755	4.80	-0.70	-0.07	3.11	1.57E-05	SUG	*Glyma13g18220*	*AT2G42450.1*		alpha/beta-Hydrolases superfamily protein
LA	IV	snp15965	4	1022737	5.31	-0.59	0.44	1.89	4.94E-06	SUG	*Glyma04g01540*	*AT1G49340.1*	PI4Kα	Phosphatidylinositol 3- and 4-kinase family protein
LA	IV	snp46758	9	41114554	7.64	-0.65	1.22	4.45	2.29E-08	SIG	*Glyma09g34770*	*AT1G31812.1*	ACBP6	acyl-CoA-binding protein 6

LA	IV	snp57221	12	2065602	6.49	0.14	3.74	3.44	3.21E-07	SIG	*Glyma12g03060*	*AT2G05990.2*	ENR1	NAD(P)-binding Rossmann-fold superfamily protein
LA	IX	snp15965	4	1022737	36.85	-0.70	0.04	1.23	1.41E-37	SIG	*Glyma04g01540*	*AT1G49340.1*	PI4Kα	Phosphatidylinositol 3- and 4-kinase family protein
LA	IX	snp35733	7	37646716	13.81	-0.31	-1.79	0.56	1.56E-14	SIG	*Glyma07g32750*	*AT2G43790.1*	MPK6	MAP kinase 6
LA	IX	snp35733	7	37646716	13.81	-0.31	-1.79	0.56	1.56E-14	SIG	*Glyma07g32780*	*AT3G59770.1*	SAC9	sacI homology domain-containing protein / WW domain-containing protein
LA	IX	snp47014	9	42559235	12.04	-0.15	1.95	0.82	9.05E-13	SIG	*Glyma09g36920*	*AT1G31480.1*	SGR2	shoot gravitropism 2 (SGR2)
LA	IX	snp63683	13	21999755	16.18	-0.45	0.09	0.94	6.57E-17	SIG	*Glyma13g18220*	*AT2G42450.1*		alpha/beta-Hydrolases superfamily protein
LA	IX	snp73359	15	5637661	3.62	0.21	-0.08	0.23	2.39E-04	SUG	*Glyma15g07900*	*AT4G04930.1*	DES-1-LIKE	fatty acid desaturase family protein
LA	IX	snp78780	16	735028	15.93	-0.44	-0.29	0.85	1.19E-16	SIG	*Glyma16g01070*	*AT1G17420.1*	LOX3	lipoxygenase 3
LA	V	snp35897	7	38879620	15.08	-1.25	0.15	2.44	8.35E-16	SIG	*Glyma07g34030*	*AT1G71010.1*	FAB1C	FORMS APLOID AND BINUCLEATE CELLS 1C
LA	V	snp35897	7	38879620	15.08	-1.25	0.15	2.44	8.35E-16	SIG	*Glyma07g33840*	*AT4G34100.2*	SUD1	RING/U-box superfamily protein
LA	V	snp48194	10	4490676	18.20	1.39	1.47	3.95	6.30E-19	SIG	*Glyma10g05750*	*AT5G03080.1*	LPPγ	Phosphatidic acid phosphatase (PAP2) family protein
LA	VIII	snp63683	13	21999755	9.95	-0.52	0.93	3.20	1.13E-10	SIG	*Glyma13g18220*	*AT2G42450.1*		alpha/beta-Hydrolases superfamily protein
LA	VIII	snp78780	16	735028	10.79	-0.58	-0.62	2.96	1.61E-11	SIG	*Glyma16g01070*	*AT1G17420.1*	LOX3	lipoxygenase 3
LNA	I	snp87557	17	39024091	10.97	-0.39	-0.42	2.20	1.08E-11	SIG	*Glyma17g34960*	*AT2G34590.1*	PDHE1β	Transketolase family protein
LNA	I	snp95117	18	60388330	30.37	-0.69	NA	2.15	2.84E-32	SIG	*Glyma18g51600*	*AT1G13280.1*	AOC4	allene oxide cyclase 4
LNA	II	snp74273	15	11765119	19.89	-0.53	0.43	4.85	1.30E-20	SIG	*Glyma15g15310*	*AT4G16155.1*	DLD	dihydrolipoyl dehydrogenases
LNA	II	snp81558	16	25313553	9.37	-0.35	0.10	2.81	4.27E-10	SIG	*Glyma16g21960* (*GmDGAT2B*)	*AT3G51520.1*	DGAT	diacylglycerol acyltransferase family
LNA	IV	snp4487	1	49157127	14.54	-0.45	0.49	2.06	2.87E-15	SIG	*Glyma01g36680* (*GmPLDδ2*)	*AT4G35790.2*	PLDδ	phospholipase D delta
LNA	IX	snp42376	8	45289240	124.06	-0.52	-0.04	0.79	8.79E-125	SIG	*Glyma08g45990*	*AT2G05990.2*	MOD1	NAD(P)-binding Rossmann-fold superfamily protein
LNA	IX	snp78830	16	1001721	78.82	-0.40	-0.03	0.84	1.51E-79	SIG	*Glyma16g01470*	*AT3G14205.1*		Phosphoinositide phosphatase family protein
LNA	IX	snp87820	17	40852330	27.89	-0.24	0.25	0.18	1.28E-28	SIG	*Glyma17g36940*	*AT1G19440.1*	KCS4	3-ketoacyl-CoA synthase 4
LNA	IX	snp88318	18	2072966	62.56	-0.35	0.66	1.03	2.77E-63	SIG	*Glyma18g03090*	*AT3G08510.2*	PLC2	phospholipase C 2
LNA	IX	snp88318	18	2072966	62.56	-0.35	0.66	1.03	2.77E-63	SIG	*Glyma18g03100*	*AT2G40116.1*		Phosphoinositide-specific phospholipase C family protein

LNA	V	snp38903	8	14811585	4.36	-0.23	0.12	1.45	4.37E-05	SUG	*Glyma08g19720*	*AT1G33270.1*		Acyl transferase/acyl hydrolase / lysophospholipase superfamily protein
LNA	V	snp39805	8	20452423	15.71	-0.46	-0.53	2.22	1.97E-16	SIG	*Glyma08g26040*	*AT1G22620.1*	SAC1	Phosphoinositide phosphatase family protein
LNA	V	snp47504	9	46335989	32.15	-0.69	-0.69	1.86	7.06E-33	SIG	*Glyma09g41730*	*AT5G01670.1*		NAD(P)-linked oxidoreductase superfamily protein
LNA	VI	snp25587	6	961556	25.72	-0.54	-0.81	3.47	1.91E-26	SIG	*Glyma06g01460*	*AT1G19440.1*	KCS4	3-ketoacyl-CoA synthase 4
LNA	VII	snp88723	18	5041385	7.02	-0.26	-0.01	1.33	9.57E-08	SIG	*Glyma18g06500*	*AT2G30200.1*	FabD	catalytics; transferases; [acyl-carrier-protein] S-malonyltransferases; binding
LNA	VIII	snp33232	7	10138673	7.18	-0.23	0.34	1.57	6.62E-08	SIG	*Glyma07g11890*	*AT3G17810.1*	PYD1	pyrimidine 1
OIL	IV	snp17323	4	9983937	12.23	0.69	1.12	5.87	5.96E-13	SIG	*Glyma04g11550*	*AT1G36160.2*	ACC1	acetyl-CoA carboxylase 1
OIL	IX	snp48392	10	6295038	8.83	0.23	0.37	1.11	1.49E-09	SIG	*Glyma10g07520*	*AT5G03770.1*	KDTA	KDO transferase A
OIL	IX	snp67418	14	372901	8.53	0.01	1.61	1.04	2.94E-09	SIG	*Glyma14g00760*	*AT5G48230.2*	ACAT2	acetoacetyl-CoA thiolase 2
OIL	IX	snp73806	15	8569014	7.65	0.24	0.07	0.93	2.25E-08	SIG	*Glyma15g11530*	*AT1G11840.6*	GLX1	glyoxalase I homolog
OIL	IX	snp83568	16	36490513	8.24	0.05	1.28	0.92	5.70E-09	SIG	*Glyma16g33510*	*AT5G08370.1*	GAL2	alpha-galactosidase 2
OIL	IX	snp92901	18	50451737	17.58	0.25	2.40	1.19	2.62E-18	SIG	*Glyma18g41590*	*AT5G55410.1*		Bifunctional inhibitor/lipid-transfer protein/seed storage 2S albumin superfamily protein
OIL	V	snp63233	13	14402337	7.95	-0.50	-0.21	4.05	1.13E-08	SIG	*Glyma13g11700*	*AT2G04350.2*	LACS8	AMP-dependent synthetase and ligase family protein
OIL	VI	snp7349	2	14128122	6.29	0.45	-0.17	3.09	5.17E-07	SIG	*Glyma02g15650*	*AT3G59770.1*	SAC9	sacI homology domain-containing protein / WW domain-containing protein
OIL	VIII	snp85112	17	11381352	3.23	-0.19	-0.25	1.60	5.88E-04	SUG	*Glyma17g14620*	*AT1G62790.1*		Bifunctional inhibitor / lipid-transfer protein / seed storage 2S albumin superfamily protein

PA: palmitic acid (PA); SA: stearic acid; OA: oleic acid; LA: linoleic acid; LNA: linolenic acid; OIL: oil content; Dataset I ~ VIII: the detection of main-effect QTNs for the phenotype of seed oil-related traits in NJ2011, NJ2012, NJ2014, NJ2015, NJ2016, WH2014, WH2015, and BLUP using Single-Env method of 3VmrMLM; Dataset IX: the detection of main-effect QTNs for the phenotype of seed oil-related traits across all environment using Multi-Env method of 3VmrMLM; Chr: chromosome; Pos: position; Add: additive; Dom: dominance; SIG: significant (-log10(P) ≥ 6.04); SUG: suggestion (LOD ≥ 3).

Table S10. 54 candidate gene-by-environment interactions for soybean seed oil-related traits

Trait	Marker	Chr	Pos (bp)	LOD	r^2(%)	P-value	Sign.	Gene	Homologous gene in Arabidopsis			Oil metabolism	Gene expression trend	
									Gene	Symbol	Annotation		Heat stress	Drought stress
PA	snp11768	3	5944719	25.93	1.9	8.13E-20	SIG	*Glyma03g05620*	*AT5G01650.1*	MDL2	Tautomerase / MIF superfamily protein		Up 13.94%	
PA	snp11768	3	5944719	25.93	1.9	8.13E-20	SIG	*Glyma03g05630*	*AT2G38360.1*	PRA1.B4	prenylated RAB acceptor 1.B4		Up 66.42%	
PA	snp15168	3	42150254	77.65	6.1	3.64E-74	SIG	*Glyma03g34740*	*AT3G52990.1*	PK	Pyruvate kinase family protein	Pyruvate metabolism		
PA	snp15168	3	42150254	77.65	6.1	3.64E-74	SIG	*Glyma03g34770*	*AT5G04550.1*		unknown		Down 31.62%	
PA	snp15168	3	42150254	77.65	6.1	3.64E-74	SIG	*Glyma03g34830*	*AT2G36530.1*	LOS2	Enolase			Down 25.43%
PA	snp15168	3	42150254	77.65	6.1	3.64E-74	SIG	*Glyma03g34880*	*AT1G31660.1*					Down 1.55%
PA	snp37550	8	6046707	36.66	2.72	8.33E-30	SIG	*Glyma08g08520*	*AT4G20840.1*	OGOX2	FAD-binding Berberine family protein			Down 3.88%
PA	snp37550	8	6046707	36.66	2.72	8.33E-30	SIG	*Glyma08g08610*	*AT3G58130.2*		N-acetylglucosaminylphosphatidylinositol de-N-acetylase family protein		Up 29.52%	
PA	snp51483	10	43583670	95.02	7.58	4.15E-86	SIG	*Glyma10g35381*	*AT3G53260.1*	PAL2	phenylalanine ammonia-lyase 2			Down 5.26%
PA	snp51483	10	43583670	95.02	7.58	4.15E-86	SIG	*Glyma10g35430*	*AT4G14145.1*		unknown protein		Down 5.27%	
PA	snp89203	18	7840442	36.59	2.75	9.75E-30	SIG	*Glyma18g09000*	*AT3G13080.1*	MRP3	multidrug resistance-associated protein 3			Down 4.47%
PA	snp98835	19	37280850	74.6	6.05	3.27E-66	SIG	*Glyma19g29610*	*AT5G39660.2*	CDF2	cycling DOF factor 2			Down 7.50%
SA	snp24808	5	36671535	36.95	2.71	4.46E-30	SIG	*Glyma05g31640*	*AT4G39620.2*	EMB2453	Tetratricopeptide repeat (TPR)-like superfamily protein			Down 19.01%
SA	snp24808	5	36671535	36.95	2.71	4.46E-30	SIG	*Glyma05g31670*	*AT5G64940.2*	ATH13	ABC2 homolog 13		Down 60.15%	
SA	snp25032	5	38490643	10.82	0.79	1.50E-06	SUG	*Glyma05g33910*	*AT1G73660.1*	MAPKKK	protein tyrosine kinase family protein			Down 29.23%
SA	snp25032	5	38490643	10.82	0.79	1.50E-06	SUG	*Glyma05g34000*	*AT4G02750.1*		Tetratricopeptide repeat (TPR)-like superfamily protein			Down 21.80%
SA	snp26121	6	4685918	30.48	2.34	5.08E-24	SIG	*Glyma06g06440*	*AT5G25080.1*		Sas10 / Utp3/ C1D family		Down 72.76%	
SA	snp32821	7	8047531	18.27	1.37	6.67E-13	SIG	*Glyma07g09590*						Up 60.88%
SA	snp32821	7	8047531	18.27	1.37	6.67E-13	SIG	*Glyma07g09640*	*AT3G24503.1*	ALDH2C4	aldehyde dehydrogenase 2C4		Down 31.41%	
SA	snp35491	7	35825129	16.83	1.19	1.24E-11	SIG	*Glyma07g30800*	*AT1G11380.1*		PLAC8 family protein		Down 40.19%	
SA	snp51538	10	44092582	4.62	0.33	4.62E-02	SUG	*Glyma10g35950*	*AT1G22700.2*		Tetratricopeptide repeat (TPR)-like superfamily protein		Down 6.44%	
SA	snp51538	10	44092582	4.62	0.33	4.62E-02	SUG	*Glyma10g35960*	*AT1G34430.1*	EMB3003	2-oxoacid dehydrogenases acyltransferase family protein	Pyruvate metabolism		
SA	snp51575	10	44410452	11.23	0.82	6.97E-07	SIG	*Glyma10g36180*	*AT1G22660.1*		Polynucleotide adenylyltransferase family protein		Down 37.27%	
SA	snp51575	10	44410452	11.23	0.82	6.97E-07	SIG	*Glyma10g36230*	*AT1G34270.1*	EXT2	Exostosin family protein		Up 10.56%	
SA	snp51575	10	44410452	11.23	0.82	6.97E-07	SIG	*Glyma10g36200*	*AT1G22710.1*	SUC2	sucrose-proton symporter 2			Up 63.83%
SA	snp51590	10	44469282	9.47	0.66	1.77E-05	SUG	*Glyma10g36230*	*AT1G34270.1*	EXT2	Exostosin family protein		Up 10.56%	
SA	snp51590	10	44469282	9.47	0.66	1.77E-05	SUG	*Glyma10g36370*	*AT1G14290.1*	SBH2	sphingoid base hydroxylase 2	Sphingolipid metabolism		

SA	snp51590	10	44469282	9.47	0.66	1.77E-05	SUG	*Glyma10g36200*	*AT1G22710.1*	SUC2	sucrose-proton symporter 2			Up 63.83%
SA	snp57125	12	1384742	7.15	0.5	1.00E-03	SUG	*Glyma12g02140*	*AT3G03130.1*		unknown protein			Down 3.58%
SA	snp57125	12	1384742	7.15	0.5	1.00E-03	SUG	*Glyma12g02230*	*AT5G19440.1*		NAD(P)-binding Rossmann-fold superfamily protein		Up 27.71%	
SA	snp57125	12	1384742	7.15	0.5	1.00E-03	SUG	*Glyma12g02270*	*AT3G21070.2*	NADK1	NAD kinase 1		Down 37.01%	
SA	snp57125	12	1384742	7.15	0.5	1.00E-03	SUG	*Glyma12g02290*	*AT3G21090.1*	CER5	ABC-2 type transporter family protein	Lipid export		
SA	snp57125	12	1384742	7.15	0.5	1.00E-03	SUG	*Glyma12g02330*	*AT4G10320.1*		tRNA synthetase class I (I, L, M and V) family protein			Down 23.21%
SA	snp67206	13	43130940	11.27	0.77	6.43E-07	SIG	*Glyma13g43570*	*AT1G79900.1*	BAC2	Mitochondrial substrate carrier family protein		Up 43.92%	
SA	snp94922	18	59528591	11.97	0.84	1.71E-07	SIG	*Glyma18g50340*	*AT5G39090.1*		HXXXD-type acyl-transferase family protein		Down 27.91%	
SA	snp94922	18	59528591	11.97	0.84	1.71E-07	SIG	*Glyma18g50390*	*AT1G74160.1*		unknown protein			Up 12.36%
SA	snp97034	19	12199744	37.68	2.82	9.17E-31	SIG	*Glyma19g10120*	*AT5G40510.1*		Sucrase / ferredoxin-like family protein		Down 11.75%	
SA	snp103976	20	33904632	9	0.67	4.13E-05	SUG	*Glyma20g24320*	*AT4G24175.1*		unknown protein			Down 16.63%
OA	snp11943	3	8113468	21.07	1.32	2.11E-15	SIG	*Glyma03g07460*	*AT2G38280.2*	AMPD	AMP deaminase, putative / myoadenylate deaminase, putative		Down 9.38%	
OA	snp25014	5	38379318	10.52	0.64	2.62E-06	SUG	*Glyma05g33910*	*AT1G73660.1*	MAPKKK	protein tyrosine kinase family protein			Down 29.23%
OA	snp25014	5	38379318	10.52	0.64	2.62E-06	SUG	*Glyma05g34000*	*AT4G02750.1*		Tetratricopeptide repeat (TPR)-like superfamily protein			Down 21.80%
OA	snp25644	6	1490151	13.45	0.84	1.00E-08	SIG	*Glyma06g02330*	*AT1G20330.1*	SMT2	sterol methyltransferase 2		Down 19.23%	
OA	snp36510	7	43205262	13.41	0.85	1.09E-08	SIG	*Glyma07g38460*	*AT2G15490.3*	UGT73B4	UDP-glycosyltransferase 73B4		Down 70.60%	
OA	snp36510	7	43205262	13.41	0.85	1.09E-08	SIG	*Glyma07g38510*	*AT5G19010.1*	MPK16	mitogen-activated protein kinase 16			Down 25.82%
OA	snp36510	7	43205262	13.41	0.85	1.09E-08	SIG	*Glyma07g38350*	*AT3G21570.1*		unknown protein; Has 43 Blast hits to 43 proteins in 13 species: Archae - 0; Bacteria - 0; Metazoa - 0; Fungi - 0; Plants - 43; Viruses - 0; Other Eukaryotes - 0 (source: NCBI BLink).			Up 45.62%
OA	snp47064	9	42919313	10.66	0.65	2.01E-06	SUG	*Glyma09g37450*	*AT5G15140.1*		Galactose mutarotase-like superfamily protein		Down 42.68%	
OA	snp47463	9	46005575	13.88	0.83	4.29E-09	SIG	*Glyma09g41270*	*AT3G51630.2*	WNK5	with no lysine (K) kinase 5			Up 75.43%
OA	snp51556	10	44206757	15.15	0.95	3.58E-10	SIG	*Glyma10g35950*	*AT1G22700.2*		Tetratricopeptide repeat (TPR)-like superfamily protein		Down 6.44%	
OA	snp51556	10	44206757	15.15	0.95	3.58E-10	SIG	*Glyma10g35960*	*AT1G34430.1*	EMB3003	2-oxoacid dehydrogenases acyltransferase family protein	Pyruvate metabolism		
OA	snp51573	10	44405881	17.16	1.02	6.37E-12	SIG	*Glyma10g36180*	*AT1G22660.1*		Polynucleotide adenylyltransferase family protein		Down 37.27%	
OA	snp51573	10	44405881	17.16	1.02	6.37E-12	SIG	*Glyma10g36230*	*AT1G34270.1*	EXT2	Exostosin family protein		Up 10.56%	
OA	snp51573	10	44405881	17.16	1.02	6.37E-12	SIG	*Glyma10g36200*	*AT1G22710.1*	SUC2	sucrose-proton symporter 2			Up 63.83%
LA	snp11943	3	8113468	22.47	1.52	1.15E-16	SIG	*Glyma03g07460*	*AT2G38280.2*	AMPD	AMP deaminase, putative / myoadenylate deaminase, putative		Down 9.38%	
LA	snp25032	5	38490643	13.3	0.87	1.33E-08	SIG	*Glyma05g33910*	*AT1G73660.1*	MAPKKK	protein tyrosine kinase family protein			Down 29.23%
LA	snp25032	5	38490643	13.3	0.87	1.33E-08	SIG	*Glyma05g34000*	*AT4G02750.1*		Tetratricopeptide repeat (TPR)-like superfamily protein			Down 21.80%
LA	snp26465	6	7393451	29.81	1.95	3.79E-27	SIG	*Glyma06g09810*	*AT5G49700.1*	AHL17	Predicted AT-hook DNA-binding family protein		Up 24.22%	
LA	snp33280	7	10409939	11.19	0.73	7.50E-07	SIG	*Glyma07g12130*	*AT3G17770.1*		Dihydroxyacetone kinase	Glycerolipid metabolism		

LA	snp33280	7	10409939	11.19	0.73	7.50E-07	SIG	*Glyma07g12150*	*AT2G01930.2*	BPC1	basic pentacysteine1		Up 3.02%	
LA	snp39463	8	18183299	8.69	0.55	7.15E-05	SUG	*Glyma08g23900*	*AT1G51660.1*	MKK4	mitogen-activated protein kinase kinase 4		Down 30.55%	
LA	snp39463	8	18183299	8.69	0.55	7.15E-05	SUG	*Glyma08g23950*	*AT1G16860.1*		Ubiquitin-specific protease family C19-related protein			Down 5.58%
LA	snp39463	8	18183299	8.69	0.55	7.15E-05	SUG	*Glyma08g23860*	*AT1G17745.1*	PGDH	D-3-phosphoglycerate dehydrogenase			Down 22.97%
LA	snp51590	10	44469282	7.4	0.49	6.54E-04	SUG	*Glyma10g36230*	*AT1G34270.1*	EXT2	Exostosin family protein		Up 10.56%	
LA	snp51590	10	44469282	7.4	0.49	6.54E-04	SUG	*Glyma10g36370*	*AT1G14290.1*	SBH2	sphingoid base hydroxylase 2	Sphingolipid metabolism		
LA	snp51590	10	44469282	7.4	0.49	6.54E-04	SUG	*Glyma10g36200*	*AT1G22710.1*	SUC2	sucrose-proton symporter 2			Up 63.83%
LA	snp85477	17	13741950	29.31	2.08	6.22E-23	SIG	*Glyma17g16950*	*AT5G65760.1*		Serine carboxypeptidase S28 family protein		Up 43.15%	
OIL	snp52940	11	2571951	6.47	0.79	2.28E-04	SUG	*Glyma11g03700*	*AT1G09620.1*		ATP binding;leucine-tRNA ligases;aminoacyl-tRNA ligases;nucleotide binding;ATP binding;aminoacyl-tRNA ligases			Down 17.02%
OIL	snp63233	13	14402337	6.43	0.81	2.47E-04	SUG	*Glyma13g11700*	*AT2G04350.2*	LACS8	AMP-dependent synthetase and ligase family protein	Fatty acid biosynthesis		
OIL	snp83910	17	1802351	9.11	1.17	1.37E-06	SUG	*Glyma17g02620*	*AT4G15640.1*		unknown protein			Down 15.69%
OIL	snp83910	17	1802351	9.11	1.17	1.37E-06	SUG	*Glyma17g02640*	*AT5G18620.2*	CHR17	chromatin remodeling factor17		Down 58.23%	

PA: palmitic acid (PA); SA: stearic acid; OA: oleic acid; LA: linoleic acid; LNA: linolenic acid; OIL: oil content; Chr: chromosome; Pos: position; Add: additive; Dom: dominance; SIG: significant (-log10(P) ≥ 6.04); SUG: suggestion (LOD ≥ 3); Up: up regulation; Down: down regulation.

Table S11. KEGG pathway enrichment analysis for each module of co-expression network under drought stress

Module	KEGG pathway	ID	Input number	Background number	P-Value	Corrected P-Value	Genes
blue	Plant hormone signal transduction	gmx04075	16	675	2.12E-04	1.71E-02	Glyma10g28290, Glyma11g34410, Glyma08g03780, Glyma12g17510, Glyma03g38391, Glyma07g33600, Glyma02g14880, Glyma05g35830, Glyma17g05540, Glyma03g31520, Glyma20g35280, Glyma06g43620, Glyma04g04170, Glyma08g16550, Glyma19g11770, Glyma17g12080
brown	Base excision repair	gmx03410	4	68	7.15E-05	2.43E-03	Glyma15g30150, Glyma08g25090, Glyma15g41940, Glyma03g14075
brown	Brassinosteroid biosynthesis	gmx00905	2	23	2.62E-03	2.24E-02	Glyma18g03211, Glyma19g04250
turquoise	Ribosome	gmx03010	97	592	2.46E-47	1.56E-44	Glyma08g25990, Glyma01g01220, Glyma07g04890, Glyma03g39480, Glyma03g32380, Glyma03g37340, Glyma16g03170, Glyma01g05740, Glyma05g01180, Glyma05g35030, Glyma10g05580, Glyma18g13270, Glyma05g27940, Glyma09g34760, Glyma13g39270, Glyma16g08440, Glyma11g11040, Glyma13g19650, Glyma20g35000, Glyma15g13650, Glyma19g29410, Glyma19g36180, Glyma02g42260, Glyma08g41280, Glyma08g04990, Glyma03g06440, Glyma15g18610, Glyma04g16660, Glyma07g01540, Glyma10g02060, Glyma10g36880, Glyma03g25520, Glyma11g25910, Glyma08g28800, Glyma20g28780, Glyma18g43721, Glyma04g40200, Glyma04g36140, Glyma12g08050, Glyma13g06920, Glyma10g37840, Glyma12g07160, Glyma15g00610, Glyma20g38080, Glyma10g06040, Glyma07g31840, Glyma03g36420, Glyma14g06630, Glyma20g30810, Glyma16g01460, Glyma02g43080, Glyma06g14330, Glyma01g44700, Glyma11g05160, Glyma05g26290, Glyma04g16890, Glyma08g46850, Glyma18g52310, Glyma02g05920, Glyma12g32850, Glyma18g02970, Glyma01g45060, Glyma03g35530, Glyma10g29170, Glyma03g33460, Glyma02g02140, Glyma05g28880, Glyma03g40530, Glyma02g05370, Glyma13g23400, Glyma02g04400, Glyma11g00450, Glyma05g27570, Glyma13g31650, Glyma18g01110, Glyma03g33530, Glyma05g26320, Glyma11g15230, Glyma19g03520, Glyma19g43190, Glyma15g42150, Glyma19g39940, Glyma18g32680, Glyma15g23220, Glyma01g31270, Glyma15g10220, Glyma01g00740, Glyma01g03570, Glyma15g08210, Glyma13g31130, Glyma10g39050, Glyma15g02610, Glyma15g10210, Glyma14g38950, Glyma08g10910, Glyma03g40110, Glyma09g02790
turquoise	Ribosome biogenesis in eukaryotes	gmx03008	28	159	2.80E-15	5.58E-13	Glyma16g04570, Glyma20g35750, Glyma05g03150, Glyma11g12200, Glyma17g09070, Glyma13g00880, Glyma15g06450, Glyma13g32840, Glyma17g13770, Glyma10g24060, Glyma08g47440, Glyma05g37950, Glyma08g15130, Glyma17g35220, Glyma08g01650, Glyma03g34360, Glyma17g09690, Glyma17g18400, Glyma17g03310, Glyma12g04400, Glyma04g37770, Glyma17g01570, Glyma01g00460, Glyma14g09940, Glyma02g39510, Glyma03g37300, Glyma03g32150, Glyma18g02340
turquoise	DNA replication	gmx03030	21	99	3.55E-13	3.74E-11	Glyma11g19880, Glyma07g36680, Glyma13g22420, Glyma08g08920, Glyma17g11220,

							Glyma12g08600, Glyma11g12110, Glyma16g34470, Glyma05g03440, Glyma19g40370, Glyma11g37330, Glyma04g38800, Glyma08g45120, Glyma15g16570, Glyma11g18960, Glyma14g02860, Glyma17g03920, Glyma03g37770, Glyma02g45610, Glyma20g24590, Glyma12g04320
turquoise	Protein processing in endoplasmic reticulum	gmx04141	22	375	1.18E-04	2.08E-03	Glyma16g08410, Glyma06g17060, Glyma05g36620, Glyma08g02940, Glyma02g43460, Glyma18g35976, Glyma06g12090, Glyma08g46410, Glyma14g01100, Glyma05g36600, Glyma12g04950, Glyma01g09310, Glyma03g37650, Glyma07g04860, Glyma20g23080, Glyma04g38000, Glyma14g05520, Glyma18g01416, Glyma02g13790, Glyma18g52480, Glyma01g01270, Glyma10g28890
turquoise	RNA degradation	gmx03018	14	203	4.45E-04	6.13E-03	Glyma20g19980, Glyma10g25630, Glyma20g31120, Glyma03g34830, Glyma07g30290, Glyma08g06950, Glyma04g09180, Glyma08g05480, Glyma19g37270, Glyma08g21370, Glyma04g04300, Glyma02g13980, Glyma09g18490, Glyma02g12480
turquoise	Base excision repair	gmx03410	7	68	1.50E-03	1.70E-02	Glyma11g19880, Glyma14g02860, Glyma12g08600, Glyma16g34470, Glyma04g38800, Glyma08g45120, Glyma11g18960
turquoise	Mismatch repair	gmx03430	7	75	2.51E-03	2.55E-02	Glyma11g19880, Glyma05g03440, Glyma12g08600, Glyma17g13050, Glyma04g38800, Glyma08g45120, Glyma20g24590
turquoise	Nucleotide excision repair	gmx03420	9	123	3.09E-03	2.71E-02	Glyma11g19880, Glyma14g02860, Glyma12g08600, Glyma16g34470, Glyma05g03440, Glyma04g38800, Glyma08g45120, Glyma11g18960, Glyma20g24590
turquoise	Starch and sucrose metabolism	gmx00500	15	299	5.45E-03	4.16E-02	Glyma01g43650, Glyma03g37420, Glyma11g04210, Glyma11g10130, Glyma14g04940, Glyma14g39930, Glyma10g38570, Glyma18g10370, Glyma15g20180, Glyma10g15980, Glyma12g36870, Glyma08g12020, Glyma06g47630, Glyma02g01990, Glyma15g03620

Table S12. KEGG pathway enrichment analysis for each module of co-expression network under control condition

Module	KEGG pathway	ID	Input number	Background number	P-Value	Corrected P-Value	Genes
blue	Plant hormone signal transduction	gmx04075	20	675	4.71E-04	2.41E-02	Glyma17g07240, Glyma13g01150, Glyma17g07280, Glyma13g16640, Glyma17g07250, Glyma02g09550, Glyma20g26290, Glyma13g01140, Glyma07g33600, Glyma06g04353, Glyma11g34410, Glyma09g08290, Glyma05g35830, Glyma08g03780, Glyma17g12080, Glyma19g31320, Glyma04g04050, Glyma17g07270, Glyma15g19840, Glyma12g03490
green	DNA replication	gmx03030	13	99	4.55E-18	3.06E-16	Glyma11g19880, Glyma07g36680, Glyma13g22420, Glyma08g08920, Glyma17g11220, Glyma11g12110, Glyma19g40370, Glyma11g37330, Glyma08g45120, Glyma17g03920, Glyma03g37770, Glyma02g45610, Glyma12g04320
green	Protein processing in endoplasmic reticulum	gmx04141	15	375	1.77E-13	3.90E-12	Glyma16g33130, Glyma05g36600, Glyma04g38000, Glyma13g10700, Glyma08g02940, Glyma05g36620, Glyma17g03520, Glyma06g17060, Glyma08g02960, Glyma10g28890, Glyma14g40320, Glyma08g07330, Glyma03g37650, Glyma20g16070, Glyma17g37820
green	Protein export	gmx03060	4	92	1.27E-04	1.15E-03	Glyma05g36620, Glyma08g02940, Glyma05g36600, Glyma08g02960
green	Phagosome	gmx04145	3	164	9.95E-03	4.94E-02	Glyma10g28890, Glyma06g17060, Glyma04g38000
red	Galactose metabolism	gmx00052	3	103	1.49E-03	2.61E-02	Glyma08g21370, Glyma20g22700, Glyma19g40680
turquoise	Ribosome	gmx03010	78	592	9.41E-32	5.83E-29	Glyma20g35000, Glyma10g06040, Glyma08g25990, Glyma07g31840, Glyma02g05370, Glyma13g23400, Glyma03g36420, Glyma19g36180, Glyma02g04400, Glyma01g05740, Glyma01g01220, Glyma11g00450, Glyma15g13650, Glyma20g30810, Glyma01g31270, Glyma02g08690, Glyma15g00610, Glyma18g13270, Glyma14g38950, Glyma08g41280, Glyma16g01460, Glyma13g06920, Glyma08g04990, Glyma03g06440, Glyma03g39480, Glyma19g03520, Glyma15g18610, Glyma10g36880, Glyma02g43080, Glyma19g39940, Glyma01g45060, Glyma03g37340, Glyma16g03170, Glyma01g44700, Glyma15g42150, Glyma06g14330, Glyma08g46850, Glyma20g38080, Glyma03g33530, Glyma15g23220, Glyma05g27940, Glyma11g11040, Glyma03g25520, Glyma11g25910, Glyma11g05160, Glyma13g31130, Glyma04g16890, Glyma01g00740, Glyma05g01180, Glyma04g40200, Glyma08g28800, Glyma15g41870, Glyma18g52310, Glyma02g05920, Glyma12g32850, Glyma12g08050, Glyma15g08210, Glyma03g35530, Glyma20g28780, Glyma18g02970, Glyma18g43721, Glyma12g07160, Glyma05g26290, Glyma03g40530, Glyma19g29410, Glyma10g29170, Glyma13g39270, Glyma03g32380,

							Glyma05g27570, Glyma04g16660, Glyma02g02140, Glyma18g32680, Glyma08g10910, Glyma05g28880, Glyma10g37840, Glyma10g39050, Glyma09g02790, Glyma13g19650
turquoise	Ribosome biogenesis in eukaryotes	gmx03008	27	159	3.24E-14	1.00E-11	Glyma16g04570, Glyma05g03150, Glyma11g12200, Glyma20g17960, Glyma17g09070, Glyma13g00880, Glyma15g06450, Glyma13g32840, Glyma02g44360, Glyma17g13770, Glyma14g36760, Glyma10g24060, Glyma08g47440, Glyma07g03646, Glyma08g15130, Glyma05g05620, Glyma08g01650, Glyma03g34360, Glyma17g09690, Glyma02g08350, Glyma12g04400, Glyma04g37770, Glyma17g01570, Glyma01g00460, Glyma02g39510, Glyma03g37300, Glyma03g32150
turquoise	RNA degradation	gmx03018	13	203	1.61E-03	3.84E-02	Glyma20g19980, Glyma10g25630, Glyma03g34830, Glyma07g30290, Glyma08g06950, Glyma04g09180, Glyma08g05480, Glyma06g23081, Glyma04g04300, Glyma02g13980, Glyma09g18490, Glyma13g42990, Glyma02g12480
pink	Protein processing in endoplasmic reticulum	gmx04141	4	375	4.75E-04	1.31E-02	Glyma02g43460, Glyma14g01100, Glyma20g23080, Glyma14g05520
pink	Ribosome biogenesis in eukaryotes	gmx03008	2	159	1.04E-02	4.41E-02	Glyma18g02340, Glyma17g35220
yellow	Base excision repair	gmx03410	4	68	7.24E-04	1.59E-02	Glyma15g30150, Glyma08g25090, Glyma15g41940, Glyma03g14075
yellow	Protein processing in endoplasmic reticulum	gmx04141	8	375	1.51E-03	2.54E-02	Glyma18g52461, Glyma18g01416, Glyma12g12600, Glyma18g52480, Glyma04g14250, Glyma02g41150, Glyma16g01440, Glyma07g04860
magenta	Ribosome	gmx03010	22	592	3.40E-30	1.63E-28	Glyma02g42260, Glyma07g04890, Glyma15g07420, Glyma13g31650, Glyma18g01110, Glyma05g26320, Glyma11g15230, Glyma03g32380, Glyma19g43190, Glyma05g35030, Glyma10g05580, Glyma15g10220, Glyma14g06630, Glyma07g01540, Glyma01g03570, Glyma10g02060, Glyma09g34760, Glyma04g36140, Glyma15g02610, Glyma15g10210, Glyma05g28880, Glyma03g40110

Table S13. Candidate genes available in future soybean breeding

Gene	% in bred soybean	Haplotype	Position of haplotype
Glyma02g15650	20.00	CG	14142249, 14142849
Glyma07g09370	45.45	CCGGAGGGGCCGGGGC	7804702, 7804735, 7804770, 7804809, 7805051, 7805088, 7805110, 7805133, 7805202, 7805235, 7805243, 7805302, 7805602, 7805811, 7806463, 7806818
Glyma08g13290	35.90	ATCAT	9715686, 9715732, 9715903, 9718958, 9723962
Glyma08g45990	35.14	CAG	45222088, 45222725, 45223806
Glyma10g05750	7.89	GC	4497492, 4497520
Glyma16g02090	28.95	TACAAC	1607208, 1607293, 1607592, 1607743, 1607839, 1608107
Glyma17g14620	18.42	GGAAGAAAAA	11361524, 11361739, 11361768, 11363356, 11363586, 11363814, 11365474, 11365569, 11365581, 11365777
Glyma17g14810	38.24	TGATAT	11566581, 11566627, 11567353, 11567841, 11568109, 11568170
